# Deep learning models map rapid plant species changes from citizen science and remote sensing data

**DOI:** 10.1073/pnas.2318296121

**Published:** 2024-09-05

**Authors:** Lauren E. Gillespie, Megan Ruffley, Moises Exposito-Alonso

**Affiliations:** ^a^Department of Plant Biology, Carnegie Science, Stanford, CA 94305; ^b^Department of Computer Science, Stanford University, Stanford, CA 94305; ^c^Department of Integrative Biology, University of California, Berkeley, CA 94720; ^d^Department of Biology, Stanford University, Stanford, CA 94305; ^e^Department of Global Ecology, Carnegie Science, Stanford, CA 94305; ^f^HHMI, University of California, Berkeley, CA 94720

**Keywords:** deep learning, biodiversity change, remote sensing, species distribution models

## Abstract

Anthropogenic habitat destruction and climate change are reshaping the geographic distribution of plants worldwide. However, we are still unable to map species shifts at high spatial, temporal, and taxonomic resolution. Here, we develop a deep learning model trained using remote sensing images from California paired with half a million citizen science observations that can map the distribution of over 2,000 plant species. Our model—*Deepbiosphere—*not only outperforms many common species distribution modeling approaches (AUC 0.95 vs. 0.88) but can map species at up to a few meters resolution and finely delineate plant communities with high accuracy, including the pristine and clear-cut forests of Redwood National Park. These fine-scale predictions can further be used to map the intensity of habitat fragmentation and sharp ecosystem transitions across human-altered landscapes. In addition, from frequent collections of remote sensing data, *Deepbiosphere* can detect the rapid effects of severe wildfire on plant community composition across a 2-y time period. These findings demonstrate that integrating public earth observations and citizen science with deep learning can pave the way toward automated systems for monitoring biodiversity change in real-time worldwide.

## Significance

Worldwide, plant biodiversity is changing rapidly due to habitat destruction and a warming climate. However, we lack methods at high enough spatial and temporal resolution to detect these changes for individual species. Here, we develop a deep learning-based approach trained with citizen science data that detects thousands of plant species from satellite or aerial imagery. We show how this approach can detect individual species at meter-resolution in California and can detect rapid changes in the makeup of plant communities across both space and time. Our approach provides an efficient way to map plant biodiversity from above that is easily scalable to a global system for monitoring plant biodiversity.

Humans are impacting plant biodiversity worldwide (1, 2), affecting critical ecosystem services such as carbon sequestration (3), primary productivity (4), and climate regulation (5). Major drivers include climate warming, which shifts plant ranges toward the poles and peaks over decades (2, 6) and land use change, which converts hundreds of thousands of hectares of habitat each year (7). Even largely undisturbed habitats are often still undergoing marked change at the individual species level (8). Therefore, comprehensively monitoring plant biodiversity will require tracking individual species at high-resolution in both space and time, a challenging and hard to solve task (9–11). Such high spatial and temporal resolution plant species maps will be crucial to tracking the world’s progress toward the United Nations’ Global Biodiversity Framework goal of protecting 30% of the world’s biodiversity by 2030 (12).

Deep learning has shown remarkable ability to make sense of large-scale, noisy datasets from across the life and earth sciences, from protein folding (13) to climate modeling (14). To help close the gap in mapping plant species at high spatiotemporal resolution (15), here, we take a similar data-driven, deep learning-based approach, and train deep neural networks to predict the presence of thousands of plant species simultaneously from large-scale citizen science, climate, and remote sensing datasets. We showcase how these deep neural networks can generate fine-scale maps of thousands of plant species from meter-resolution remote sensing imagery. We further demonstrate that these maps are high enough resolution in both space and time to detect anthropogenic signatures of biodiversity change, including deforestation, habitat fragmentation, and severe wildfire. Relying solely on publicly available data, our approach is easily scalable to entire continents and paves the way for automated plant biodiversity monitoring tools at global-scale.

**Fig. 1.**
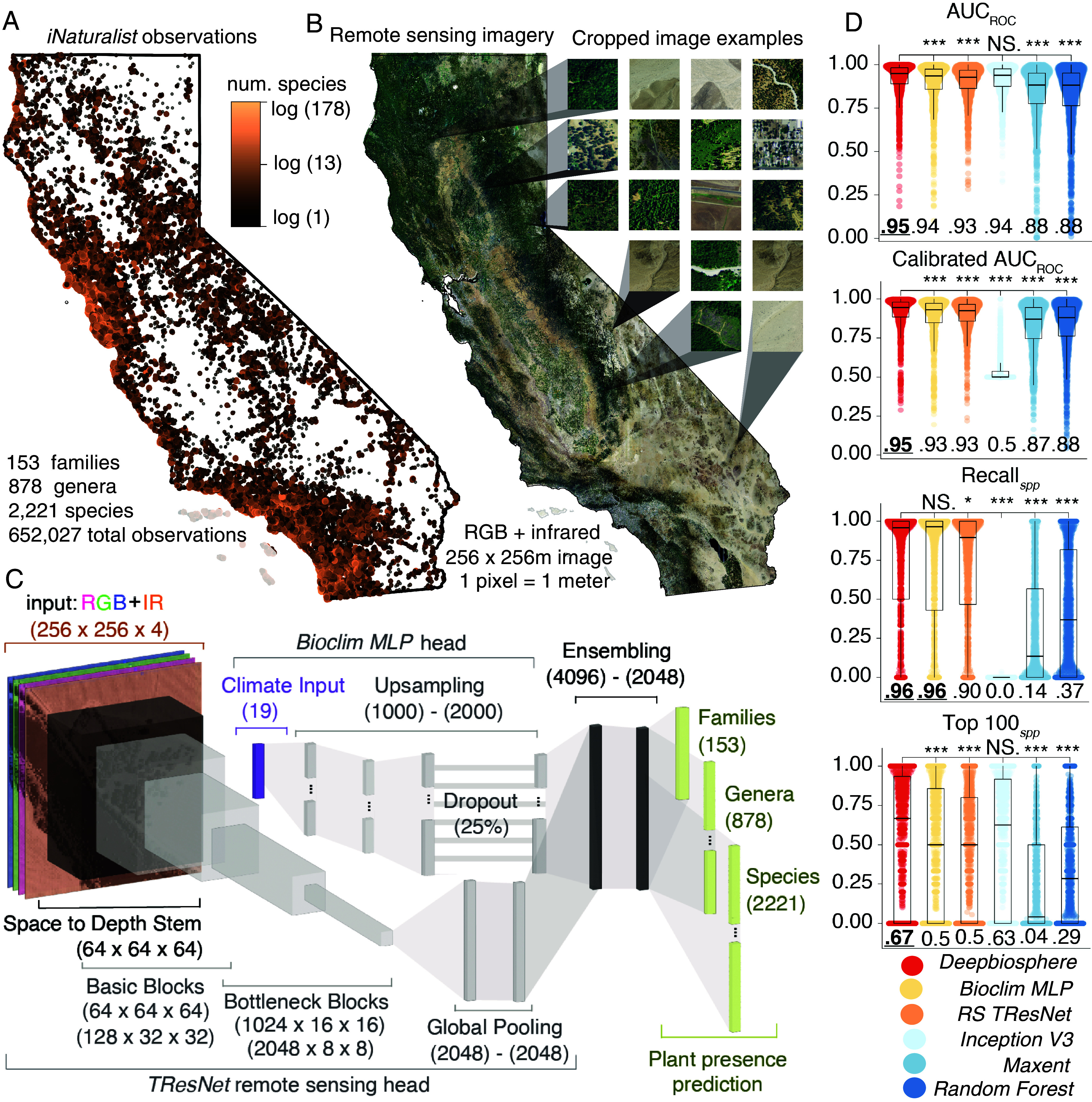
Training a deep neural network to predict the presence of thousands of plant species. (*A*) Map of over 650,000 *iNaturalist* observations for 2,221 plant species we curated to train plant species distribution models (SDMs) (24). (*B*) To train the CNNs, species observations were linked to 256 × 256 m remote sensing images cropped from 2012 NAIP data (16), along with climate variables (31) (*SI Appendix*, Fig. S2). (*C*) *Deepbiosphere* architecture, which combines a residual CNN (*TResNet*) (27) trained using remote sensing imagery with a multilayered perceptron (*MLP*) network (32) trained using climate variables to predict plant families, genera, and species (*SI Appendix*, Table S3). Layer types and dimensions are annotated for each section of the neural network. (*D*) Comparison of *Deepbiosphere’s* performance to common climate-based SDMs including *Maxent*, *Random Forest* (33), as well as the *Bioclim MLP* head trained with just climate, the remote sensing *TResNet* head trained with just NAIP data, and an *Inception V3* model from previous work (34). Metrics are reported per-species for the 1,541 species shared between the uniform split of the training and testing set, with the median score annotated on each boxplot. *spp* = per-species, AUC = area under the curve; ROC = receiver operating characteristic curve; RS = remote sensing. Stars indicate results from unpaired student’s *t* test, with *** indicating a *P*-value < 10^−3^, * indicating a *P*-value < 10^−1^, and NS. indicating a nonsignificant *P*-value of > 0.1.

To develop deep neural networks that predict the presence of thousands of plant species from high-resolution remote sensing imagery, we focused on California, a species-rich and data-dense state with abundant high-quality remote sensing imagery (16), dense citizen science observations [~2 million species occurrences since the year 2000 (17)], and a variety of independently generated ecosystem measurements and maps to serve as ground truth (18–21). First, we compiled almost one million observations from the Global Biodiversity Information Facility (22), filtering out duplicate observations, low coverage species, and oversampled areas to curate a large dataset of over 650,000 research-grade, primarily *iNaturalist,* citizen science observations for 2,221 vascular plant species (23, 24) (Fig. 1*A* and *SI Appendix*, SM 1.1). Similar to previous datasets (25), we paired each observation’s species label with the location’s corresponding 256 × 256 pixel, 1-m-resolution RGB-Infrared aerial image from the National Agricultural Imagery Program (NAIP) (16) (Fig. 1*B* and *SI Appendix*, SM 1.4, Fig. S2, and Table S1). For extracting statistical patterns from this high-resolution imagery, we employed convolutional neural networks (CNNs) (26), specifically, a multilabel-optimized, residual CNN architecture (*RS TResNet,* *SI Appendix*, SM 3.2.1 and Table S2) (27). To improve performance for species with few observations, we modified this architecture to classify both species, genus, and family to help share signals of niche similarity for species that are both phylogenetically related (28) and occupy similar ranges, which outperformed CNNs trained with just species labels (*SI Appendix*, Table S6). We further included co-occurring species information through neighbor imputation (*SI Appendix*, SM 1.2 and Fig. S2) which also improved CNN performance (*SI Appendix*, Table S6), matching previous work, and expectations from community ecology (29, 30) (see *SI Appendix*, SM 1.2 for options to train models without these additional information).

To improve learning from noisy citizen science data—which possesses systematic user observation biases, like overrepresenting common species and species in densely populated regions (35) (*SI Appendix*, Fig. S3)—we developed a sampling bias-aware loss function [sampling-aware binary cross-entropy (BCE), *SI Appendix*, SM 3.2.2]. Most locations in our dataset contain an incomplete list of present species because many plants simply have not yet been observed and uploaded to *iNaturalist* (*SI Appendix*, Fig. S3*B*). To combat this, our new loss function differentially downweighs the information contributed from absent species based on the estimated per-location incompleteness of the species presence data. Across a suite of twenty different accuracy metrics using held-out test examples (*SI Appendix*, SM 1.3.1 and 2 and Fig. S4*A*), our modified *TResNet* and sampling-aware loss function outperformed a range of common loss functions (*SI Appendix*, Table S7), reinforcing how incorporating information about known biases into the learning process improves species distribution modeling (36).

Both climate and land cover are key drivers of plant range limits, but at different spatial scales (37). Climate data such as WorldClim’s Bioclim variables [19 composite climate variables chosen for their biological relevance and averaged across 1970 to 2000 from monthly precipitation and temperature averages (31)] are common predictors for species distribution models (SDMs) (29, 33), and we were curious how performance would vary when training deep neural networks only with climate variables, only with remote sensing imagery, or with both. To do so, we modified a feed-forward multilayer perceptron (MLP) deep learning architecture to predict species from bioclimatic data (32) (*Bioclim MLP*, *SI Appendix*, SM 3.3.3 and Table S5). Surprisingly, this simple climate-based MLP outperformed our remote sensing-based *TResNet* CNN for several accuracy metrics (*SI Appendix*, Table S8), so next we sought to develop a deep learning architecture that could utilize both types of data together.

Since most remote sensing data and climate variables are of much different spatial resolutions (*SI Appendix*, Fig. S1), we designed a unique neural network architecture to process both data streams simultaneously by combining our *Bioclim MLP* with our modified *TResNet* (Fig. 1*C* and *SI Appendix*, SM 3.2.3 and Table S3). For this multiheaded architecture—which we call *Deepbiosphere*—we see that for species prediction the sum is greater than the parts, as *Deepbiosphere* largely outperformed both our *Bioclim MLP* trained only with climate data and our modified *TResNet* trained only with remote sensing data (Table 1 and *SI Appendix*, Table S8). *Deepbiosphere* further outperformed the classical climate-based species distribution modeling methods *Random Forest* and *Maximum Entropy* (*Maxent*) (Table 1 and *SI Appendix*, SM 3.3 and Fig. S8), and a previously published CNN trained with remote sensing data using a standard computer vision loss function and single-label training paradigm (34) (*Inception V3,* Table 1 and *SI Appendix*, SM 3.2.4 and Tables S4 and S8). Per-species, *Deepbiosphere’s* performance increased across a wide range of accuracy metrics (*SI Appendix*, Figs. S5 and S6), including the area under the receiver operator characteristic curve (AUC_ROC_, *SI Appendix*, SM 2.2), a metric of the model’s discrimination ability across a gradient of presence–absence thresholds, alongside binary classification metrics using a standard presence–absence threshold of 0.5 (*SI Appendix*, SM 2.1). For the 1,541 species tested, *Deepbiosphere* improved the mean AUC_ROC_ by 1 to 7% compared to all nontrivial baselines (Fig. 1*D* and *SI Appendix*, Fig. S5), especially including the rarest species (*SI Appendix*, Table S9). While these individual accuracy improvements may seem small and some species—especially rare ones—are predicted poorly (*SI Appendix*, Fig. S6), *Deepbiosphere* importantly exhibits consistent and improved performance on all types of accuracy metrics—including binary classification metrics, which are important when drawing species range maps (Precision*_spp_*: 0.2 to 1.3% improvement; Recall_obs_: 0.0 to 100.%; F1*_spp_*: 0.4 to 2.6%, Presence Accuracy: 1.0 to 89.5%); discrimination metrics, which are important for calibrating model’s performance across presence thresholds (AUC_PRC_: 0.4 to 2.2% improvement; AUC_ROC_: 1.1 to 6.8%); and ranking metrics, which are important for understanding models’ confidence across species (Top–100*_spp_*: 4.2 to 62.5% improvement; Top–100_obs_: 0.8 to 47.%) (Table 1).

**Table 1.** Comparing the accuracy of SDMs on unseen examples. Bolded entries refer to the top performing model for a given accuracy metric

Model name	Data	Res.	Loss	AUC_ROC_	AUC_PRC_	Recall_obs_	Recall*_spp_*	Prec*_spp_*	F1*_spp_*	Pres. Acc.	Top 100_obs_	Top 100*_spp_*
*Deepbiosphere*	Remote sensing + Climate	256 m	Sampling-aware BCE	**0.9496 [0.89 to 0.98]**	**0.0398 [0.01 to 0.11]**	**1.0 [0.89 to 1.0]**	0.9583 [0.5 to 1.0]	**0.0131 [0.004 to 0.04]**	**0.0258 [0.01 to 0.07]**	**0.8918**	**0.7613**	**0.6667 [0.0 to 0.93]**
*Bioclim MLP*	Climate	~1,000 m	Sampling-aware BCE	0.9346 [0.86 to 0.98]	0.0346 [0.01 to 0.10]	**1.0 [0.86 to 1.0]**	**0.9643 [0.43 to 1.0]**	0.0111 [0.002 to 0.03]	0.0218 [0.005 to 0.06]	0.8820	0.7035	0.5 [0.0 to 0.86]
*RS TResNet*	Remote sensing	256 m	Sampling-aware BCE	0.9268 [0.86 to 0.97]	0.0265 [0.01 to 0.08]	**1.0 [0.83 to 1.0]**	0.8958 [0.47 to 1.0]	0.01 [0.003 to 0.03]	0.0198 [0.01 to 0.05]	0.8645	0.6779	0.5 [0.0 to 0.8]
*Inception V3* (34)	Remote sensing	256 m	CE	0.9391 [0.88 to 0.99]	0.0359 [0.01 to 0.10]	0.0 [0.0 to 0.0]	0.0 [0.0 to 0.0]	0.0 [0.0 to 0.0]	0.0 [0.0 to 0.0]	0.0013	0.7533	0.625 [0.0 to 0.92]
*Maxent* (33)	Climate	~1,000 m	N/A	0.8825 [0.78 to 0.95]	0.018 [0.004 to 0.07]	0.0 [0.0 to 0.5]	0.1348 [0.0 to 0.57]	0.0048 [0.0 to 0.03]	0.0089 [0.0 to 0.06]	0.2761	0.2910	0.0417 [0.0 to 0.5]
*Random Forest* (33)	Climate	~1,000 m	N/A	0.882 [0.76 to 0.95]	0.0237 [0.004 to 0.09]	0.2821 [0.0 to 0.88]	0.3684 [0.0 to 0.82]	0.0086 [0.0 to 0.04]	0.0166 [0.0 to 0.07]	0.3943	0.3709	0.2857 [0.0 to 0.60]
*Random*	N/A	NA	N/A	0.4995 [0.48 to 0.52]	0.0022 [0.001 to 0.006]	0.5 [0.4 to 0.6]	0.5 [0.47 to 0.53]	0.0016 [0.001 to 0.01]	0.0031 [0.001 to 0.01]	0.5005	0.0451	0.0333 [0.0 to 0.07]
*Frequency*	N/A	NA	N/A	0.5 [0.5 to 0.5]	0.0016 [0.001 to 0.01]	0.0 [0.0 to 0.0]	0.0 [0.0 to 0.0]	0.0 [0.0 to 0.0]	0.0 [0.0 to 0.0]	0.0656	0.1952	0.0 [0.0 to 0.0]

Median [IQR] are reported for each accuracy metric and for each species distribution model along with baseline random and frequency-based estimations. Examples used for evaluation were sampled from across all of California and were at least 1.3 km away from any training point (*SI Appendix*, SM 1.3.1 and Fig. S4*A*). For more reported accuracy metrics, see *SI Appendix*, Table S8. Res. = Resolution; *MLP* = multilayer perceptron; BCE = binary cross-entropy; CE = cross-entropy; *spp* = per-species; obs = per-observation; AUC_ROC_ = area under receiver operating curve; AUC_PRC_ = area under precision–recall curve; Prec = precision; Pres. Acc. = Presence Accuracy.

To test *Deepbiosphere’s* ability to extrapolate to previously unseen regions, a 10-fold latitudinal block-based cross-validation experiment was also performed (*SI Appendix*, SM 1.3.2 and Fig. S4*B*). *Deepbiosphere* exhibited a significant increase in accuracy across all metrics (*P*-values < 0.025, except for *Random Forest* Recall*_spp_*; Table 2 and *SI Appendix*, Fig. S7 and Table S10), supporting an improved extrapolation ability to geographic areas excluded from training. While accuracy did decrease in regions with fewer training observations per-region, the overall decrease was less imbalanced than the original training data, implying that there is significant transfer in predictability from well-sampled ecosystems to data-sparse ones with our approach (*SI Appendix*, Fig. S3 *D* and *E*). These results suggest that combining remote sensing and climate information with deep learning improves the joint species distribution modeling of plants across a wide range of taxa and heterogeneous landscapes.

**Table 2.** Comparing the accuracy of selected SDMs on held-out cross-validation blocks. Bolded entries refer to the top performing model for a given accuracy metric

Model name	Data	Res.	AUC_ROC_	AUC_PRC_	Recall_img_	Recall*_spp_*	Prec*_spp_*	F1*_spp_*	Pres. Acc.	Top 100_img_	Top 100*_spp_*
*Deepbiosphere*	Remote sensing + Climate	256 m	**0.8682 [0.84 to 0.88]**	**0.0365 [0.03 to 0.04]**	**0.8571 [0.82 to 0.88]**	**0.5865 [0.54 to 0.64]**	**0.0219 [0.02 to 0.029]**	**0.0414 [0.04 to 0.05]**	**0.8425 [0.83 to 0.87]**	**0.6803 [0.67 to 0.69]**	**0.2242 [0.16 to 0.29]**
*Climate MLP*	Climate	~1,000 m	0.8025 [0.77 to 0.82]	0.0279 [0.026 to 0.03]	0.8091 [0.76 to 0.85]	0.4536 [0.38 to 0.50]	0.0129 [0.01 to 0.017]	0.0242 [0.02 to 0.03]	0.7856 [0.75 to 0.82]	0.5482 [0.53 to 0.58]	0.0378 [0.0 to 0.07]
*Maxent* (33)	Climate	~1,000 m	0.7339 [0.71 to 0.77]	0.0207 [0.02 to 0.024]	0.4273 [0.32 to 0.68]	0.1541 [0.00 to 0.51]	0.0045 [0.0 to 0.011]	0.0088 [0.0 to 0.021]	0.4268 [0.36 to 0.64]	0.1862 [0.16 to 0.19]	0.0 [0.0 to 0.0]
*Random Forest* (33)	Climate	~1,000 m	0.7056 [0.69 to 0.76]	0.0219 [0.02 to 0.025]	0.5714 [0.40 to 0.74]	0.4129 [0.09 to 0.69]	0.0073 [0.0 to 0.012]	0.0137 [0.01 to 0.02]	0.5113 [0.44 to 0.69]	0.2234 [0.20 to 0.28]	0.0288 [0.0 to 0.06]
*Frequency*	N/A	N/A	0.5 [0.5 to 0.5]	0.0045 [0.0 to 0.005]	0.0801 [0.01 to 0.09]	0.0 [0.0 to 0.0]	0.0 [0.0 to 0.0]	0.0 [0.0 to 0.0]	0.103 [0.07 to 0.12]	0.3008 [0.22 to 0.31]	0.0 [0.0 to 0.0]

Median [IQR] are reported for each accuracy metric across ten latitudinal cross-validation blocks (*SI Appendix*, SM 1.3.2 and Fig. S4*B*). For accuracy results per-image, see *SI Appendix*, Table S10. Res. = Resolution; *MLP* = multilayer perceptron; *spp* = per-species; img = per-image; AUC_ROC_ = area under receiver operating curve; AUC_PRC_ = area under precision–recall curve; Prec = precision; mAP = mean average precision, Pres. Acc. = Presence Accuracy.

**Fig. 2.**
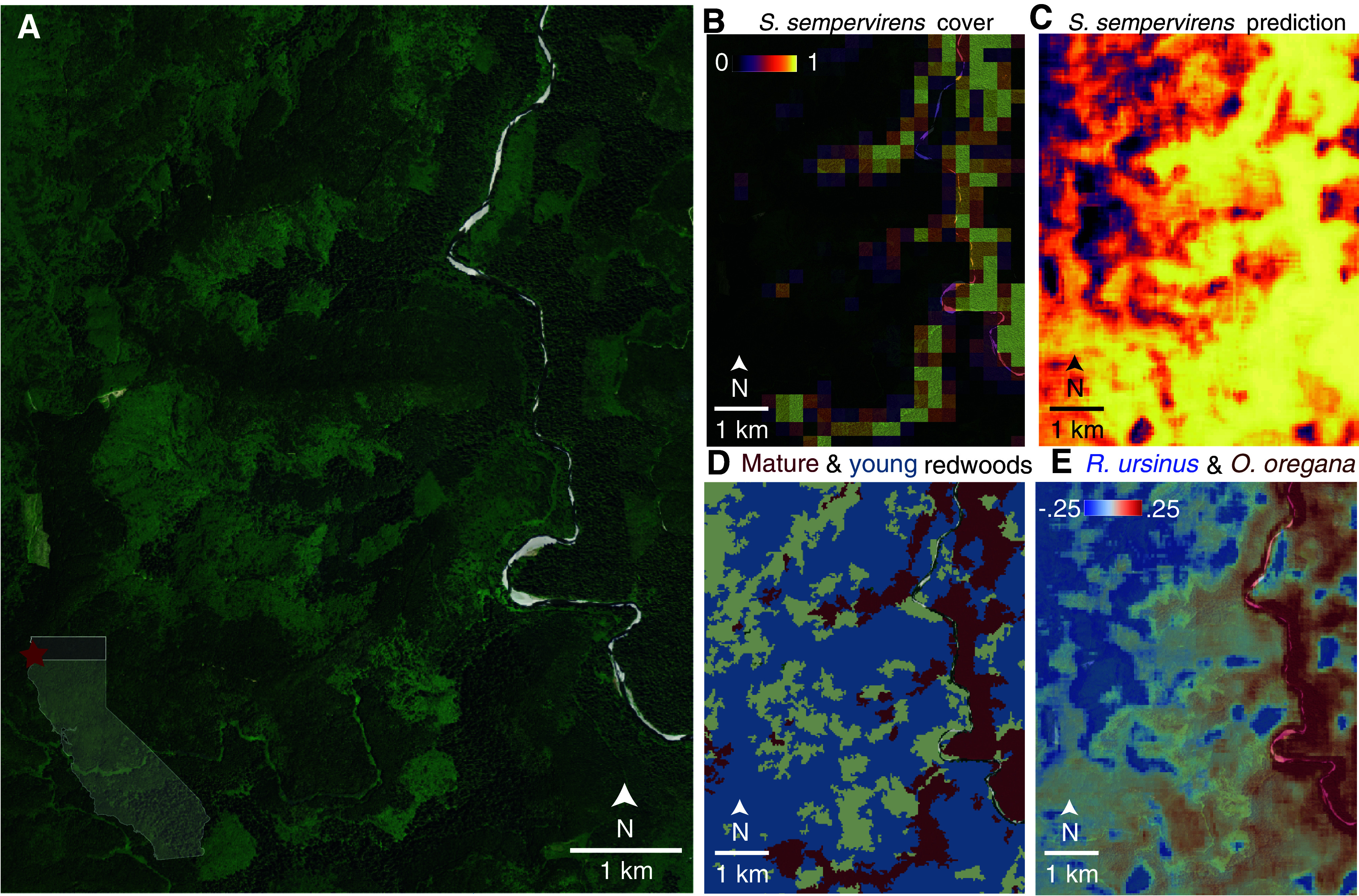
*Deepbiosphere* predictions of field-validated coastal redwood forest species. (*A*) NAIP aerial imagery (16) of Tall Trees redwood grove (*Sequoia sempervirens*) in California’s Redwoods National and State Parks. The region contains some of the last old-growth redwood remnants in the world, visible as the dark green line bordering the right-hand side of Redwood Creek. (*B*) Human annotations of redwood forest cover at 256 m resolution, based on examples of other old-growth redwood groves (*SI Appendix*, Fig. S9). Annotators can correctly distinguish mature groves (*SI Appendix*, Fig. S10*B*). (*C*) *Deepbiosphere* predicted presence of *S. sempervirens* at 50 m resolution. *Deepbiosphere* correctly detects both mature and young regrowth groves (*SI Appendix*, Fig. S10*A*). (*D*) Official NPS vegetation map (18) highlighting mature redwood (dark red) and young redwood regrowth (blue) vegetation classes. (*E*) *Deepbiosphere’s* difference in predicted presence of two understory species: *Oxalis oregana* which has a preference for mature redwood stands, and *Rubus ursinus* which has a preference for secondary-growth redwood forest (18, 39). Differences were calculated by subtracting the predicted presence of *Oxalis oregana* from the predicted presence of *Rubus ursinus* per-map pixel.

The ultimate goal of building predictive SDMs is to study where species are and what environmental features or human activities have shaped their ranges. Doing so at high spatial resolution may enable the detection of certain signatures of anthropogenic impacts on plant biodiversity, such as the lasting effects of deforestation. To test this hypothesis, we generated high-resolution species maps using *Deepbiosphere* by iteratively predicting the presence of all ~2,000 species across NAIP tiles, yielding species presence prediction maps at up to a few meters resolution per-pixel (*SI Appendix*, SM 4.2 and Fig. S8). We focused on a region emblematic of anthropogenic biodiversity: the redwood forests of coastal California (*Sequoia sempervirens*; 2,349 observations in the dataset). Redwood forests are highly heterogeneous due to heavy logging in the mid-20th century which decimated 95% of the old-growth forest (38). The scars of this deforestation are easy to recognize from aerial imagery, including around the iconic old-growth Tall Trees grove (Fig. 2*A* and *SI Appendix*, Table S11), which has been fully mapped by the National Park Service (NPS) to the vegetation association level, including by forest age (18) (Fig. 2*D* and *SI Appendix*, SM 4.4). These mature groves were manually annotated by humans (Fig. 2*B* and *SI Appendix*, SM 4.3 and Fig. S9) with high accuracy (mature redwood pixels true positive rate: 93.5%, Fig. 2*D* and *SI Appendix*, Fig. S10*B*), but human annotators failed to detect additional postclear cutting secondary-growth redwood forest in the area (young redwood pixels true positive rate: 2.6%, *SI Appendix*, Fig. S10*C*), ultimately generating low accuracy maps when considering the full redwood forest extent (all redwood pixels binary classification accuracy: 37.9%, *SI Appendix*, Fig. S10*A*).

In contrast to these human-derived maps, *Deepbiosphere’s* redwood presence map indicated a much broader distribution of redwoods (Fig. 2*C*) and correctly demarcates both mature redwood groves (mature redwood pixels true positive rate: 100.0%, Fig. 2*D* and *SI Appendix*, Fig. S10*B*) and young secondary regrowth redwood forest (young redwood pixels true positive rate: 89.7%, *SI Appendix*, Fig. S10*C*), generating an accurate map of redwood forest extent (all redwood pixels binary classification accuracy: 81.4%, *SI Appendix*, Fig. S10*A*). This young redwood forest is difficult to detect not just for humans but also climate-only and remote sensing-only SDMs (*SI Appendix*, Fig. S11), which in general predict redwoods as absent across the study area (*Maxent* all redwood pixels true positive rate: 0.0%, *Inception V3* all redwood pixels true positive rate: 0.0%, *SI Appendix*, Fig. S10*A*) and have poor predictability across the broader region (*SI Appendix*, Table S12). While *Deepbiosphere* can accurately predict species probabilities from remote sensing imagery (Tables 1 and 2)—including across fragmented and heterogeneous landscapes and at fine spatial scales (Fig. 2*C*)—currently, *Deepbiosphere* is still a correlative approach and not a truly process-based method (40). Since fragment size, distance from fragment edges, distance between similar patches, and landscape context are all known to affect species distributions (41–44), a powerful way to better map and understand the human-altered distributions of plant species across space and time would be to combine *Deepbiosphere’s* species presence maps with models that explicitly account for these anthropogenic drivers (40).

Along with redwoods themselves, mid-20th century clear-cutting also dramatically altered understory species composition (39), but mapping these species from remote sensing imagery can be challenging when individuals are not directly detectable (45). While these understory species may not be directly visible from above, they often show preferences for certain habitats and form visually distinguishable communities (18), preferences which *Deepbiosphere* could potentially exploit to map these understory species. To explore this hypothesis, from six field-validated focal understory species (*SI Appendix*, SM 4.4 and Figs. S12 and S13), we focused on two understory species common to redwood forests: *Oxalis oregana* (redwood sorrel; 1,063 observations in the dataset) and *Rubus ursinus* (California blackberry, 458 observations in the dataset), which *Deepbiosphere*-generated maps suggest occupy different types of redwood forest (Fig. 2*E*). *Deepbiosphere* predicted *Oxalis oregana* with the highest probability mainly in mature redwood groves (median [IQR] *Deepbiosphere* predicted presence *O. oregana*: mature redwood map pixels = 0.996 [1.0 to 0.99], young redwood map pixels = 0.944 [0.98 to 0.85], *t* test *P*-value < 2 × 10^−16^, *SI Appendix*, Fig. S12*B*), matching field-validated associations with cool and moist old-growth redwood understories (18, 39) (median [IQR] field-validated *O.* *oregana* constancy measurements: mature redwood associations = 0.96 [0.98 to 0.96], young growth associations = 0.49 [0.62 to 0.35], *t* test *P*-value = 0.057, *SI Appendix*, Fig. S13*B*). Meanwhile, *Deepbiosphere* predicted *Rubus ursinus* with high probability mainly in young redwood regrowth (median [IQR] *Deepbiosphere* predicted presence *R. ursinus*: mature redwood map pixels = 0.864 [0.92 to 0.76], young redwood map pixels = 0.957 [0.97 to 0.93], *t* test *P*-value < 2 × 10^−16^, *SI Appendix*, Fig. S12*E*), reflecting a preference for semishaded young redwood understory also validated by field measurements (18, 39) (median [IQR] field-validated *R. ursinus* constancy measurements: mature redwood associations = 0.27 [0.28 to 0.14], young growth associations = 0.735 [0.85 to 0.65], *t* test *P*-value = 0.057, *SI Appendix*, Fig. S13*C*).

These associations are further supported by analyses of other well-known understory species associated with either mature and/or regrowth redwood forests (18, 39) (see case studies of *Struthiopteris spicant, Viola sempervirens, Polystichum munitum,* and *Vaccinium ovatum,* *SI Appendix*, Figs. S12 and S13). In contrast, climate-based SDM species presence maps were qualitatively too low-resolution to capture these deforestation-induced differences (*SI Appendix*, Fig. S11*C*) and were quantitatively less accurate detecting known species occurrences from the region (*SI Appendix*, Table S12). *Deepbiosphere’s* ability to accurately map the distribution of both canopy trees and small herbaceous plants also extends to other habitats, including Southern California’s mediterranean ecosystems where *Deepbiosphere’s* species predictions better matched previously mapped vegetation distributions (19, 46) and better detected known presences from the region compared to climate-based models (see case studies of *Quercus lobata, Q. berberidifolia, Ceanothus cuneatus, Bromus diandrus, Arctostaphylos glandulosa, Adenostoma fasciculatum,* *SI Appendix*, SM 4.5, Figs. S14–S16, and Table S13). Furthermore, *Deepbiosphere* can generate regional maps of species distributions with high accuracy for both well-predicted species (*Deepbiosphere* average AUC_ROC_ = 0.972, *Maxent* average AUC_ROC_ = 0.917, average *Deepbiosphere* AUC_ROC_ improvement = 5.53%, *SI Appendix*, Fig. S22) and random species (*Deepbiosphere* average AUC_ROC_ = 0.941, *Maxent* average AUC_ROC_ = 0.909, average *Deepbiosphere* AUC_ROC_ improvement = 3.15%, *SI Appendix*, Fig. S23), as quantified using an independently derived set of species occurrence records from Calflora (47) (*SI Appendix*, SM 4.1). These improvements likely stem from the rich habitat information present in remote sensing imagery that *Deepbiosphere* can leverage and are especially pronounced for disturbance-related, open-ground, coastal, or wetland species like *Lupinus arboreus* (AUC_ROC_ improvement of 19.6%, *SI Appendix*, Fig. S22)*, Coreopsis gigantea* (AUC_ROC_ improvement of 10.4%, *SI Appendix*, Fig. S22)*, Malacothrix saxatilis* (AUC_ROC_ improvement of 17.4%, *SI Appendix*, Fig. S22)*, and Juncus acutus* (AUC_ROC_ improvement of 9.7%, *SI Appendix*, Fig. S22) whose unique habitat characteristics are readily visible from remote sensing imagery. *Deepbiosphere’s* remote sensing-based approach especially enables the creation of range maps at fine spatial scales (see *SI Appendix*, Figs. S24–S28 for additional high-resolution case studies). Together, these results demonstrate that deep learning can map both large tree species (48) and small herbaceous plants from high-resolution remote sensing imagery and, from these data, detect the lasting effects of deforestation on entire plant communities decades later.

**Fig. 3.**
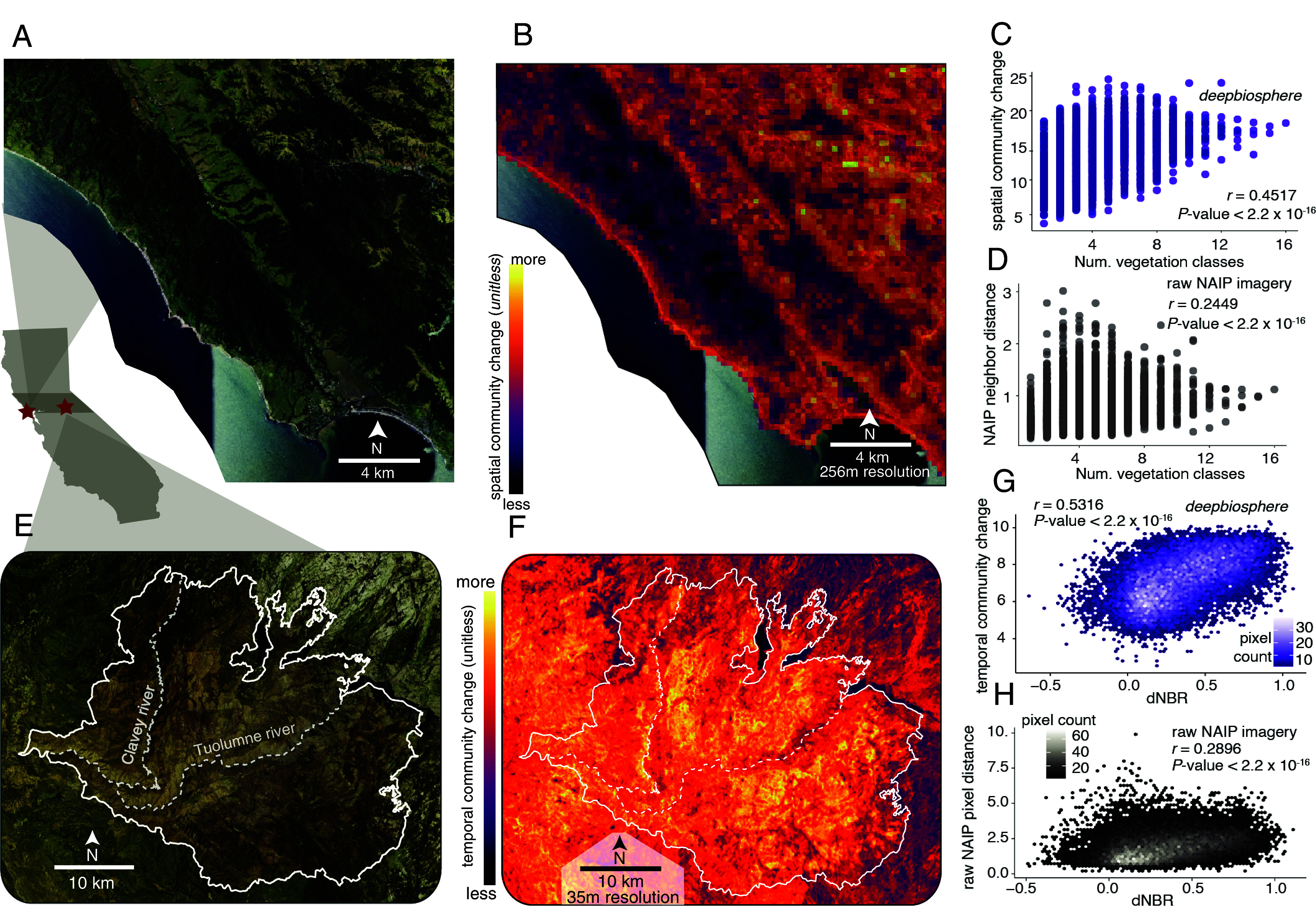
Detection of high-resolution spatial and temporal community change using *Deepbiosphere*. (*A*) NAIP aerial imagery of northern Marin county (16). Marin county has substantial habitat fragmentation and sits between two major ecoregions, the coast range and oak chaparral. (*B*) *Deepbiosphere’s spatial community change* calculated using an edge-detection algorithm applied to the predicted presence of all 2,221 plant species (*SI Appendix*, SM 5.1 and Fig. S19). (*C*) Comparison of the number of unique fine-scale vegetation types (20) present in each 256 × 256 m plot and *Deepbiosphere’s spatial community change* predictions. (*D*) Comparison of the number of unique fine-scale vegetation types (20) present at each 256 × 256 m plot and an edge-detection algorithm run on raw NAIP RGB-I images. (*E*) 2014 NAIP aerial imagery (16) of Sierra foothills after severe Rim Fire of 2013 (fire boundary in white). (*F*) *Deepbiosphere’s temporal community change* metric, calculated using the Euclidean distance between predicted species presence in 2012 and 2014 (*SI Appendix*, SM 5.2 and Fig S21). (*G*) Comparison of an empirical burn severity metric—difference in normalized burn ratio (dNBR, *SI Appendix*, Fig. S20*C*) (21)—with *Deepbiosphere’s* temporal community change from f. (*H*) Comparison of dNBR with the Euclidean distance calculated between raw NAIP RGBI-I imagery from 2012 and 2014 (16) (*SI Appendix*, Fig. S20*E*).

One major effect of deforestation—and land use or environmental change more broadly—is the increasing fragmentation of native habitat (2, 7). While fragmentation can depress plant genetic diversity (49), natural ecosystem transitions (called ecotones) often instead exhibit increased biodiversity (50). Marin County is a prime example of both kinds of ecosystem edges, containing both fragmented native vegetation broken up by agricultural areas and urban districts (Fig. 3*A*), but also many ecotones, as it sits on the boundary of the coast range and valley chaparral ecosystems (20) (*SI Appendix*, Figs. S17*C* and S18). We wondered whether *Deepbiosphere* could be used to automatically delineate these important areas of spatial biodiversity change. To do so, we adapted an edge-detection algorithm from image processing, and for each 256 × 256 m aerial image in the region, we generated presence probabilities for all 2,221 species from *Deepbiosphere*. We then calculated the average Euclidean distance between these probabilities and the probabilities from the eight neighboring images, where a larger Euclidean distance means higher turnover (*SI Appendix*, SM 5.1 and Fig. S19). Mapping this species turnover metric that we call *spatial community change* across north Marin county captured both mountain-to-valley and developed-to-undeveloped ecotone edges (Fig. 3*B*). Quantifying these results, *Deepbiosphere’s* estimated *spatial community change* strongly correlated with the number of unique vegetation classes in the official Marin County fine-scale vegetation map (20) (Pearson’s *r* = 0.45, *P*-value < 2.2 × 10^−16^, Fig. 3*C* and *SI Appendix*, Fig. S17*C*), more strongly than the raw underlying NAIP imagery (Pearson’s *r* = 0.25, *P*-value < 2.2 × 10^−16^, Fig. 3*D* and *SI Appendix*, S17*D*), and the density of *iNaturalist* observations (Pearson’s *r* = 0.08, *P*-value = 0.02, *SI Appendix*, Fig. S17 *E* and *F*). These results suggest that *Deepbiosphere’s* aggregated species predictions can be used to automate detection of important spatial patterns of anthropogenic biodiversity at high-resolution.

Monitoring plant biodiversity in the Anthropocene ultimately will require detecting rapid changes in plant communities caused by forest clear-cutting, flooding, and wildfires. High-resolution remote sensing data collected at weekly-to-yearly timescales can capture these events (21, 51, 52), and thus remote sensing-based SDMs should capture their effects on plant communities. Last, we showcased *Deepbiosphere’s* ability to detect fire-induced community-level change across time precipitated by the 2013 Rim wildfire in the western California Sierra foothills (Fig. 3*E*). The Rim Fire’s burn scar is clearly visible from NAIP imagery taken both before and after the fire (2014 vs. 2012, *SI Appendix*, Fig. S20 *A* and *B*). From these two images, we generated presence prediction maps for all 2,221 species at 35 m resolution from *Deepbiosphere* and calculated the Euclidean distance between these predictions per-map pixel to generate an estimate of *temporal community change*, with a higher Euclidean distance indicating a more pronounced change in time (*SI Appendix*, SM 5.2 and Fig. S21). *Deepbiosphere’s* predicted *temporal community change* was higher inside the fire’s boundary than outside (unpaired student’s *t* test, *P*-value < 2.2 × 10^−16^, Fig. 3*F* and *SI Appendix*, Fig. S20*F*), implying that the fire substantially changed the community composition of the burned habitats. To further quantify these results, we compared the predicted *temporal community change* to a field-calibrated, NDVI-like metric used to map burn severity from hyperspectral imagery called the difference in Normalized Burn Ratio (dNBR) (21) (*SI Appendix*, SM 5.2 and Fig. S20*C*). *Deepbiosphere’s* predicted *temporal community change* significantly correlated with these independently measured dNBR severity estimations (Pearson’s *r* = 0.53, *P*-value < 2.2 × 10^−16^, Fig. 3*G*), far exceeding the correlation of dNBR with the Euclidean distance between the original NAIP RGB + Infrared images from 2012 and 2014 (Pearson’s *r* = 0.29, *P*-value < 2.2 × 10^−16^, Fig. 3*H* and *SI Appendix*, Fig. S20*E*). Compared to traditional highly specialized hyperspectral approaches used in fire ecology, *Deepbiosphere* can detect fire-induced community change from simple RGB-I imagery, solidifying the potential of deep learning-based SDMs trained on ubiquitous remote sensing imagery to detect rapid anthropogenic community changes cost-effectively and at scale.

To achieve the United Nations’ Global Biodiversity Framework, a paradigm shift in species distribution modeling for global biodiversity monitoring is needed (53). Prioritizing species-rich sensitive areas or fragmented habitats will require maps of thousands to millions of species at high spatial resolution. Tracking progress toward restoring 30% of the world’s degraded lands will involve synthesizing large amounts of data of different modalities. Detecting and attributing environmental disasters and illegal impacts on ecosystems calls for temporally explicit approaches. Toward these goals, here, we showcased how deep learning models can perform complex biodiversity monitoring tasks, from fine mapping of a fragmented keystone species and its community to detecting rapid shifts in species presence after a massive fire. While *Deepbiosphere’s* accuracy is overall higher than other SDMs and can generate species range maps at high-resolution, many species with few observations are still hard to account for. Nevertheless, expanding *Deepbiosphere* beyond plants to predict species across of the tree of life should help improve these cases by providing more signal of the complex interspecific relationships that weave together and define ecological communities. Ultimately, we envision a paradigm shift toward open-source foundation models (54) that are continuously trained and improved with new remote sensing data, citizen science observations, and data modalities as they become available. Achieving this from public airborne or satellite imagery and growing citizen science observations will make biodiversity monitoring more accessible, thus advancing local and global nature conservation goals.

## Methods

### Species Observations.

We collected observations from kingdom *Plantae* using *GBIF.org* from the years 2015 to 2022 (22). Only records observed by humans with a coordinate uncertainty radius of less than or equal to 120 m with no flagged geospatial issues were taken from within the state of California (*SI Appendix*, SM 1.1). We downloaded a total of 912,380 observations of 5,193 unique plant species and further filtered the dataset to only include vascular plants, remove duplicate observations of the same species within a 150 m radius, remove species that contain all observations located within a 256 m radius, remove observations that were not geographically located within the Global Administrative Area boundary of California, and remove observations that were not located within both the climatic and remote sensing imagery rasters (see *SI Appendix*, SM 1.1 for details, *SI Appendix*, Table S1 for more dataset details). To increase the density of observations and allow for multiple species within a single image, we used neighbor imputation to add any other species observed within an overlapping 256 m radius to a given observation (*SI Appendix*, SM 1.2 and Fig. S2). We finally removed any species that had fewer than 500 total observations in the dataset after neighbor imputation, leaving us with a total of 652,027 observations of 2,221 unique plant species (23, 24).

### Remote Sensing Data.

To link species observations with images, we utilized aerial imagery from the NAIP (16) which we downloaded for the entire state of California from 2012 and 2014 using Microsoft Azure’s NAIP data blob disk image on its West Europe and Eastern US servers (*SI Appendix*, SM 1.4). For training the CNN models, we specifically used the NAIP data from 2012 at 1-m resolution to generate 256 × 256 pixel images, where 1 pixel corresponds to a 1 × 1 m resolution (23). We used all available bands for training, specifically the RGB and infrared color bands (*SI Appendix*, Table S1). The 256 × 256 pixel images were extracted so that the geographic coordinates of the corresponding species observation mapped to the center of the image (*SI Appendix*, Fig. S2).

### Climate Variables.

We used the 19 bioclimatic variables available from WorldClim Version 2 (31) at 30 arc-second (approximately 1 km) per-pixel resolution. Variables were downloaded directly from the WorldClim Version 2 repository (*SI Appendix*, SM 1.5). Before fitting any model, all bioclimatic variables were normalized per-variable to mean 0 and SD of 1 using the entire raster clipped to the outline of California.

### Train/Test Split and Cross-Validation.

In order to properly validate and compare models, we split the dataset into multiple partitions. The first partition, which was used for hyperparameter tuning and loss comparison, was generated by randomly selecting observations uniformly from across the state (23) (*SI Appendix*, SM 1.3.1 and Fig. S4*A*) which we refer to as the *uniform partition* and use the notation *modelname_unif_* to refer to models trained using this partition of the dataset. For this train/test partition, we chose points uniformly across the state to maximize the number of unique ecosystems models would be evaluated on. To ensure the independence of training and testing set data due to spatial autocorrelation, we added all overlapping observations to the test set to guarantee that none of the remote sensing images and observations in the test set were present in the training set. To further ensure that there was no data leakage between the test and train set, only observations which were more than 1,300 m away from any other nonoverlapping observation were included. We chose an exclusion radius of 1,300 m because the climate variable raster pixels converted from arc-seconds to meters can have a diameter of up to 1,200 m, so any test set observation within that distance to any observation in the train set would have an identical input value as some observations used during fitting. Ultimately 12,277 observations (1.88% of the dataset) were set aside for testing in this split.

In order to provide cross-validation of the uniform train-test split and to test the extrapolation ability of all models, we also conducted a latitudinal ten-fold spatial holdout block validation by partitioning California into ten one-degree latitudinal bands (23) (*SI Appendix*, SM 1.3.2 and Fig. S4*B*) which we refer to as the *spatial partition*, using the notation *modelname_k_* to refer to models trained using points from the k-th spatial block (*SI Appendix*, Fig. S4*B*). Training observations within 1,300 m of the test band were removed to prevent data leakage as discussed above. For SDMs fitted with pseudoabsence points, all pseudoabsence points within the test bands were removed to ensure a fair comparison to presence-only models. Ultimately, the percentage of test points per-spatial block ranged from 1.40 to 25.35% of the entire dataset.

### Deep CNNs for SDMs.

We chose to use the medium-sized *TResNet* architecture, a CNN-based residual neural network (27) which is optimized for fast inference on Graphics Processing Units (GPUs) and optimized for multilabel image classification. We modified the *TResNet* architecture to have four input channels in order to support the RGB and infrared NAIP imagery and added three fully connected output layers corresponding to three taxonomic ranks (family, genus, and species) that confer some phylogenetic signal during training (28) (*SI Appendix*, SM 3.2.1 and Table S2). All *TresNet*-based CNNs are trained to predict each of the 2,221 plant species simultaneously, giving a probability of presence from 0 to 1 for all species. Along with this standard version of the *TResNet* architecture trained using only the NAIP aerial imagery, we also created our own custom CNN model which combines a *TResNet* head trained using NAIP imagery with an MLP multilayer perceptron head trained using climate inputs (*SI Appendix*, SM 3.2.3) which we refer to as the *Deepbiosphere* model (Fig. 1*B* and *SI Appendix*, Table S3). Weights were initialized following best practices laid out in the original *TResNet* paper, using Kaiming He-style for CNN layers and zeroed out BatchNorm and residual connections. For all analyses, *TresNet*-based CNN outputs were converted to independent probabilities using the sigmoid transformation.

We compared the performance of the *TResNet* architecture trained on a variety of standard loss functions (*SI Appendix*, SM 3.2.2). The loss function compares how well CNN outputs align with a training set of observations and thus determines how well the model fits the data and learns from it. While we report results from a variety of common loss functions for fair comparison to previous work, the final results use a new loss function we called sampling-aware BCE that overcomes limitations of common functions like cross-entropy loss which is best suited for single-label images, BCE which is best suited for multilabel images where the absence of labels are informative, or the recent asymmetric focal loss which is best suited for multilabel images where many mislabels may occur, by weighing the contribution of the few species present in any given image as much as the contribution of the many species that are absent.

For comparison to previous work using CNNs to rank species presence from remote sensing imagery, we trained an *Inception V3* architecture (34) (*Inception V3,* *SI Appendix*, SM 3.2.4 and Table S4) with softmax cross-entropy loss using the official architecture implementation and initial weights from pytorch and using both the standard and auxiliary loss during training. We utilize the standard dropout rate of 0.5 and a standard learning rate of 0.01, different but comparable hyperparameters to those used in previous work. For all analyses, the *Inception V3* outputs were converted to a probability density function using the softmax transformation. While the *Inception V3* model is trained jointly across all species like *Deepbiosphere*, the cross-entropy loss forces the *Inception V3* CNN to fit a probability density function across species and as such the predicted probabilities per-species are not independent.

All CNNs were trained with standard minibatch stochastic gradient descent for 13 epochs using the Adam optimizer. The epoch of evaluation was determined using early stopping calculated from the per-species average AUC_ROC_ on the uniform test set split (see *SI Appendix*, SM 2.2 for metric details). Learning rates were tested using a stepwise sweep ranging from 5 × 10^−6^ to 1 × 10^−1^ in increments of 0.5 and batch sizes were chosen depending on model size relative to the GPU size used for training. Batch size, learning rate, memory usage, and GPU architecture used for training are reported for each CNN in *SI Appendix*, Tables S2–S10.

### SDM Based on Climate Rasters and Other Baselines.

We use the popular *dismo* R package for species distribution modeling and compared against two popular SDM approaches: *Maxent* and downsampled single stacked random forest (RF). We chose these two models as they consistently had the best performance across dozens of models and hundreds of species in a large benchmarking experiment (33) (*SI Appendix*, SM 3.3.1 and 3.3.2). We removed all but one bioclim variable with a Pearson correlation coefficient higher than 0.8, leaving ten variables in total for modeling including Mean Diurnal Range, Max Temperature of Warmest Month, Minimum Temperature of Coldest Month, Annual Precipitation, Precipitation of Wettest Month, Precipitation of Driest Month, Precipitation Seasonality, Precipitation of Wettest Quarter, Precipitation of Warmest Quarter, and Precipitation of Coldest Quarter. For each species, we generated 50,000 background samples using a circular overlay across all points in the training dataset where the radius of each circle is the median distance between said species’ observations. We used the same number of presence and background points for both the RF and *Maxent* models and we used the “nothreshold” option for *Maxent* and 1,000 trees with equal bootstrapping of positive and negative samples with replacement, with all other options set using *dismo* default. For a few species, the fitting process failed for *Maxent* and/or RF. For these species, in downstream accuracy analyses, we impute an accuracy of 0 for all metrics.

For completeness, we also trained a fully connected, feed-forward MLP on all 19 bioclim variables to predict all 2,221 species simultaneously as a climate-only deep learning baseline (*SI Appendix*, SM 3.3.3). The architecture consists of two fully connected layers with 1,000 neurons each, followed by a dropout layer with a 0.25 dropout rate, then by two layers with 2,000 neurons each (32), before predicting species, genus, and family (*SI Appendix*, Table S5). The random baseline was calculated by drawing random values from a standard normal distribution ten times and averaging the accuracy metrics across these ten trials (*SI Appendix*, SM 3.3.4). The frequency baseline involved calculating the frequency of observations per-species on the training set, rescaling the probabilities to 0.001 to 1.0 and imputing these frequencies as the predicted probabilities at each test set example (*SI Appendix*, SM 3.3.4).

### Accuracy Metrics.

We utilized a wide variety of accuracy metrics from across a variety of relevant disciplines, from computer vision to species distribution modeling. For the full list of reported accuracy metrics and their explicit mathematical definitions, see *SI Appendix*, SM 2. The reported accuracy metrics can be classified into three broad categories.

The first category of accuracy metrics—binary classification metrics, *SI Appendix*, SM 2.1—captures an SDM’s ability to correctly predict the presence or absence of a species given a probability of presence threshold. We report precision, recall, and F1 score both per-species and per-image, along with presence accuracy. For all reported binary classification metrics in the main text, figures, tables, and supplemental (precision, recall, *F1*, presence accuracy), we use a standard 0.5 threshold. This is not only a common threshold in species distribution modeling, but it is a standard threshold used by the computer vision community, as when using a sigmoid-based loss function, values above 0.5 map to positive real-valued numbers and values below 0.5 map to negative real-valued numbers. However, the requirement to choose a threshold for classification makes binary classification metrics less desirable as accuracy metrics for species distribution modeling, and other metrics are thus generally favored.

Unlike binary classification metrics, the next category of accuracy metrics—discrimination metrics, *SI Appendix*, SM 2.2—calculates an SDM’s performance across a wide range of presence thresholds and describe the relationship between threshold change and performance change. Discrimination metrics essentially integrate accuracy across a gradient of presence thresholds, negating the need to pick a threshold value like binary classification metrics, and is a deciding factor in why discrimination metrics like AUC_ROC_ are very commonly used metrics for selecting SDMs. For this reason, we primarily focus on AUC_ROC_ for comparing model performance across the range of case studies in this work. Reported discrimination metrics in this work include AUC_ROC_ and area under the precision–recall curve (AUC_PRC_), averaged across species (*_spp_*), and a calibrated version of both metrics (calibrated AUC_ROC_ and calibrated AUC_PRC_).

The third and final accuracy category—ranking metrics, *SI Appendix*, SM 2.3—focus solely on how high a given species is ranked by probability of presence compared to other species in the same image or observation and are common in machine learning and computer vision research. In this work, we report Top-1, Top-5, Top-30, and Top-100 accuracy across observations and species, plus mean average precision.

### Case Studies of Species and Ecosystems.

For both case studies, locations were chosen using expert knowledge of the respective species ranges and known occurrences from Calflora (47). Three nonexpert human annotators annotated *Sequoia sempervirens* cover and two annotated *Quercus lobata* cover. To calibrate annotators to the task, each annotator received three NAIP images from 2012 and an assigned cover classification using known species occurrences pulled from Calflora (47) (*SI Appendix*, SM 4.3 and Figs. S9, S14*C*, and Table S11). Annotations took between 30 min to 2 h per-case study (depending on the efficiency and familiarity of the annotators with the task) and final cover scores were calculated by averaging annotations per-pixel across annotators.

High-resolution species predictions at 50 m resolution were generated from the CNNs by convolving the 256 × 256 pixel prediction window with a stride of 50 (*SI Appendix*, SM 4.2 and Fig. S8). It is important to note that the versions of *Deepbiosphere, Maxent, RF*, and *MLP* used in these case studies were trained without observations or pseudoabsences from the respective spatial cross-validation band where the case study was located (see darkened band inside California inset in Fig. 2 and *SI Appendix*, Fig. S14). Thus, these models did not see any example images or climate variables from the respective regions at train time, with the nearest training examples located between 9 to 20 km away from the case studies (*SI Appendix*, Fig. S4*B*). Conversely, the *Inception V3* baseline was trained using multiple observations from within the parks (specifically using the uniform data split, (*SI Appendix*, Figs. S4 *A* and S11*B*).

For the redwoods case study, the 2017 NPS generalized alliance-level map was used for vegetation comparison (18), with the class “mature redwoods” mapping to the *Sequoia sempervirens* mature forest alliance, the class “young redwoods” mapping to the *Sequoia sempervirens*-(other) YG alliance, and the class “other vegetation” mapping to all other alliance-level classes present in the study area (*SI Appendix*, SM 4.4). Per-pixel labels were determined based on which alliance had the largest area overlap with the pixel’s extent.

For the oaks case study, the United States Department of Agriculture (USDA) Forest Service’s 2018 map of existing vegetation in Region 5's South Coast Ecological Province was used for comparison (19), specifically the type 1 regional dominance map with species crosswalked to vegetation type using the vegetation class descriptions from Region 5's CALVEG Zone 7 (46) (*SI Appendix*, SM 4.5). For the per-species analysis, the species to CALVEG mappings are as follows: *Ceanothus cuneatus*: CC, CQ, and EX; *Quercus lobata*: QL; *Bromus diandrus*: HG; *Quercus berberidifolia*: CQ; *Arctostaphylos glandulosa*: CQ and SD, *Adenostoma fasciculatum*: QA, CC, CQ, SS, and EX. For each species, pixels were marked as “inside” if said pixel intersected with at least one of the associated CALVEG classes for that species.

For the additional full-state species range map examples, range maps at 150 m resolution for *Deepbiosphere* and ~1 km resolution for *Maxent* were generated for the best-predicted species by AUC_ROC_ by *Deepbiosphere* and *Maxent* for the five L2 ecoregions of California using a minimum 0.98 accuracy threshold and 10 test set observations to choose species to display, or species were randomly selected using numpy’s random.choice function and a random seed of 1 (*SI Appendix*, SM 4.1 and Figs. S22, S23). For the quantitative accuracy assessment, occurrence records were derived from Calflora (47), specifically for each species using all observations uploaded directly from Calflora, the Consortium of California Herbaria, and the Consortium of North American Bryophyte Herbaria (excluding just records derived from *iNaturalist*). All subsequent occurrences with location information from inside California were included, including obscured records, all varieties, and subspecies. Absence locations for calculating AUC_ROC_ were derived from the location of all other Calflora occurrences for the selected species not predominantly found in the species’ ecoregion (e.g., excluding observations for all other species predominant to the Warm Deserts L2 ecoregion for *Bahiopsis parishii*). For each L2 ecoregion, one species was chosen for a high-resolution case study zoom-in at ~1, 0.1, and 0.001 degrees resolution (*SI Appendix*, Figs. S24–S28).

### Spatial Community Change Metric.

For calculating spatial community change using *Deepbiosphere*, we designed an edge detection algorithm inspired by edge-detection filters from the field of computer vision. Specifically, the averaged one-neighbor Euclidean norm was calculated per-pixel to generate a map of averaged similarity to neighbor pixels using standard 256 m resolution *Deepbiosphere* predictions (*SI Appendix*, SM 5.1). This algorithm essentially measures the average distance from a given pixel’s species prediction to all its nearest neighbors’ predictions, summarizing how similar or different a given pixel’s predicted species list is from nearby areas (see *SI Appendix*, Fig. S19 for visual walkthrough). To validate *Deepbiosphere’s* spatial community change predictions, we utilized the 2018 Marin fine-scale vegetation map (20) to calculate the number of vegetation classes intersecting each pixel. Pearson’s *r* between the number of intersecting vegetation classes and spatial community change was calculated using the spatially corrected modified *t* test from SpatialPack, using the centroid of each pixel as the coordinates per-sample. A similar comparison to the number of intersecting vegetation classes was performed using the averaged one-neighbor Euclidean norm between the normalized raw NAIP pixel values per-band, upsampled to 256 m resolution.

### Temporal Community Change Metric.

For calculating temporal community change using *Deepbiosphere,* we used the per-pixel Euclidean distance between *Deepbiosphere’s* predicted species probabilities made at two different timepoints (*SI Appendix*, SM 5.2 and Fig. S21). This change metric essentially measures the magnitude of per-species change (including both increases and decreases) aggregated between the two timepoints. To validate *Deepbiosphere’s* temporal community change predictions for the Rim Fire, we compared an independently generated map of dNBR (21) to *Deepbiosphere*-generated temporal community change predictions made using 2012 and 2014 NAIP imagery. Pearson’s *r* between temporal community change and nDBR was calculated using the spatially corrected modified *t* test from SpatialPack, using the centroid of each pixel as the sample coordinates. For this comparison, we used 256 m, nonstrided species predictions from *Deepbiosphere* and dNBR upsampled to 256 m resolution to minimize spatial autocorrelation and ensure the memory-intensive spatially corrected modified *t* test could run in sufficient time. A similar comparison to upsampled dNBR was performed using the Euclidean distance between the normalized raw NAIP pixel values per-band upsampled to 256 m resolution.

## Supplementary Material

Appendix 01 (PDF)

## Data Availability

Training data are publicly available through GBIF.org (24) and NAIP (16), and original occurrence records can be found at ref. 22. Code to build paired image-species datasets and to train *Deepbiosphere* are available at github.com/moiexpositoalonsolab/deepbiosphere (23). Documentation of use is provided within the code. Additional data were used from the vegetation mapping and classification project for Redwood National and State Parks, California (2017) conducted by ref. 18; the Marin County Fine Scale Vegetation Map (2021) created by ref. 20; old-growth and unmanaged second-growth riparian forest plots at Redwood National Park, USA by ref. 39; climate data from ref. 31; remote sensing data before and after the California Rim and King forest fires (2010 to 2015) by ref. 21; existing vegetation data for the USDA Forest Service Region 5— Zone 7, South Coast (2018) by ref. 19; additional California plant observations by ref. 47; and the U.S. General Soil Map (STATSGO2) by ref. 55.
