# Supplementary material for: Deep learning models map rapid plant species changes from citizen science and remote sensing data
Source: Proc Natl Acad Sci U S A. 2024 Sep 5;121(37):e2318296121. doi: 10.1073/pnas.2318296121 (PMC11406280; doi:10.1073/pnas.2318296121)
Supplement: Supplementary file 1 — Appendix 01 (PDF) [file pnas.2318296121.sapp.pdf]

Supplementary Materials for:

Deep learning models map rapid plant species changes from citizen science and remote sensing data

Lauren E. Gillespie[1,2,5,*], Megan Ruffley[1], Moises Exposito-Alonso[1,3,4,5,6*]

[1] Department of Plant Biology, Carnegie Science, Stanford, California, USA
[2] Department of Computer Science, Stanford University, Stanford, California, USA
[3] Department of Biology, Stanford University, Stanford, California, USA
[4] Department of Global Ecology, Carnegie Science, Stanford, California, USA
[5] Department of Integrative Biology, University of California Berkeley, Berkeley, California, USA
[6] Howard Hughes Medical Institute, University of California Berkeley, Berkeley, California, USA

[*]to whom correspondence should be addressed: gillespl@cs.stanford.edu, moisesexpositoalonso@gmail.com

This Supplemental includes:

Supplementary Text 1-5

Figures S1-28

Tables S1-13

Table of contents

Supplementary Text 4
- SM 1. Building the dataset 4
  - SM 1.1 Collecting species observations 4
  - SM 1.2 Creating Joint Observations 4
  - SM 1.3 Generating the test/train splits 5
    - SM 1.3.1 Uniform partition of dataset 5
    - SM 1.3.2 Spatial cross-validation partition of dataset 6
  - SM 1.4 Collecting Remote Sensing Imagery 6
  - SM 1.5 Bioclimatic Variables 7
  - SM 1.6 Resolution Limitations of Bioclimatic Variables 7
  - SM 1.7 Challenges in Using Open-Source Citizen Science Data 7
- SM 2. Accuracy Metrics 8
  - SM 2.1 Binary classification metrics 8
  - SM 2.2 Discrimination metrics 10
  - SM 2.3 Ranking metrics 11
- SM 3. Species Distribution Models 12
  - SM 3.1 Limitations of on-site sampling methods 13
  - SM 3.2 Convolutional neural network-based species distribution models 13
    - SM 3.2.1 *TResNet* CNN architecture 13
    - SM 3.2.2 Developing a sampling-aware loss function 14
    - SM 3.2.3 *Deepbiosphere*, a novel climate + remote sensing CNN architecture 16
    - SM 3.2.4 *Inception V3* Baseline 17
  - SM 3.3 Climate-only Species Distribution Model baselines 18
    - SM 3.3.1 Maximum Entropy Baseline 18
    - SM 3.3.2 Random Forest Baseline 18
    - SM 3.3.3 Climate-only Multilayer Perceptron baseline 18
    - SM 3.3.4 Trivial baselines 19
- SM 4. Individual species case studies 19
  - SM 4.1 Validating *Deepbiosphere's* predictions for individual species 19
  - SM 4.2 Generating high-resolution species range maps with CNN SDMs 20
  - SM 4.3 Human annotation protocols 21
  - SM 4.4 Redwoods case study 21
  - SM 4.5 Oaks case study 22
- SM 5. Mapping spatiotemporal changes with *Deepbiosphere* 22
  - SM 5.1 Detecting spatial community change 23
  - SM 5.2 Detecting temporal community change 24

Supplemental Figures 26
- Fig. S1 | Spatial and temporal resolution comparison of remote sensing data vs. SDM bioclimatic covariates 26
- Fig. S2 | Illustration of building the biodiversity dataset 27
- Fig. S3 | Biases present in the dataset and their effects on accuracy 28
- Fig. S4 | Partitioning the dataset for cross-validation 29

Fig. S5 | Comparison of SDM models' accuracy metrics across species 30
Fig. S6 | Comparison of SDM models' accuracy metrics by species rank in the dataset 31
Fig. S7 | Comparison of SDM models' accuracy metrics across latitudinal cross-validation bands 32
Fig. S8 | Generating high-resolution predictions with *Deepbiosphere* 33
Fig. S9 | Example images from human redwood labeling task 34
Fig. S10 | Humans can correctly detect mature but not young redwood groves 35
Fig. S11 | Baseline SDMs cannot detect redwoods in Tall Trees Grove 37
Fig. S12 | *Deepbiosphere*-generated species presence maps for six redwood understory and associated species 38
Fig. S13 | *Deepbiosphere* prediction breakdown by forest age for six understory species 39
Fig. S14 | Predictions of dominant species in the Santa Ynez Valley and Mountains of Southwest California 40
Fig. S15 | *Deepbiosphere*-generated species presence maps for six chaparral indicator species 41
Fig. S16 | *Deepbiosphere* prediction comparisons to baseline SDMs for six chaparral indicator species 42
Fig. S17 | *Deepbiosphere* predicts spatial community change in northern Marin county 43
Fig. S18 | Legend for fine-scale Marin vegetation map 44
Fig. S19 | Spatial community change algorithm visual explanation 45
Fig. S20 | Detecting rapid temporal plant community change after a major California wildfire 46
Fig. S21 | Example of temporal Euclidean distance calculation 47
Fig. S22 | Species range maps for a subset of well-predicted species across California. 48
Fig. S23 | Species range maps for a subset of random species across California. 50
Fig. S24 | High-resolution zoom-in of *Juniperus osteosperma* in Transverse Range. 51
Fig. S25 | High-resolution zoom-in of *Rhododendron macrophyllum* near Red Mountain, Mendocino County. 52
Fig. S26 | High-resolution zoom-in of *Calochortus argillosus* in the Bay Area region. 53
Fig. S27 | High-resolution zoom-in of *Salvia funerea* in Death Valley. 54
Fig. S28 | High-resolution zoom-in of *Collinsia torreyi* in the North Coast Mountains. 55
Supplemental Tables 56
Table S1 | Key metrics of dataset 56
Table S2 | Model summary of *TResNet* architecture 57
Table S3 | Model summary of *Deepbiosphere* architecture 58
Table S4 | Model summary of *Inception V3* architecture 61
Table S5 | Model summary of *Bioclim MLP* architecture 62
Table S6 | Including species co-occurrence and taxonomic information improves species-level prediction 63
Table S7 | Comparing the performance of different loss functions on uniform data split 64
Table S8 | Comparison of *Deepbiosphere* to baseline SDMs and previous deep-learning-based approaches 65
Table S9 | Comparison of *Deepbiosphere* to baseline SDMs on rarest species 67
Table S10 | Comparison of *Deepbiosphere* to baseline SDMs across spatial cross-validation bands. 68
Table S11 | Site details for individual species case studies and human annotation experiments 69
Table S12 | Accuracy of various SDMs on redwood + understory species in northern California 70
Table S13 | Accuracy of various SDMs on chaparral indicator species in southern California 71
Supplemental References 72

Supplementary Text

SM 1. Building the dataset

The methods for dataset collection were inspired from ref (1) and broadly consist of linking a set of species observations linked to corresponding remote sensing imagery. How the citizen science observations and remote sensing imagery were collected is detailed below.

SM 1.1 Collecting species observations

We collected observations from kingdom *Plantae* using *GBIF.org* from the years 2015-2022 (2). Only records observed by humans with a coordinate uncertainty radius of less than or equal to 120m with no flagged geospatial issues were taken from within the state of California. Nearly all of the subsequent observations were public observations uploaded using the *iNaturalist* app. Any person with a smartphone and who has downloaded the app can upload observations to *iNaturalist*, meaning that the observations used in this dataset were collected by many thousands of citizen scientists with a wide variety of backgrounds. Accordingly, some observations may be mis-identified.

To minimize misidentification, only research-grade observations with taxon identifications from at least two community members were included in the dataset. That being said, mis-identified observations or mis-located observations can still slip past these filters, making this data especially challenging to work with (3, 4). However, GBIF takes steps to resolve major mis-identification events between closely related taxa (4). Finally, a recent case study from San Clemente Island in California showed that all examined *iNaturalist* observations with a positional error of < 10m as listed in GBIF were within 270m of the corresponding species detected from remote sensing imagery (3). Extrapolating to this dataset, it's reasonable to assume that a <120m positional uncertainty filter would place the vast majority of observations within the linked remote sensing image, therefore preserving the geographic relationship between the observed species and the remotely sensed image.

In total we downloaded a total of 912,380 plant observations of 5,193 unique plant species (2). We further filtered observations to only include vascular plants, which we define vascular plants as all plants in the taxonomic classes of Gnetopsida, Liliopsida, Lycopodiopsida, Magnoliopsida, Pinopsida, Polypodiopsida, Lycopodiopsida, and Ginkgoopsida. We also removed duplicate observations of the same species within a 150 m radius, removed species that contain all observations located within a 256 m radius, and were not geographically located within the Global Administrative Area boundary of California, or were missing climate or NAIP imagery data. To increase the density of observations in the dataset, we used neighbor imputation to add any other species observed within an overlapping 256m radius to a given observation (**SM 1.2**, **Fig. S2**). We finally removed any species that had fewer than 500 total observations in the dataset after neighbor imputation, leaving us with a total of 652,027 observations of 2,221 unique plant species (**Table S1**).

SM 1.2 Creating Joint Observations

To create the joint species occurrence dataset, we used neighbor imputation of locally overlapping observations. Specifically, for each observation, we appended any species observed nearby within a fixed radius to said observation (**Fig S2**). Observations were considered to be locally overlapping if the Euclidean distance between the latitude and longitude of the two points is less than or equal to 256 m. While this technically means that some neighbor observations may not be geographically located within a neighbor's 256 × 256 pixel image, said resolution is on par with accepted spatial scales in theoretical biogeography

and empirical community ecology, which have shown that biotic species interaction networks between individual plants can reach scales of thousands of square meters, and both biotic and shared land use features are thought to drive site-level plant distribution (5–7). Therefore, although there is no strict guarantee that two observations lie within the extent of their overlapping observations' respective NAIP imagery, strong ecological theory supports that the two species may be influencing each other's co-occurrence and subsequent observed co-occurrence.

The reason that we chose to create a joint dataset is twofold. First, there is theoretical evidence to support that biotic interactions (the interactions of two living species both directly and indirectly) are a strong driving factor in the distribution of species at a variety of scales (6) and that overlap data can be seen as a partial observation of these biotic interactions. Second, many species in our dataset have few observations (**Fig. S3A**) which can make it impossible to learn an accurate representation of the species' distribution. However, oftentimes these rarely observed species will inhabit similar habitats to much more commonly observed species. Therefore, building a multi-species observations dataset may enable better modeling of those rare species using shared habitat signatures learned for the more common, overlapping species.

Along with providing overlap data, we also provide higher taxonomic information per-image. Concretely, the species, genus, and family of all overlapping species was utilized for training the *TResNet* and *Deepbiosphere* models. Our rationale was that the phylogenetic history embedded within the taxonomic hierarchy of species should also encode a shared ecological niche space for some taxonomic groupings. However, depending on the study system, models can be trained without these options by including the flag `--dataset_type = single_species` during training to train models without co-occurrence information and `--taxon_type = speconly` during training to train models without higher order taxonomic information.

SM 1.3 Generating the test/train splits

In order to properly validate and compare models, we split the dataset into multiple partitions. Best practices to ensure the reproducibility of machine learning models is to have a train-test-validation split, ideally with the validation data coming from a separate acquisition process to provide as robust a test as possible. To test models with statistical power, such validation sets on the order of thousands of observations would be necessary. Furthermore, to test our models appropriately, we require test and validation sets that are at least 1,300 m away from all training set examples, to prevent data leakage due to spatial autocorrelation in the coarse resolution Bioclim data used by baseline models. Next, since the CNN predicts at 256 m resolution, observations with low geographic uncertainty are a must. Finally, since citizen science observations tend to be common on publicly accessible land and near major access routes, candidate locations for taking validation observations tend to be located on private, inaccessible land or in very remote locales that are exceedingly difficult to reach on foot. Understandably, finding or collecting such a validation dataset is an exceedingly challenging task and is left for future work. Having those caveats in mind, to robustly test the models with the data we have, we designed two types of test/train splits to pick hyperparameters and test extrapolation accuracy: The first type—the uniform split—was used primarily for choosing an optimal loss function and learning rate, while the second type—spatial cross-validation—was used to validate these choices and test model extrapolation ability.

SM 1.3.1 Uniform partition of dataset

The first partition—the uniform split—was generated by randomly selecting observations uniformly from across the state (**Fig. S4A**) which we refer to as the *uniform partition* and use the notation $modelname_{unif}$ to refer to models trained using this partition of the dataset. We chose points uniformly across the state to maximize the number of unique climates where models would be evaluated on. To ensure the independence of training and testing set data due to spatial autocorrelation, we added all overlapping observations to the test set to guarantee that none of the remote sensing images and observations in the test set were present in

the training set. To ensure that there was no data leakage between the test and train set, only observations which were more than 1,300 m away from any other non-overlapping observation were included. We chose an exclusion radius of 1,300 m because the climate variable raster pixels converted from arc-seconds to meters can have a diameter of up to 1,200 m, so any test set observation within that distance to any observation in the train set would have an identical input value as some observations used during fitting, resulting in data leakage. Ultimately 1.88% of the dataset was set aside for testing using this method. The test dataset has relatively few observations compared to a traditional 80/20% test/train split because the *iNaturalist* observations are very spatially heterogeneous, and tend to be very clustered, meaning that there are few observations that are sufficiently far enough away from observations used for training to be included in the test split.

SM 1.3.2 Spatial cross-validation partition of dataset

In order to provide cross-validation of the uniform train-test split and to test the extrapolation ability of all models, we also conducted a latitudinal ten-fold spatial holdout block validation by partitioning California into ten one-degree latitudinal bands (**Fig. S4B**) which we refer to as the *spatial partition*, using the notation $modelname_k$ to refer to models trained using points from the k-th spatial block (**Fig. S4B**). Training points within 1,300 m of the test band were removed to prevent data leakage as discussed above (For models utilizing pseudo-absence points, all pseudo-absence points within the test bands were removed to ensure a fair comparison to presence-only models). Ultimately, the percentage of test points per-spatial block ranged from 1.40-25.35% of the entire dataset.

SM 1.4 Collecting Remote Sensing Imagery

To link species observations with images, we utilized aerial imagery from the National Agricultural Imagery Program (NAIP) (8) which we downloaded for the entire state of California from 2012 and 2014 using Microsoft Azure's NAIP data blob disk image on its West Europe and Eastern U.S. servers. This dataset was chosen as it his highly pre-processed and curated, containing sun angle-corrected orthophotography data collected during the leaf-on growing season with guaranteed < 10% cloud cover at 1 m-resolution (see ref. (9) for a comprehensive overview of NAIP data). For training the CNN models, we specifically used the NAIP data from 2012 at 1-m resolution to generate 256 x 256 pixel images, where 1 pixel corresponds to a 1 x 1 m resolution. We used all available bands for training, specifically the RGB and infrared color bands (**Table S1**). The 256 x 256 pixel images were extracted so that the geographic coordinates of the corresponding species observation mapped to the center of the image (**Fig. S2**).

Our decision to work with four-band RGB-Infrared remote sensing data instead of many-band Landsat data or full-band hyperspectral data was motivated by data accessibility and resolution to capture biological patterns. Although Landsat data is collected worldwide, it has a pixel resolution of 30 m per-pixel, while NAIP data has a 1m-60cm pixel resolution (depending on the year of acquisition). Both Landsat and NAIP are sub-kilometer resolution, but the higher resolution of NAIP data better captures important local factors for plant distribution modeling such as individual tree crowns, land use, and biotic interactions. Further, the four color and infrared bands (RGB-I) of NAIP also contain the same information as derived vegetation products such as NDVI (10). Finally, other hyperspectral remote sensing products with a similar level of resolution to NAIP—such as the 224-band, full-spectrum product AVIRIS—have limited coverage at national scale, not even covering all of California. Indeed, four-band (Red-Green-Blue-Infrared) remote sensing products of a similar resolution are available from private companies like Maxar Technologies and Planet Labs across the entire globe with weekly to nearly daily acquisitions. Therefore, the four-band NAIP imagery provides an optimal mix of spatial resolution and availability to appropriately model plant communities at a theoretically sound scale with the ability to scale these techniques to potentially the rest of the globe.

SM 1.5 Bioclimatic Variables

We used the 19 bioclimatic variables available from WorldClim Version 2 at 30 arc-second (approximately 1km) per-pixel resolution (11). Variables were downloaded directly from the WorldClim Version 2 repository (http://www.worldclim.com/version2). Before fitting any model, all bioclimatic variables were normalized per-variable to mean 0 and standard deviation of 1 using the entire raster clipped to the outline of California.

SM 1.6 Resolution Limitations of Bioclimatic Variables

While downscaling and interpolation techniques exist that allow one to scale bioclimatic variables to 250-100 meter resolution (12, 13), these downscaling and interpolation methods are inherently limited in the amount of novel information they can provide, and introduce a new source of potential bias (14). Furthermore, there has been evidence that SDMs trained using coarser-grained climate data overestimate some montane species' tolerance to changing climate compared to meter-resolution local scale information (15). This discrepancy can be easily seen in **Fig. S1** which illustrates the difference between the remotely sensed NAIP imagery and Bioclim in northwest California. One can easily distinguish the various grassland-forest transitions along with land use differences from agriculture. This local information is simply not detectable from the projected 7th band of Bioclim data shown. The 30 x 30 arc-second pixels of the bioclimatic rasters are simply too coarse-resolution to capture the local variations between the various communities in this image, as evidenced by the fine scale vegetation map for the region (**Fig. S1B**). Furthermore, from ecological theory it is expected that fundamental drivers of species distribution should be different at local versus site scale, with bioclimatic data driving local scale distribution versus topographic and land use information driving site scale distribution (7). Thus, it is to be expected that the information contained in both sources to be radically different.

SM 1.7 Challenges in Using Open-Source Citizen Science Data

Creating citizen science-based species distribution models is challenging due to the unevenness of number of observations per species and data quality issues. Despite our data filtering, which included minimum data thresholds for species to be included in the analyses, we still see many dataset imbalances (**Fig. S3**); for example, 20% of species have 1,000 images or less while only 27% of species have 10,000 or more. In addition, 37,300 images contain only one species while only 61,386 images have >100 species, reaching the expected size of a full species checklist for a given 256 m radius area. Moreover, species labels are presence-only, meaning that in most cases, the absence of a species label in an image does not guarantee true absence from the ecosystem, only that it has not been observed on *iNaturalist* at that location. Overall, this leads to a highly imbalanced dataset, with some habitats and some species underrepresented and hard to study.

Specifically, our dataset of observations suffers from three main types of biases. First, there's spatial bias, in that observations are not uniformly distributed across the landmass of California (**Fig. S3C**). Citizen scientists can only take observations where they can themselves access species, which restricts most observations to publicly owned land and convenience plays a large factor in the distribution of observations, with more observations coming from ecoregions with a higher population or more public parks, such as the Southern California Chaparral or Coast Range ecoregions (**Fig. S3D**).

Second, observations from casual users tend to show density bias (**Fig. S3B**), where many observed locations have few other overlapping species reported, while a few observations have many overlapping species reported. Oftentimes, this is a result of observers noticing and documenting a particularly salient individual of a species, like when a specific wildflower is in bloom in the spring. However, rarely will users

upload all the plant species they may find in a given small area, meaning that the majority of species present at any given observation location in our dataset are unreported. These unobserved species are referred to as *pseudo-absences*. The high pseudo-absence rate of our dataset also means that we cannot consider these species occurrences to represent full species checklist data at each site in our dataset, meaning that the co-occurrence network of each site in our dataset is partially observed, again adding extra challenge to the machine learning task.

Third, the dataset is also long-tailed in the number of observations per-species, with many species possessing few observations while a few possess a large number of observations in the dataset (**Fig. S3A**). Unfortunately, this "commonness of rarity" is a known phenomenon in plants' distributions and is an expected phenomenon for plant observations (16). This class imbalance can be problematic for classic machine learning algorithms, since standard accuracy metrics become less informative, and models can simultaneously suffer from both under- and over-fitting across classes (17). However, it should be noted that these challenges are not unique to this dataset alone (18–20), thus algorithms successfully able to learn a generalizable representation of this data should be of interest to both the species distribution modeling and more general machine learning fields.

Despite these challenges, citizen science provides an opportunity to utilize deep learning methods that may require hundreds of thousands to millions of observations to train. Further, the wide array of learning functions and training techniques for deep learning models can still enable the learning of useful patterns even with this complex dataset.

SM 2. Accuracy Metrics

We report twenty accuracy metrics from across a variety of relevant disciplines, from computer vision to species distribution modeling. The reported accuracy metrics can be classified into three broad categories, which are explained below.

SM 2.1 Binary classification metrics

Binary classification metrics are a very common set of metrics used to compare yes/no binary prediction tasks (or e.g. whether a species is or is not present at a given location). For binary classification-based metrics, probability predictions must be converted to a binary presence / absence output using a threshold value. There is vigorous debate within the SDM community on the proper threshold to pick for presence versus absence (21, 22), but for consistency with the computer vision community (as sigmoid transformations are common in many computer vision loss functions, and thus positive values map to above 0.5 and negative values map to below 0.5), and the fact that our multi-label domain makes optimal threshold determination non-trivial, we chose to threshold all probabilities ≥0.5 as present and <0.5 as absent for these metrics. Specifically, for all reported binary classification metrics in the main text, figures, tables, and supplemental (precision, recall, *F1*, presence accuracy), we always use the reported 0.5 threshold. To reiterate the rationale, a 0.5 threshold not only a common threshold in species distributions, but it is a standard threshold used by the computer vision community, as when using a sigmoid-based loss function, values above 0.5 map to positive real-valued numbers and values below 0.5 map to negative real-valued numbers.

There exists many different metrics for binary classification, but in this work we focus on four common ones: *precision*, which measures how many species predicted to be present were actually present; *recall*, which measures how many of the true species present are predicted as present; *F1*, which is the harmonic mean of precision and recall and represents a conservative mean of the two (i.e. is more affected by low values); and *accuracy* which simply measures the percent of correctly identified examples of present

species. For each metric, both binary and multi-class versions exist, along with single-label and multi-label. In this work, we report the multi-label, multi-class versions of each metric except for accuracy, which is reported as multi-class, single-label. To clarify this technicality, we refer to accuracy as *presence accuracy* in this work to signal that for each example the model can either be right or wrong, as there is only one species tested at a time.

In the multi-label, multi-class setting, there are multiple axes by which one can aggregate the chosen statistic: the first is by species, which we refer to as *per-species*; the second is by example image, which we refer to as *per-image*. For all metrics, S is the number of unique species in the training split of the dataset, N the number of images in the training split of the dataset, $\bar{y}$ is the multi-label ground truth neighbor-imputed presences and absences of each species for each image, y_s is the single-label original species associated with each observation, $\hat{y}$ is the SDM's predicted binary present / absent list for each species and each image using a 0.5 threshold, tp is true positives, fn is false negatives and fp is false positives (N.B. true negatives are unknown). Per-species metrics were calculated using scikit-learn version 1.1.1 (23) and per-image metrics were implemented by ourselves (see open Github repository for implementation: github.com/moiexpositoalonsolab/deepbiosphere).

$$\text{per-species recall} = \frac{1}{S}\sum_{i=1}^{S}\frac{tp_i}{tp_i + fn_i}$$

$$\text{per-species precision} = \frac{1}{S}\sum_{i=1}^{S}\frac{tp_i}{tp_i + fp_i}$$

$$\text{per-species F1} = \frac{1}{S}\sum_{i=1}^{S}\frac{2 \cdot Rec_i \cdot Prec_i}{Rec_i + Prec_i}$$

For per-image accuracy metrics, we used the definitions as outlined in ref. (24) using a custom implementation written in Python.

$$\text{per-image precision} = \frac{1}{N}\sum_{i=1}^{N}\frac{|\bar{y}_i \cap \hat{y}_i|}{|\hat{y}_i|}$$

$$\text{per-image recall} = \frac{1}{N}\sum_{i=1}^{N}\frac{|\bar{y}_i \cap \hat{y}_i|}{|\bar{y}_i|}$$

$$\text{per-image F1} = \frac{1}{N}\sum_{i=1}^{N}\frac{2 \cdot Rec_i \cdot Prec_i}{Rec_i + Prec_i}$$

Finally, for the presence accuracy, we use the standard multi-class definition, defined as the fraction of examples where the correct species observed at that location was predicted as present. This metric was calculated using our own custom Python implementation.

$$\text{presence accuracy} = \frac{\sum_{i=1}^{N} y_{i,s} = \hat{y}_{i,s}}{N}$$

SM 2.2 Discrimination metrics

Binary classification metrics do come with some drawbacks, mainly that the choice of what threshold value to use for determining whether a species is present can have an outsized impact on the accuracy of a model (25). In order to take into account the effect of thresholds, discrimination metrics measure binary classification ability across a wide range of thresholds in order to calculate an SDM's performance across a wide range of presence thresholds and describe the relationship between threshold change and performance change. Rather than set an arbitrary threshold, they observe how well the model is able to predict across the range of predicted probabilities for that class to see if a higher threshold means more species are correctly predicted without adding in too many false positives. In other words, discrimination metrics (AUC_{ROC}, AUC_{PRC}, and calibrated AUC_{ROC} & AUC_{PRC}) essentially integrate accuracy across a gradient of presence thresholds and negate the need for choosing a specific threshold value. This is why AUC is a very common metric used to select species distribution models and other classification approaches and is why it is the accuracy metric of choice when comparing models in this work.

For discrimination metrics, we report the area under the receiver operating characteristic curve averaged across species (AUC_{ROC}) and average area under the precision-recall curve averaged across species ($AUC_{PRC\mathit{spp}}$). We again use multi-label, neighbor imputed ground truth presences and absences when calculating discrimination-based metrics. We use scikit-learn version 1.1.2 for all discrimination metrics, which utilizes the trapezoidal integration to calculate area under the curve (23). $tp(\hat{y_s}, i)$ refers to the number of true positive predictions of species s in $\hat{y}$ when using i as the threshold for predicted presence for species S, while $fp(\hat{y_s}, i)$, and $fn(\hat{y_s}, i)$ are the same for the number of false positives and false negatives, respectively. $P(\bar{y_s})$ is the true number of actual presences in the ground truth for species S, which can also be written as $\sum_1^N \bar{y_s}$and $N(\bar{y_s})$ is the true number of actual absences in the ground truth data for species S, which can also be written as $\sum_1^N 1 - \bar{y_s}$.

$$\text{average } AUC_{ROC} = \frac{1}{S}\sum_{s=1}^{S} \int_{i=\min(\hat{y_s})}^{\max(\hat{y_s})} \frac{1}{2} \cdot \Delta TPR(\hat{y_s}, i, \bar{y_s}) \cdot \Delta FPR(\hat{y_s}, i, \bar{y_s})$$

$$\text{average } AUC_{PRC} = \frac{1}{S}\sum_{s=1}^{S} \int_{i=\min(\hat{y_s})}^{\max(\hat{y_s})} \frac{1}{2} \cdot \Delta Prec(\hat{y_s}, i) \cdot \Delta Rec(\hat{y_s}, i)$$

$$\Delta Prec(\hat{y_s}, i) = \frac{tp(\hat{y_s}, i+1)}{tp(\hat{y_s}, i+1) + fp(\hat{y_s}, i+1)} - \frac{tp(\hat{y_s}, i)}{tp(\hat{y_s}, i) + fp(\hat{y_s}, i)}$$

$$\Delta Rec(\hat{y_s}, i) = \frac{tp(\hat{y_s}, i+1)}{tp(\hat{y_s}, i+1) + fn(\hat{y_s}, i+1)} - \frac{tp(\hat{y_s}, i)}{tp(\hat{y_s}, i) + fn(\hat{y_s}, i)}$$

$$\Delta TPR(\hat{y_s}, i, \bar{y_s}) = \frac{tp(\hat{y_s}, i+1)}{P(\bar{y_s})} - \frac{tp(\hat{y_s}, i)}{P(\bar{y_s})}$$

$$\Delta FPR(\hat{y_s}, i, \bar{y_s}) = \frac{fp(\hat{y_s}, i+1)}{N(\bar{y_s})} - \frac{fp(\hat{y_s}, i)}{N(\bar{y_s})}$$

One major drawback to this approach is that it does not measure the calibration of an SDM's predicted probabilities well, meaning that a model which has extremely low predicted probabilities can still nevertheless have a high AUC_{ROC} if within its range of predicted probabilities said model has good

sensitivity and specificity for that class. This means that it's possible to have a model that never predicts a species as present with the standard presence/absence threshold of 0.5 yet still has a high average AUC_{ROC}. Furthermore, tuning the presence/absence threshold using the ROC curve for such models is non-trivial, since the derived optimal threshold will likely be different across species. To correct for this, we also introduce a calibrated area under the curve score where the chosen thresholds are linearly interpolated values between 0 and 1. We use a trapezoidal approximation of area under the curve, utilizing scikit-learn's implementation of area under the curve with 50 uniformly spaced probability thresholds between 0 and 1 (23).

$$\text{calibrated avg. } AUC_{ROC} = \frac{1}{S}\sum_{s=1}^{S}\int_{i=0}^{1}\frac{1}{2}\cdot\Delta TPR(\hat{y_s}, i, \bar{y_s})\cdot\Delta FPR(\hat{y_s}, i, \bar{y_s})$$

$$\text{calibrated avg. } AUC_{PRC} = \frac{1}{S}\sum_{s=1}^{S}\int_{i=0}^{1}\frac{1}{2}\cdot\Delta Prec(\hat{y_s}, i)\cdot\Delta Rec(\hat{y_s}, i)$$

SM 2.3 Ranking metrics

Compared to binary classification and discrimination metrics, ranking metrics focus solely on how high a given species is ranked by probability of presence compared to other species in the same image / observation. These ranking metrics suffer from the same limitations as the aforementioned discrimination metrics in that they only compare accuracy of probabilities in a relative sense, rather than the absolute. However, they are the most common within the deep learning and computer vision communities, so we choose to report them here for completeness. The first set of ranking-based metrics we report are top-K accuracy metrics, a set of single-label metrics. These metrics measure how many times the correct species was correctly predicted within the top-K highest-ranked species within a given image (where, for example, K=5 would be the 5 species with the highest probability of presence). Much like the binary classification metrics, top-K accuracy can be calculated across images and also across species (which we refer to as Top K_{img} and Top K_{spp}, respectively). However, top-K accuracy across species is considered to be a better metric of an SDM's ability to distinguish present species, as it corrects for sampling imbalances across species (1, 4).

Machine learning papers oftentimes report top-1 or top-5 accuracy, but given our task is an inherently multi-label one, we choose to report with a larger K than normally seen in computer vision projects with many possible labels, as the expected number of unique plant species at the local scale varies anywhere from five to one hundred and thus on average we are most interested in the composition of these top five to one hundred species. To that note, we report both Top-K_{img} and Top K_{spp} for K = 1, 5, 30, and 100. Along with top-K accuracy, both top-K recall and precision also exist but are far less commonly reported and so we do not report them here. We implement these metrics in Python using the definitions from ref (1). Here, $\text{rank}(j, \hat{y}_i)$ is defined as the rank of species j observed in image i from the sorted list of probabilities $\hat{y}_i$ predicted by the SDM, $\text{rank}(j, \hat{y}_i) = \|\{k : \hat{y}_{i,k} \geq \hat{y}_{i,j}\}\|_0$.

$$\text{Top-K}_{img} = \frac{1}{N}\sum_{i=1}^{N} Acc(\hat{y}_{i,j}, K)$$

$$\text{Top-K}_{spp} = \frac{1}{S}\sum_{i=1}^{S} Acc_{spp}(\hat{y}_{i,j}, i, K)$$

$$\text{where } Acc_{spp} = \sum_{m=1}^{N_i} Acc(\hat{y}_{m,j}, K) \text{ and where } Acc = \begin{cases} 1 \text{ if rank}(j, \hat{y}_i) \leq K \\ \\ 0 \text{ otherwise} \end{cases}$$

However, as a single-label metric, these top-K accuracy metrics do not use or capture any information about an SDM's ability to correctly label overlapping species, making them less useful for judging an SDM's ability to capture co-occurrence patterns correctly. To capture multi-label ranking performance, there exists a commonly-used multi-label ranking-based metric called mean average precision (mAP). This metric is also sometimes referred to as label ranking average precision (LRAP). mAP calculates how highly each species is correctly ranked along with how many other present species are ranked higher, averaged across species. mAP is the multi-label version of the mean reciprocal rank metric (MRR) commonly used in document retrieval.

$$\text{mAP} = \frac{1}{N} \sum_{i=1}^{N} \frac{1}{\|\bar{y}_i\|_0} \sum_{j:\bar{y}_{i,j}=1}^{J} \frac{\|L(\hat{y}_{i,1:j})\|_0}{\text{rank}(j, \hat{y}_i)} \text{where } L = \begin{cases} 1 \text{ if } \hat{y}_{i,k} \geq \hat{y}_{i,j} \\ 0 \text{ otherise} \end{cases}$$

SM 3. Species Distribution Models

Species distribution models (SDMs) describe how a species is distributed across a given geographic extent. Oftentimes this is accomplished by modeling how the predicted presence of a species across a landscape varies spatially in response to a set of ecologically-meaningful variables. SDMs broadly fall into two rough categories: process-based and correlative. Process-based models attempt to derive fundamental equations based on processes or mechanisms governing a species distribution and build a model of likelihood of occurrence (e.g. dispersal ability, growth rates, demographic characteristics, etc.). Correlative models attempt to infer a species' geographic extent by correlating its known occurrences with a suite of relevant environmental variables and projecting likelihood of occurrence from said correlated variables. However, these definitions are not dichotomous nor mutually exclusive, meaning correlative models may indeed capture some process-based mechanisms intrinsically in their modeling procedure (26).

Further distinction can be made between single species and joint species SDMs, with the former only modeling one species at a time and the latter attempting to model multiple species' distributions simultaneously. Joint SDMs can be further subdivided based upon at what point in the modeling process the species were aggregated. Some joint SDMs are really aggregation of individual SDMs, with the predicted species' presence joined during *post-hoc* analysis (27), while other joint SDMs model all species simultaneously throughout both the model fitting and analysis steps (28). For future clarification, when referring to a *joint SDM*, we are referring to the latter process.

A final important distinction between different types of SDMs is between functional niche models and realized niche models. The functional niche of a species traditionally was defined as the set of environments where a species individually can sustain itself, while the realized niche is the set of environments where a species can sustain itself in the presence of competition from other biotic sources (29). Contemporary niche theory has strengthened these definitions to include dispersal dynamics, growth rates, and biotic interactions, all vital processes to a species' dispersal (30). However, many modern uses of the terms use a simpler less precise definition of niche that simplifies the fundamental niche to where a species *can* occur and the realized niche to where a species *does* occur (31, 32). Our approach falls somewhere in between, where for some species, like large redwoods, the realized nice is likely being modeled through direct detection of redwood canopy signatures, while for smaller less-observable species, like redwood sorrel, the modeling process is closer to the functional niche than realized.

SM 3.1 Limitations of on-site sampling methods

While the best metric of species presence and current biodiversity comes from on-the-ground observation of species, generating comprehensive checklists of species presence at high resolution across large geographic extents is generally infeasible. For example, in ref. (33), rarefaction curves of species richness were generated at various grid sizes and using estimates from this analysis of over 1.5 million plant observations, an estimated 1,000 observations would need to be taken per grid scale at a 15 km resolution to reach a point where the rarefaction curve begins to plateau, with many cells requiring upwards of a magnitude more samples due to California's extremely heterogeneous and endemic plant communities. Using the 1,000 observation estimations, an conservative estimated uniformly sampled 2 million checklists would be needed to estimate species richness at 15 km spatial blocks, with rapidly increasing numbers of samples as the desired resolution increases. All in all, it would be infeasible to try and perform the same species checklist curation using onsite methods that *Deepbiosphere* can perform.

SM 3.2 Convolutional neural network-based species distribution models

Convolutional neural networks (CNNs) are a popular machine learning model that have been adapted to successfully model a wide variety of complex, real-world image-related tasks, from image classification to deciphering handwriting (34, 35). Their widespread success lies in their ability to learn and extract arbitrary patterns and features from images without any human input, allowing them to detect and exploit only the most relevant visual features of an image for a given task. This ability to intrinsically learn the most relevant features of an image makes them very flexible for use across a wide variety of image-related tasks and a wide range of image media, from medical imagery of cells to satellite imagery (36, 37). In this work, we seek to predict the presence of thousands of plant species from relevant features found in the local aerial imagery of a location, a task that CNN models are well-designed to perform.

We implemented all of our deep learning models in the standard deep learning framework *PyTorch* and implementation details can be found in the associated repository (github.com/moiexpositoalonsolab/deepbiosphere). All CNNs were trained with standard mini-batch stochastic gradient descent for 13 epochs using the Adam optimizer. The epoch of evaluation was determined using early stopping calculated from the per-species average area under the receiver operator characteristic curve (AUC_{ROC}) on the uniform test set split (see **SM 2.2** for metric details). Learning rates were tested using a stepwise sweep ranging from $5x10^{-6}$ to $1x10^{-1}$ in increments of 0.5 and batch sizes were chosen depending on model size relative to the GPU size used for training. Batch size, learning rate, memory usage, and GPU architecture used for training are reported for each CNN in **Tables S2-S10**.

SM 3.2.1 *TResNet* CNN architecture

For training CNN-based SDMs using NAIP remote sensing imagery as input, we chose to use the medium-sized *TResNet* architecture, a small CNN-based residual neural network (38) which is GPU-optimized for fast inference speeds and is a state-of-the-art architecture for multi-label image classification in the computer vision community (39). We modified the *TResNet* architecture to have four input channels in order to support the RGB + Infrared NAIP imagery. Following on previous work that suggests higher-order taxonomic signals are useful for species-level classification (40), we conducted an ablation study using the uniform test split of the dataset (**SM 1.3.1**) that compared the classification performance of the *TResNet* architecture using just the present species labels to the same architecture using higher-order taxonomic information to make predictions, specifically species, genus, and family labels (**Table S6**). We found that adding higher-order taxonomic information improved performance, and thus included both species, genus, and family classification during training in all subsequent *TResNet*-based experiments (**Table S2**).

Following on previous work that found using species co-occurrence signals when training deep neural networks for species distribution modeling substantially improves performance (41), we also performed

another ablation study on the uniform test split of the dataset where we compared model accuracy using just the original species in each observation (single-label classification) to using all nearby imputed neighboring species (multi-label classification) (**Table S6**). We found that including nearby species information again improved performance, and thus subsequently used the nearby species information in all subsequent *TResNet* analyses when possible.

All model weights were initialized following best practices laid out in the original *TResNet* paper, using Kaiming He-style for CNN layers and zeroed out BatchNorm and residual connections (38). For all analyses, the *TResNet* outputs were converted to independent probabilities using the sigmoid transformation. Using the taxonomically-informed architecture and nearby imputed species, we performed a linear learning rate optimization sweep, testing a series of static learning rates from 0.01 to $1x10^{-6}$ in increments of 0.5. We found a learning rate of $1x10^{-5}$ had the highest accuracy on the uniform test set for most *TResNet*-based models and was the chosen learning rate for all subsequent experiments.

SM 3.2.2 Developing a sampling-aware loss function

We compared performance of our modified *TResNet* architecture trained on a variety of common loss functions and a new sampling-aware loss function we developed (sampling-aware binary cross-entropy [BCE]) using the uniform test split of the dataset (**Table S7**). Specifically, the loss, which determines how correct the CNN model's prediction is for a given example, is task-dependent and there are many different choices for a given task type. In this work, we frame our problem as a classification task, where the goal is to classify each image into one of *N* classes. We also frame our problem as a multi-label task, which extends the above definition to classify each image into *K* of *S* classes (where *K* is the number of present species in the observation). While many CNNs have been developed for image classification, the vast majority of these architectures have been designed for *single-label classification*, where for each image exactly one class should apply; for example, each image may either have a dog or a cat, but an image is never expected to have both. However, what makes our dataset both unique but challenging is that it provides occurrences of all overlapping species in a given image, making it a *multi-label* dataset (see Section 1.2 for more details), since each image is associated with anywhere from one to nearly one hundred overlapping plant species within 256 × 265 m squares.

Notationally, single-label data refers to training models with examples where only the original species observed at that location is included as a positive label, with all imputed neighbors being ignored. Alternately, multi-label data refers to including all imputed neighbor species as positive labels in each training example in addition to the originally observed species. Following, the notation $\hat{y}$ refers to the raw outputs from a neural network, $\bar{y}$ refers to the single- or multi-hot vector of known species presence in that example, where 0 means a species is absent and 1 means a species is present, and S is the number of unique species in the dataset.

The most commonly used loss function for training classification CNNs in a single-label setting is cross-entropy loss (CE).

$$\text{CE} = -L_{+} - L_{-} \begin{cases} L_{+} = \bar{y} \cdot log(f(\hat{y})) \\ L_{-} = (1-\bar{y}) \cdot log(1-f(\hat{y})) \end{cases} \quad \text{where} f(\hat{y}_i) = \frac{e^{\hat{y}_i}}{\sum_j^S e^{\hat{y}_j}}$$

It should be noted that softmax-based losses like CE loss were designed for classification of single-label datasets and model a probability density function across labels (35) (meaning the sum of probabilities across all possible labels must be 1 per-example). However, this approach does not match the multi-label nature of our task well, as in each image the probabilities across species should be independent so that the presence of one species in an image does not imply a decrease in presence of other species. Furthermore, when using a softmax-based transformation of model outputs, probabilities of an individual species are no longer

directly comparable across individual observations on account of the lack of independence across labels, and thus is not a valid transformation for calculating binary classification metrics. Nevertheless, to compare to previous work in ref. (42), we still report accuracy metrics for models trained with CE loss using the softmax transformation.

In the multi-label setting, the classic loss used for training CNNs is binary cross-entropy (BCE) loss:

$$\text{BCE} = -L_{+} - L_{-} \begin{cases} L_{+} = \bar{y} \cdot log(f(\hat{y})) \\ L_{-} = (1-\bar{y}) \cdot log(1-f(\hat{y})) \end{cases} \quad \text{where} f(\hat{y_i}) = \frac{1}{1+e^{-\hat{y_i}}}$$

BCE loss can be intuitively interpreted as training the neural network to maximize the conditional log-likelihood of species presence and thus the network's predictions can be interpreted as estimating the likelihood of species occurrence in any arbitrary image. However, our dataset exhibits two important types of imbalances that make this standard loss not ideal for our task. First, our dataset exhibits strong observation imbalance, with few species possessing many observations and many species possessing few observations (**Fig. S3A**) which is problematic since the standard BCE loss formulation assumes an equal number of observations per-class in the dataset. The second is label imbalance, or the imbalance in the number of present vs. absent species per-image, a byproduct of using citizen science observations with incomplete coverage of all species present in a given 256 × 256 m area (see **SM 1.7** for details). Most observations have fewer than five species present in the observation (**Fig. S3B**), and very few observations reach close to the expected true number of plant species present in a given 256 m radius. Therefore, in most cases only one to one hundred classes from the 2,221 species are present in a given observation and it should be assumed that all locations contain some pseudo-absences in $\bar{y}$. Since BCE loss assumes all absences are true absences, yet pseudo-absences are guaranteed to be present in the dataset, a loss calculation whose main contributions are from absences points is not ideal for our task.

Therefore, we also considered two other losses that handle the contribution of the negative class differently. The first is a recent multi-label classification loss variant of focal loss called asymmetric focal loss (ASL) (43). This loss was explicitly designed and optimized for multi-label tasks by upweighting the loss contributions of the present classes without eliminating the contribution of the absent classes entirely. It does so by breaking the standard BCE definition down per-class, depending on whether the class is present or absent in the observation. The contribution of the absent versus present classes is then differentially applied to the loss using the hyperparameters γ_{+} and γ_{-}. By setting $\gamma > 0$, the loss contribution of all absent classes will be scaled down, decreasing their contribution to the overall loss. Furthermore, very easy negative examples which the network assigns low probability ($\hat{y}_i \ll 0.5$) will be exponentially down-weighted, creating a "soft thresholding" effect.

The loss contribution of absent classes can be further reduced for easy negatives through the addition of "hard thresholding" (the function p_m) which fully discards the loss contribution for absent classes with predicted probability below a tunable threshold, m. One can think of this as "throwing away" the loss contribution of very easy classes ($\hat{y}_i \lll 0.5$). Furthermore, the shape of loss function is such that very hard negative samples ($\bar{y}_i = 0$ when $\hat{y}_i \approx 1$) have a down-weighted loss contribution as well (see Fig. 3 of ref (43) for specifics). This corresponds to the scenario where an observation is "mis-labeled," which in our dataset would correspond to a location missing an observation of a clearly present species, thus minimizing the negative effect of density bias seen in our dataset. Finally, the values of γ can be dynamically adjusted during online training to maintain symmetry between the probability contribution of absent versus positive classes, to ensure that the loss contribution of negative samples does not "overwhelm" the contribution of the positive classes. Overall, these benefits fit our dataset well.

This soft and hard thresholding is somewhat analogous to the sampling of pseudo-absence points, a required step for the maximum entropy modeling of *Maxent*, but importantly the hyperparameters are not dependent

on the spatial extent of a given species, as are the pseudo-absence sampling ranges of *Maxent*, and are instead a product of the distribution of presences versus absences in the dataset, thus avoiding the biases introduced through *Maxent* sampling process (44). We used the recommended default values for $\gamma^{+} = 1$, $\gamma^{-} = 4$ and $m = 0.05$, the default values proposed in ref. (43).

$$\text{ASL} = -L_{+} - L_{-} \begin{cases} L_{+} = \bar{y} \cdot (1 - f(\hat{y}))^{\gamma+} \cdot log(f(\hat{y})) \\ L_{-} = (1 - \bar{y}) \cdot p(f(\hat{y}))^{\gamma-} \cdot log(1 - p(f(\hat{y}))) \end{cases}$$

$$\text{where } f(\hat{y_i}) = \frac{1}{1 + e^{-\hat{y_i}}} \text{ and where } p(\hat{y}) = max(p - m, 0)$$

In order to account for sampling biases present in citizen science data, (**SM 1.7**), we also developed a new sampling-aware loss function (sampling-aware binary cross-entropy) which for each training example simply weights the loss contribution of present versus absent classes by the number of present and absent classes, respectively. The effect is that the magnitude of the contribution to the loss of present labels versus absent labels is approximately equal; in other words the correctness of the predictions for present species matters as much as the correctness of the predictions for absent species when calculating the model's regret.

$$\text{Sampling-aware BCE} = -L_{+} - L_{-} \begin{cases} L_{+} = \frac{\bar{y} \cdot log(f(\hat{y}))}{\sum_1^S \bar{y_i}} \\ L_{-} = \frac{(1-\bar{y}) \cdot log(1-f(\hat{y}))}{\sum_1^S 1-\bar{y_i}} \end{cases}$$

$$\text{where} f(\hat{y_i}) = \frac{1}{1 + e^{-\hat{y_i}}}$$

We compared the performance of each of these losses on the uniform test set (**SM 1.3.1**) using the remote sensing image-only *TResNet* architecture (**SM 3.2.1, Table S7**). All losses were implemented in *PyTorch* and can be found in the code repository associated with the project. We found our new sampling-aware loss function—which equally weights the training signal from present and absent classes—had competitive performance compared to these common functions while also exhibiting useful properties for downstream species mapping.

SM 3.2.3 *Deepbiosphere*, a novel climate + remote sensing CNN architecture

We were interested in testing a neural network architecture that could combine both remote sensing and climate sources to improve species modeling using CNNs. Previous work has shown that regional patterns in climate data can be interpreted by CNNs (45) but unfortunately climate rasters are too low resolution to be able to combine them with remote sensing images, as each 256 x 256 m remote sensing image will be assigned a single climate value (**Fig. S1**).

To get around this issue of scale, we created our own custom CNN model which combines a *TResNet* CNN head trained using NAIP imagery (**Table S2)** with a multilayer perceptron (*MLP*) head trained using climate inputs (**Table S5**), and we refer to this model as *Deepbiosphere* (**Table S3, Fig. 1C**). The *TResNet* head processes the high-resolution NAIP remote sensing image data (**Fig. S2B**) using 2D convolutions while the low-resolution bioclimatic information is processed pointwise by the *MLP* head (**Fig. S2C**) and ensembles the predictions through a series of fully-connected layers before predicting species, genus, and family like the *TResNet* architecture (**Table S3, Fig. 1C**). Overall, this architecture combines remote sensing imagery, climate data, taxonomic signal, and species co-occurrence patterns to make its species-level presence predictions. We found that *Deepbiosphere* performed better than *TResNet* models trained with just remote sensing imagery or *MLPs* trained with just climate variables (**Table S8**).

All model weights were initialized following best practices laid out in the original *TResNet* paper, using Kaiming He-style for CNN layers and zeroed out BatchNorm and residual connections (38). For all analyses, *Deepbiosphere's* outputs were converted to independent probabilities using the sigmoid transformation. Like the modified *TResNet*, we performed a linear learning rate optimization sweep for *Deepbiosphere*, testing a series of static learning rates from 0.01 to $1x10^{-6}$ in increments of 0.5, and found comparable performance across a range of learning rates ranging from $5x10^{-6}$ to $5x10^{-4}$. For model-to-model comparison, we trained *Deepbiosphere* with a static $1x10^{-5}$ learning rate—the same learning rate as the *TResNet* architectures—and for the case study analyses and change detection experiments, we used models trained with a static learning rate of $1x10^{-4}$.

SM 3.2.4 *Inception V3* Baseline

For comparison to previous work using CNNs to predict the presence of individual species from remote sensing imagery (46), we also trained an *Inception V3* architecture (*Inception,* **Table S4**) with softmax cross-entropy loss. This architecture only uses remote sensing imagery and the individual species labels associated with each remote sensing image and does not make use of climate data, taxonomic signal, nor species co-occurrence patterns in its modeling process. We used the official architecture implementation and weight initialization from *PyTorch*, using both the standard and auxiliary loss during training. We utilized the standard dropout rate of 0.5 and performed a learning rate optimization sweep for *Inception's* softmax cross-entropy-based loss with the more computationally-efficient *TResNet* architecture, and ultimately settled on an optimal static learning rate of $1x10^{-4}$ for the *Inception*-based model. While ref. (46) used a learning rate scheduler, we did not implement a scheduler in our training framework, so we only report accuracies for the model trained with the constant learning rate of $1x10^{-4}$. We also use the auxiliary loss for model training, which ref. (46) did not, and this addition should improve accuracy by preventing vanishing gradients. While the hyperparameters for training were slightly different from ref. (46), the *Inception V3* models trained with our hyperparameters exhibited expected behavior during training (monotonically decreasing training loss and increasing test set accuracy as training progressed), and thus should provide a fair comparison to previous work.

For all analyses, the *Inception* outputs were converted to a probability density function using the softmax transformation as in ref. (46). While the *Inception* model is trained jointly across all species like *Deepbiosphere*, the softmax cross-entropy loss forces the *Inception* CNN to fit a probability density function across species, meaning that it has been trained to predict just one species at a time and making it infeasible to use the model as a joint SDM effectively (see **Fig. S11B)**. Specifically, when using softmax cross-entropy loss, the raw model outputs can span from $-\infty$ to $+\infty$, so to map these predictions to probabilities the softmax transformation restricts that all probabilities across classes must sum to 1. This means that per-image, the probability of classes are dependent on one another (in this case, classes are species), and if a class is to rise in probability, another class must fall, making the probabilities across classes no longer independent. This lack of independence across species classes means that the predicted probabilities are not comparable across images and that these models are unable to produce probabilistically consistent maps of species' distributions across space, making them a poor choice for building species distribution maps. This means that if the *Inception* model trained with this loss predicts with high probability that a given species is present, for it to also predict another species as likely present, it must balance the probability assigned to the two species in a mutually-exclusive way. Naturally mutual exclusion proves problematic when wanting to predict upwards of hundreds of species simultaneously. Further, converting these probabilities to binary thresholds for calculating accuracy metrics becomes tricky. To this note, we still train and compare against the *Inception*-based model used in this work, and report accuracies for probabilities calculated using the *softmax* transformation, meaning that the *Inception* model will naturally have very low predicted probabilities per-species and per-image, and thus all binary classification metrics are 0 for this model.

Since the *Inception V3* model (**Table S4**) is significantly larger than the *TResNet*-based models (**Tables S2, S3**) and requires upwards of 5x the training time and compute, the *Inception* baseline CNN model was not included in the spatial cross-validation analyses as it was prohibitively resource-intensive to train and had poor results on the uniform split of the dataset for all binary accuracy metrics (**Table S8**).

SM 3.3 Climate-only Species Distribution Model baselines

Dozens of different SDM methods have been proposed over the decades, ranging from simple linear regression to neural network models. We chose to focus on the popular maximum entropy (*Maxent*) method and *Random Forest* method, as these two models performed consistently well compared to the dozens of approaches tried in ref. (47) across hundreds of species. Specifically, we use the popular *dismo* package for species distribution modeling (48) and compare against *Maxent* and downsampled single stacked *Random Forest* using best practices lined out in ref. (49). We also attempted to compare against ensembling approaches and run the popular *biomod* ensembling algorithm, but the algorithm was too slow and memory-intensive for us to be able to run it on all 2,221 species in our dataset.

We used *WorldClim* 2.0 bioclimatic variables (11) normalized to mean 0 standard deviation 1 (see **SM 1.5** for details). Following best practices from ref. (49), we removed all but one Bioclim variable with a Pearson correlation coefficient higher than 0.8, leaving ten variables in total for modeling including Mean Diurnal Range, Max Temperature of Warmest Month, Minimum Temperature of Coldest Month, Annual Precipitation, Precipitation of Wettest Month, Precipitation of Driest Month, Precipitation Seasonality, Precipitation of Wettest Quarter, Precipitation of Warmest Quarter, and Precipitation of Coldest Quarter.

For each species, we generated 50,000 background samples using a circular overlay across all points in the training dataset where the radius of each circle is the median distance between said species' observations. For the spatial cross-validation experiments (**SM 1.3.2**) we removed all background samples within the spatially withheld portion of the state. Finally, we used the same presence and background points for both the *Random Forest* and *Maxent* models.

SM 3.3.1 Maximum Entropy Baseline

For *Maxent*, we use a stacked single SDM approach to generate predictions for the 2,221 species in our dataset by generating individual models for each species then aggregating the predictions *post-hoc*. We use the *Maxent* implementation from the R package *dismo (48)* using the aforementioned background samples and all presences in a given train split of the main dataset. Consistent with ref. (49), we included the 'nothreshold' option and set the rest of the hyperparameters using *dismo* defaults. Although studies exist that run *Maxent* on the genus level, we nevertheless opted to only run *Maxent* to model species distribution, not genus or family. *Maxent* failed to run on 83 species, so for downstream accuracy analyses we imputed a prediction of 0.0 for these species.

SM 3.3.2 Random Forest Baseline

For the Random Forest baseline, we also take a stacked single SDM approach and use *dismo (48)*. For each species, we fit *Random Forest* with 1,000 trees using equal bootstrapping of positive and negative samples with replacement as outlined in ref. (49) with all other options set to the *dismo* default settings. 66 species did not properly fit for *Random Forest*, and so we imputed an accuracy of prediction of 0.0 for these species in subsequent analyses.

SM 3.3.3 Climate-only Multilayer Perceptron baseline

To compare the difference in remote sensing data versus standard bioclimatic data for species distribution modeling, we also considered how well a standard fully-connected multilayer perceptron (MLP) trained

using bioclimatic variables would perform as an SDM. MLPs trained on environmental data are a standard choice for SDMs, although *Maxent* and *Random Forest* have been more popular in recent years as they're less prone to overfitting (47).

We implemented a four layer, fully-connected MLP with BatchNorm in *PyTorch* inspired by ref. (50) which we refer to as the *Bioclim MLP*. This architecture consists of two fully-connected layers with 1,000 neurons each, followed by a dropout layer with a 0.25 dropout rate, then by two layers with 2,000 neurons each, before predicting species, genus, and family like the modified *TResNet* and *Deepbiosphere* architectures (**Table S5**). It should be noted that this MLP is *not* convolutional and learns from the raw value of environmental variables, rather than two-dimensional patterns of remote sensing data, like the CNN-based models.

For all experiments, we trained the *Bioclim MLPs* with sampling-aware binary cross-entropy loss (**SM 3.2.2**) and utilized co-occurring species information similar to the modified *TResNet* and *Deepbiosphere* architectures. All versions of this model were trained with a batch size of 1,000, as the model is significantly smaller than the CNN models and so a much larger batch size still easily fits on a small GPU. The optimal model learning rate was found using a stepwise sweep ranging from $5x10^{-6}$ to $1x10^{-1}$ in increments of 0.5 on the uniform test dataset, with the optimal learning rate being $1x10^{-5}$, similar to the *TResNet*-based architectures. The *Bioclim MLP* was trained with standard mini-batch stochastic gradient descent using the Adam optimizer for 100 epochs and like the other deep learning-based SDMs, the epoch of evaluation was determined using early stopping calculated from the average area under the receiver operator characteristic curve (AUC_{ROC}) on the uniform test set split (see **SM 2.2** for metric details).

SM 3.3.4 Trivial baselines

Finally, we also compared performance against two trivial baselines. The random baseline was calculated by drawing random values from a standard normal distribution five times and averaging the accuracy metrics across these five trials. The frequency baseline involved calculating the frequency of observations per-species on the training set, rescaling the frequencies to 0.001-1.0 and imputing these frequencies as the predicted probabilities at each test set example.

SM 4. Individual species case studies

While the ability to successfully predict the presence of thousands of species at once is in itself an impressive feat, also of importance is *how* such an SDM can be useful for a variety of downstream ecological tasks. By modeling each individual species of a community, we can begin to detect not just individual species, but ideally patterns of the entire community as well. To begin to explore the power of a multi-species modeling approach, here we present two case studies of well-known vegetation communities and their species and demonstrate how *Deepbiosphere* can detect expected species-level range dynamics across a variety of ecoregions.

SM 4.1 Validating *Deepbiosphere's* predictions for individual species

Since *Deepbiosphere* generates predictions for thousands of plant species simultaneously, it can theoretically be used to detect and model both individual species along with the broader vegetation community. For the high-resolution case studies to compare to human annotators, we focused on two large charismatic tree species—redwoods (*Sequoia sempervirens*) and valley oak (*Quercus lobata*. We specifically chose these two species as they are observable directly from the NAIP imagery so that human annotators could distinguish their specific canopies and so that human annotations could be reasonably assumed to represent some proxy of ground-truth presence and absence. For both case studies, locations

were chosen using expert knowledge of the respective species ranges and known occurrences from Calflora (64) (**Table S11**).

It is important to note that for the human annotator comparison case studies, the versions of *Deepbiosphere, Maxent, Random Forest*, and *Bioclim MLP* used in these case studies were trained without observations or pseudoabsences from the respective spatial cross-validation band where the case study was located (see darkened band inside California inset in **Figs. 2, S14**). Thus, these models did not see any example images or climate variables from the respective regions at train time, with the nearest training examples located between 9–20 km away from the case studies (**Fig. S4B**). Conversely, the *Inception V3* baseline was trained using the uniform split of the dataset and thus was trained with multiple observations from within the case study areas (**Fig. 4A**, **S11B**).

For the additional state-wide species range maps (**Figs. S22-23**), 55 species representing a mix of well-predicted and randomly-selected species were chosen. Well-predicted species (**Fig. S22**) were chosen by assigning species to one of the five L2 ecoregions of California based on which L2 ecoregion the most *Deepbiosphere* uniform training set observations fell into, then filtering these species to only include those with at least 10 *Deepbiosphere* uniform test set observations and species with a test set AUC_{ROC} accuracy of at least 0.98 for either *Deepbiosphere* or *Maxent* (for a total of 239 possible species). Then, for both *Deepbiosphere* and *Maxent*, the top-5 most well-predicted species per-L2 ecoregion were chosen, for upwards of a total of 10 species per-L2 ecoregion. Not every ecoregion has 10 species plotted if there was overlap between the top-5 best AUC_{ROC} species for *Deepbiosphere* and *Maxent*, or if one of the two models did not have a high enough AUC_{ROC} on well-supported species. For the randomly-chosen species (**Fig. S23**), species with a *Deepbiosphere* test set threshold of at least 10 observations (leaving 1,007 possible species) were assigned to one of the five L2 ecoregions of California based on which L2 ecoregion the most *Deepbiosphere* uniform training set observations fell into, and for each L2 ecoregion, five species were randomly selected from the list of species most common to that ecoregion using the numpy.choice function and a random seed of 1.

To quantify these 55 species, occurrence records for each species were obtained from the independently-sampled Calflora occurrence record database (64). For each of the 55 species, all occurrences from Calflora, the Consortium of California Herbaria, and the Consortium of North American Bryophyte Herbaria were downloaded, only excluding *iNaturalist* observations as those records are likely present in the *Deepbiosphere* training dataset. From these occurrences, those present within California that included location information were used, including obscured records, all varieties, and sub-species. To quantify *Deepbiosphere* and *Maxent's* predictive accuracies using AUC_{ROC} from these records, each known species location was considered a presence and absences were derived from the location of all other Calflora occurrences for the selected species in **Figs. S22** and **S23** not predominantly found in the species' ecoregion (e.g., excluding observations for all other species predominant to the Warm Deserts L2 ecoregion for *Bahiopsis parishii*). Observations from species common to the L2 ecoregion were excluded as absence points as these species may co-occur, thus representing pseudo-absence points as opposed to true absence points. In total, 30,543 observations were curated from Calflora to validate the models' performances.

Additionaly, from each L2 ecoregion one species was chosen for a high-resolution case study zoom-in at ~1, 0.1, and 0.001 degrees resolution (**Fig. S24-28**). Species were chosen to highlight the various strengths of using remote sensing data for species range mapping, including the ability to detect differences in soil type (**Figs. S25, S27**), land use (**Fig. S26**), and geographically-isolated extant populations (**Figs. S24, S28**).

SM 4.2 Generating high-resolution species range maps with CNN SDMs

Deepbiosphere, like all classification-based CNN models, takes in images of ideally a set dimensionality (for our work, 256 x 256 pixels) and makes a single prediction for this image (in our formulation, a single

prediction consists of a predicted presence of 2,221 plant species). In other words, *Deepbiosphere* can produce a prediction of all 2,221 species for any arbitrary 256 x 256-pixel image. In order to get a map of predictions at a set ground-level resolution using this model, one simply has to convolve this local receptive field every K pixels to generate a map of K-pixel resolution, a technique similar to the striding operation in the convolutional layers of a CNN. Then, depending on the resolution of the imagery used to make the predictions, one can determine the ground-level resolution of the predictions themselves. For example, to generate a 50 m ground-level resolution map from 1m NAIP imagery, a stride of 50 should be employed. Alternately, to generate a 30 m ground-level resolution map from 60 cm NAIP imagery, a stride of 50 will also suffice. A visual explanation of the striding procedure can be found in **Fig. S8**. Each of these strided blocks we refer to as *map pixels*, which can then be compared to other maps by aligning *Deepbiosphere's* map pixels to the other map's pixels through co-registration using the underlying spatial extent of the map pixels. *Deepbiosphere* is efficient enough to generate maps for all 2,221 species across the state of California at 150m resolution, taking about a day to run with 30-core parallelization (**Fig. S22, S23**).

SM 4.3 Human annotation protocols

To generate ground-truth human annotations at a similar resolution to *Deepbiosphere's*, a user study was implemented in Google Sheets where human annotators classified the same NAIP imagery as *Deepbiosphere* by percent cover. To calibrate annotators to the task, each annotator received three NAIP images from 2012 (the same imagery used to train *Deepbiosphere*) and an assigned cover classification using known species occurrences pulled from *Calflora* (64) (**Table S11, Fig. S9, S14C**). These example photos were chosen from outside the case study area yet within the species' core ranges and were annotated on a scale of 0% cover to 100% cover at five different levels.

Annotators were then given the full NAIP imagery (**Fig. S9, S14A**) partitioned into 256 × 256m blocks—which were labeled A-Z and 1-30 to correspond with the appropriate cell in Google Sheets—and were asked to label each corresponding image block in its matching cell. Annotators were not domain experts and the only training received *a priori* were the three already classified example images (**Figs. S9, S14C**). Three non-expert human annotators annotated *Sequoia sempervirens* cover and two annotated *Quercus lobata* cover. Annotations took between 30 minutes to two hours per-case study (depending on the efficiency and familiarity of the annotators with the task) and final cover scores were calculated by averaging annotations per-pixel across annotators.

SM 4.4 Redwoods case study

To validate the predicted presence of redwoods across vegetation types and models, the National Park Service's (NPS) 2017 vegetation mapping and classification project was used, specifically the alliance-level classification mapped to the thirty vegetation classes used for the accuracy assessment in ref. (51). To generate this map, the association-level vegetation map was cross-walked to the generalized alliance level (see section 2 of ref. (51) for details on class type designations). We further grouped these alliance-level classes into the thirty vegetation classes used in ref. (51)'s accuracy assessment (see 6.2.1 of ref. (51) for details on the map classes; see sections 5 and 6 of ref. (51) for justification on classes that were removed) and filtered the mapped vegetation categories to only include those mapped to these thirty classes. For the final map, the class "mature redwoods" mapped to the *Sequoia sempervirens* Mature Forest alliance, the class "young redwoods" mapped to the *Sequoia sempervirens*- (other) YG alliance, and the class "other vegetation" mapped to all other alliance-level classes present in the study area. Per-pixel labels were determined based on which alliance had the largest area overlap with the pixel's extent.

The co-occurring species used for the understory analysis were chosen based on importance values for primary versus secondary-growth redwoods as reported in Table 2 of ref. (52) from 16 inventory plots and

the constancy values generated from 65 relevé surveys reported in ref. (51) for Manual of California (MoC) vegetation associations crosswalked to either the *Sequoia sempervirens*-(Other) YG Mixed Forest alliance (*Lithocarpus densiflorus-Sequoia sempervirens* Young Growth association [Appendix B Table B10], *Alnus rubra-Sequoia sempervirens* Young Growth association [Appendix B Table B8], and *Pseudotsuga menziesii-Lithocarpus densiflorus-Sequoia sempervirens* Young Growth association [Appendix B Table B11]) or the *Sequoia sempervirens* Mature Forest alliance (*Sequoia sempervirens- Acer macrophyllum-Umbellularia californica* association [Appendix B Table B12], and *Sequoia sempervirens/Vaccinium ovatum/Polystichum munitum* association [Appendix B Table B14]).

Species were first filtered to only include shrubby and herbaceous understory species present in Table 2 of ref. (52) and those that were present in all constancy tables for the crosswalked MoC associations. Species were further filtered to only include species who showed similar association patterns across the two independent studies (e.g. higher constancy values on average for old-growth-associated plots in both studies and lower constancy values on average for secondary-growth- associated plots in both studies). Specifically, species were considered to be old-growth-associated if they had a higher importance value for old-growth versus secondary-growth in ref. (52) and a strictly higher constancy in old-growth-associated MoC vegetation associations compared to secondary-growth-associated MoC associations in ref. (51). Species were considered to be secondary-growth-associated if they a higher importance value for secondary-growth versus old-growth in ref. (52) and a strictly higher constancy in secondary-growth-associated MoC vegetation associations compared to old-growth-associated MoC associations in ref. (51). Species were considered to be associated with both forest types if the importance values for old-growth versus secondary-growth plots in ref. (52) were smaller than 3.0 and the average difference between constancy values in in ref. (51) was < 10. Only 6 species met this filter, two that were secondary-growth-associated, two that were old-growth-associated, and two that were associated with both types of vegetation. Constancy values are individually reported for each crosswalked MoC association and also for the redwood forest maturity type reported in the relative frequency column of Table 2 in ref. (52), ultimately leaving four independently sampled constancy values for the *Sequoia sempervirens*-(Other) YG Mixed Forest class and three independently sampled constancy values for the *Sequoia sempervirens* Mature Forest class.

SM 4.5 Oaks case study

For the oaks case study, the United States Department of Agriculture (USDA) Forest Service's Region 5 South Coast existing vegetation map (53) was used to validate species predictions, specifically the type 1 regional dominance (REGIONAL_DOMINANCE_TYPE) crosswalked to species using the Classification and Assessment with Landsat of Visible Ecological Groupings (CALVEG) class descriptions from Region 5's Zone 7 (54). A species was considered present within a given regional dominance type if said species' name was mentioned in the corresponding CALVEG Zone 7 vegetation description. The final species to CALVEG mappings are as follows: *Ceanothus cuneatus*: CC, CQ, EX; *Quercus lobata*: QL; *Bromus diandrus*: HG; *Quercus berberidifolia*: CQ; *Arctostaphylos glandulosa*: CQ, SD, *Adenostoma fasciculatum*: QA, CC, CQ, SS, EX. Other CALVEG classes were present in the study area, but either were associated with developed and agricultural land use or were too small in area to map to a sufficient number of pixels and were thus excluded from the analyses. For comparing predicted presence inside versus outside CALVEG zones, CNN-based SDM predictions made at 256 m resolution were used to minimize spatial autocorrelation. For a given species, pixels were marked as "inside" if a given pixel intersected at least one of the associated CALVEG classes for that species and was marked as 'outside' otherwise.

SM 5. Mapping spatiotemporal changes with *Deepbiosphere*

On top of modeling individual species, *Deepbiosphere's* multi-species predictions can also be evaluated in aggregate to explore more macroecological trends in community composition, such as spatial and temporal

change. Historically, these processes have been difficult to observe directly without detailed species checklist data, limiting the resolution at which these metrics can be generated (55, 56). However, combined SDMs for thousands of species have been proposed previously to measure certain important ecological phenomena, such as the imperiled species index (27). Here, we showcase that *Deepbiosphere* could potentially be used for similar purposes, especially for detecting community change in space and time at high-resolution.

SM 5.1 Detecting spatial community change

In order to interpret *Deepbiosphere's* predictions of species presence as community change, a novel edge detection algorithm inspired by common edge detection filters from computer vision was used (see **Fig. S19** for visual walk-through). Specifically, the averaged one-neighbor Euclidean norm was calculated per-pixel from a map of all 2,221 species predictions to generate a map of averaged similarity to neighbor pixels (See **SM 4.2** for map generation details). This algorithm essentially measures the average distance of the predictions of a given pixel to all its nearest neighbor pixels, summarizing how similar or different a given pixel's predicted species list is from nearby areas. Another interpretation of this statistic is as a measure of the average rate of change of species composition within a local area.

The Euclidean norm was the chosen statistic as it is a metric that encodes both magnitude and direction. Therefore, it captures both changes in a given species' presence across pixels through the directional component (e.g: a species highly predicted in one pixel has much lower predicted probability in the other pixel) and capture changes in the raw number of species predicted through the magnitude component (e.g.: many species are predicted with high probability in one pixel, but then very few are predicted with high probability in the other pixel). Generalizing, for all non-edge pixels in the given extent of *Deepbiosphere's* presence predictions, we take the Euclidean norm from the central pixel to its neighboring pixels, then take the average across its eight neighbors to generate the final spatial community change value. Below, $\hat{y}_c$ is the predicted probability vectors for all 2,221 species at the given pixel of interest and $\hat{y}_{1N}$ are the predicted probability vectors at all eight one-neighbor pixels (see **Fig. S19** for visual walk-through).

$$\text{average one-neighbor Euclidean norm} = \frac{\sum_{i=1}^{8} \|\hat{y}_c - \hat{y}_{1i}\|}{8}$$

We refer to this averaged one-neighbor Euclidean norm metric as *spatial community change*. Directly validating spatial community change is exceedingly difficult, as such data rarely exists at scale. As a proxy, the number of unique alliance-level vegetation classes in a given pixel is used to approximate how rapidly habitat transitions are occurring in a given location. The rationale is that more vegetation classes intersecting in the pixel means that the area is likely an ecotone, and thus should have a higher spatial turnover of species.

To validate *Deepbiosphere's* spatial community change predictions, we utilized a case study associated with the 2018 Marin fine-scale vegetation map (**Fig. S17C**) (57), calculating the number of vegetation classes intersecting each pixel. The number of intersecting alliance-level vegetation classes were counted per 256 x 256 image using Geopandas' "intersects" function (58) and the spatial community change metric was correlated to the number of vegetation classes using the modified *t*-test from SpatialPack using the centroid of each pixel as the coordinates per-sample (59).

To confirm that predicting changes in community composition is more complex than simply predicting the change in greeness or infrared absorption, a similar comparison to the number of intersecting vegetation classes was performed using the averaged one-neighbor Euclidean norm between the normalized raw NAIP pixel values per-band, upsampled to 256 m resolution. (**Fig. S17D**). Since *Deepbiosphere* was trained with observations from the case study region (**Fig. S17E**), to confirm that predicting changes in community

composition is also not as simple as simply recapitulating the number of species in a given area, the number of unique species observed in a given pixel was also correlated with the spatial community change from *Deepbiosphere* (**Fig. S17F**).

SM 5.2 Detecting temporal community change

One major benefit to using remote sensing data to train SDMs as compared to climatological datasets like WorldClim is that rapid, short-term ecological change is more readily captured in high-resolution remote sensing imagery as opposed to long-term climatological trends (**Fig. S1D-F**). To test the utility of high-resolution remote sensing imagery for capturing changes in time, we conducted a case study from the Rim Fire that occurred in the Sierra Mountain foothills. At the time of the blaze, the Rim Fire was California's second-largest fire on record. More specifically, the blaze occurred in the Hetch Hetchy Valley in the western Sierra mountains, and occurred in the fall of 2013, giving an entire growing season between the fire and the acquisition of the next round of NAIP imagery in summer of 2014. In order to detect temporal change, a metric similar to the spatial community change was developed. Specifically, the per-pixel Euclidean distance between *Deepbiosphere's* predicted species probabilities made from NAIP imagery acquired in 2012 and 2014 was used to approximate the magnitude of temporal community change.

$$\text{temporal Euclidean distance} = \|\hat{y}_{2012} - \hat{y}_{2014}\|$$

This temporal Euclidean distance metric we refer to as the *temporal community change* (visual explanation found in **Fig. S21**). This change metric essentially measures the magnitude of per-species change (including both increases and decreases) aggregated between the two timepoints.

As with spatial community change, directly detecting temporal community change is difficult. However, hyperspectral data was independently collected within the bounds of the fire at high resolution, enabling the estimation of the differenced normalized burn ratio (dNBR), a popular metric of fire severity (60). Normalized burn ratio (NBR) is an empirical measurement of burn severity calculated by taking the difference between infrared wavelengths—which capture photosynthesis intensity—and shortwave infrared wavelengths—which capture heat absorption from char—and is typically measured using hyperspectral spectrometers such as AVIRIS or MASTER sensors (61). To calculate dNBR, the change in NBR from data acquired before the fire to data acquired after is calculated. For this analysis, MASTER sensor data was chosen as it had more coverage over the fire than the AVIRIS sensor data, covering roughly half of the fire's extent. Specifically, dNBR calculated using NBR acquired in June of 2014 was used for validation, as that was the closest time point to the acquisition of the corresponding NAIP imagery. In order to reach the same resolution as the empirical burn severity data, we used a 35 pixel stride (**SM 4.2**).

Deepbiosphere's predicted temporal community change metric was correlated to dNBR using Pearson's *r* corrected for spatial autocorrelation using the Dutilleul correction from the SpatialPack R package using the centroid of each pixel as the sample coordinates (59, 62). Before correlation, both dNBR and *Deepbiosphere's* temporal community change predictions were upsampled to 256 m resolution as the spatial correction calculation is very computationally intensive. The Pearson's *r* does not change substantially between correlations calculated at 35 m versus 256 m resolution, thus only the 256 m results are reported.

As with spatial community change, the Euclidean distance between NAIP imagery acquired in 2012 and 2014 was calculated to confirm that detecting fire severity is not as simple as recapitulating the difference in the infrared and green bands across time. NAIP imagery was upscaled to 35 m and 256 m resolution respectively, normalized, and mean-centered in the same way that imagery is prepared during *Deepbiosphere* model training. The correlation between the distance in NAIP pixels and dNBR was also

generated at 35 m and 256 m resolution, and as with the temporal community change, the Pearson's *r* does not change substantially between correlations calculated at 35 m versus 256 m resolution.

Supplemental Figures

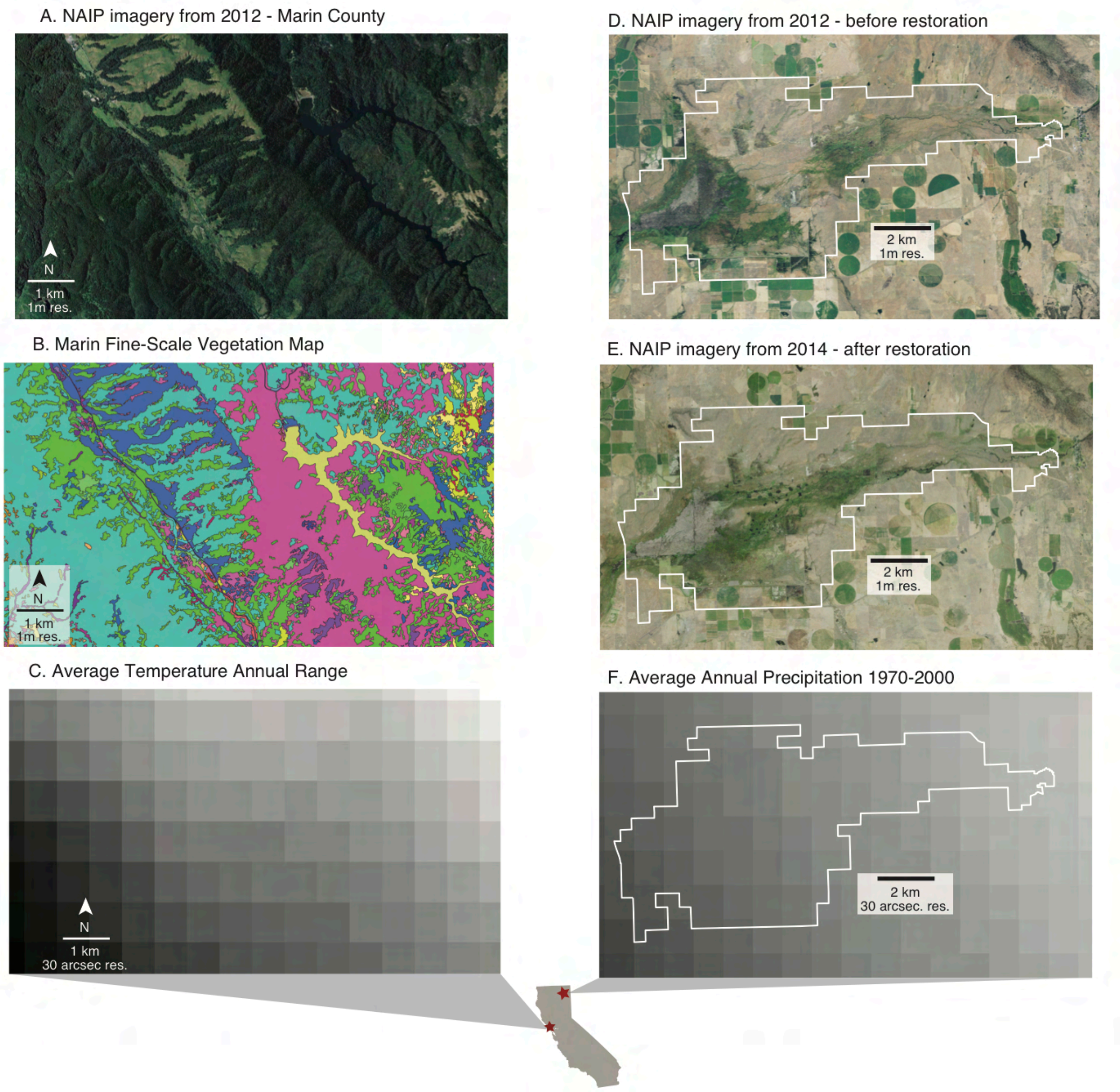

Fig. S1 | Spatial and temporal resolution comparison of remote sensing data vs. SDM bioclimatic covariates
(A) National Agricultural Imagery Program (NAIP) imagery data acquired in 2012 for northern Marin County in northwest California. From this imagery, different ecological features of this ecological transition zone are clearly visible, such as the light green of invasive annual grasslands interspersed with patches of coast live oak. **(B)** Fine-scale vegetation map from ref (57), which contains almost 50 highly fragmented unique habitats and land use categories for the study area. **(C)** Temperature annual range from WorldClim 2.0 (11), a version of bioclimatic variables commonly used as covariates for species distribution modeling (SDM). These bioclimatic variables are of low spatial resolution and do not capture the local habitat changes in (B) that are clearly visible from remote sensing imagery in (A). **(D)** National Agricultural Imagery Program (NAIP) imagery data acquired in 2012 at a degraded ephemeral freshwater wetland at Ash Creek Wildlife Area in northern California (park bounds outlined in white). The degradation of the wetlands is clearly visible as the large light brown grassland intrusion cutting through the middle of the much greener and darker-colored wetland (center of image) **(E)** NAIP imagery acquired in 2014, after a restoration project was undertaken to restore the degraded wetland through a series of pond and plug procedures (visible as small dark dots in the center of image). The restored wetlands are clearly visible as a now-continuous band of green vegetation traversing the majority of the wildlife area. **(F)** Annual precipitation from WorldClim 2.0 at Ash Creek Wildlife Area (11). The WorldClim bioclimatic variables are averaged across a three decade timespan (1970-2000) and thus fail to meaningfully capture this rapid temporal ecosystem change.

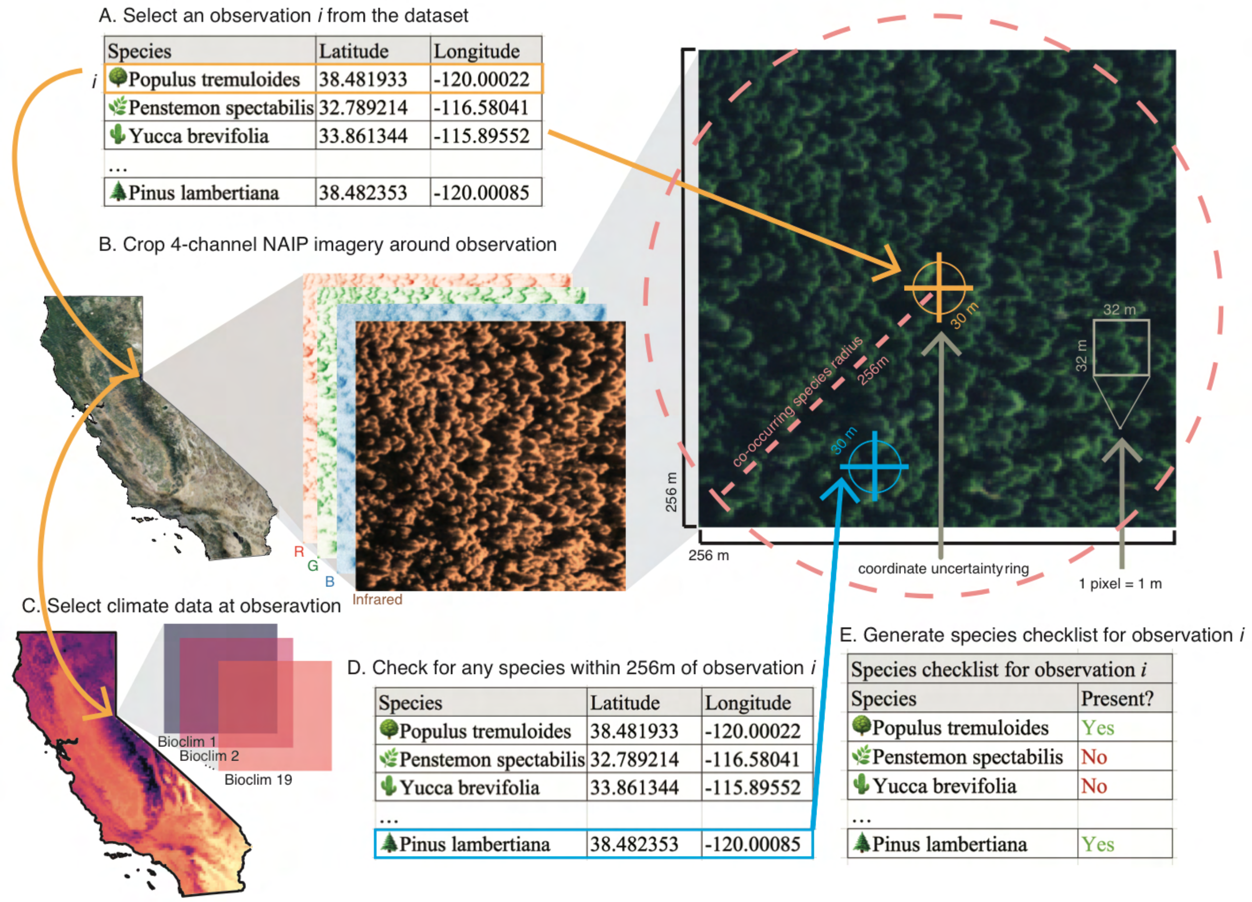

Fig. S2 | Illustration of building the biodiversity dataset
(A) For each unique observation in the dataset, first, a species observation is selected. **(B)** For this selected observation, a 256 × 256 pixel image is generated from the four band NAIP imagery, centered at the geographic location of the observation (orange cross). Observation locations may have upwards of a 30 m radius of geographic uncertainty (orange circle) but still fall well within the image extent. **(C)** Next, the bioclimatic variables for that location are selected. The resolution of the bioclimatic variables is much coarser than the remote sensing imagery, thus for each 256 x 256 pixel remote sensing image, only one pixel of bioclimatic data is selected. **(D)** Afterwards, a list of overlapping species is generated by selecting other observations from the dataset (blue cross) whose coordinates fall within a 256 m radius of the original observation (pink circle) **(E)** The final data products consist of the four-band remote sensing image (B), the Bioclim variables (C) and the partial species checklist (E).

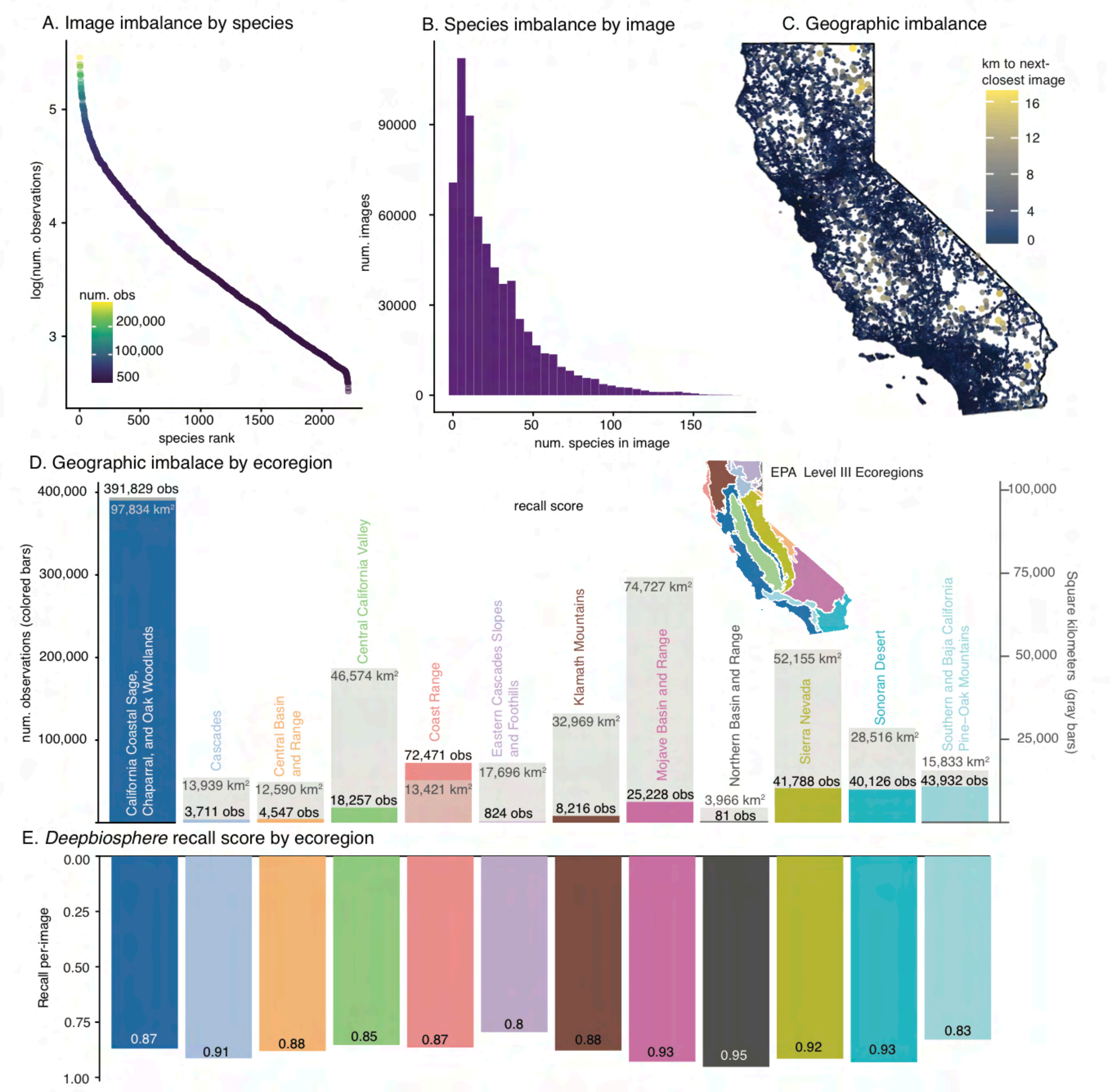

Fig. S3 | Biases present in the dataset and their effects on accuracy

(A) Many species have few images in the dataset, while a few species have a disproportionate number of observations. This extreme imbalance between species class frequencies can make it difficult for classic machine learning methods that rely on an assumption of an equal number of examples per-class to learn a good representation. **(B)** The dataset also exhibits significant imbalance in the number of species labeled per-image in the dataset. Most observations have few overlapping species in the observation—likely not fully describing all species actually present in a given site—while only a few observations contain many species and present a more accurate checklist of species presence in a given area. **(C)** Plot of distance to nearest non-overlapping observation across California. Color and size represent the distance in kilometers to the next-closest non-overlapping image in the dataset. As to be expected from opportunistic citizen science data, observations tend to cluster and distances are not distributed evenly. **(D)** Comparison of number of species occurrences in a given Level III EPA ecoregion versus the area of that ecoregion. Citizen science observations like those used in the dataset tend to cluster around population areas and natural regions where observers can reach. This leads to oversampling in some ecoregions, especially the California Coastal Sage, Chaparral and Oak Woodlands and Coast Range, and significant undersampling in other regions, especially the Cascades and Northern Basin and Range habitats. Colored bars represent the number of observations in the dataset from that region and the grey bars represent the area in square kilometers of the specific ecoregion. Inset is a map of the Level III ecoregions of California. **(E)** Average per-image recall accuracy of *Deepbiosphere* on the uniform split of the dataset (same model as **Table S8**). The accuracy differences of *Deepbiosphere's* predictions across ecoregions is less pronounced than the underlying citizen science data, implying that the model is learning patterns of species' distributions that are generalizable across regions.

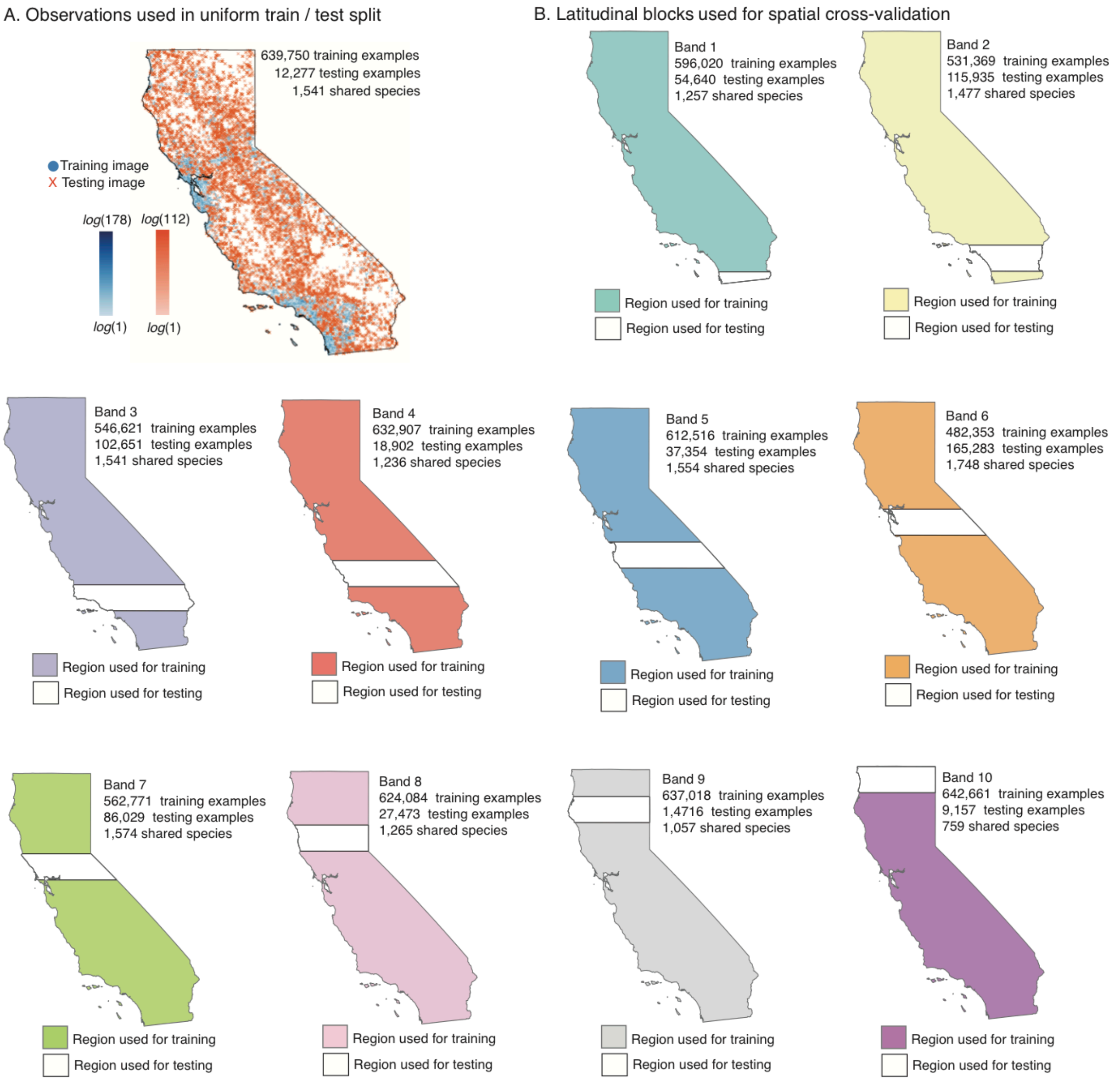

Fig. S4 | Partitioning the dataset for cross-validation

(A) Location of observations used for the uniform split of the dataset. Observations were selected for the test set if they were at least 1.2 km away from any observation in the training set to ensure that there was no train/test leakage for Bioclim-trained models. Color scales represent the number of unique species present in each observation, log-scaled. **(B)** Location of test blocks for spatial cross-validation. A 10-block spatial holdout procedure was employed to test models' extrapolation ability. Areas that were included in the training set are in color and areas used for the test set are in white. Any images within 1.3km of the test region were removed from the training set in order to prevent potential overlap between bioclimatic variables in the train and test splits (grey boundary). The number of images within each split is also annotated, along with the number of unique species present in both splits.

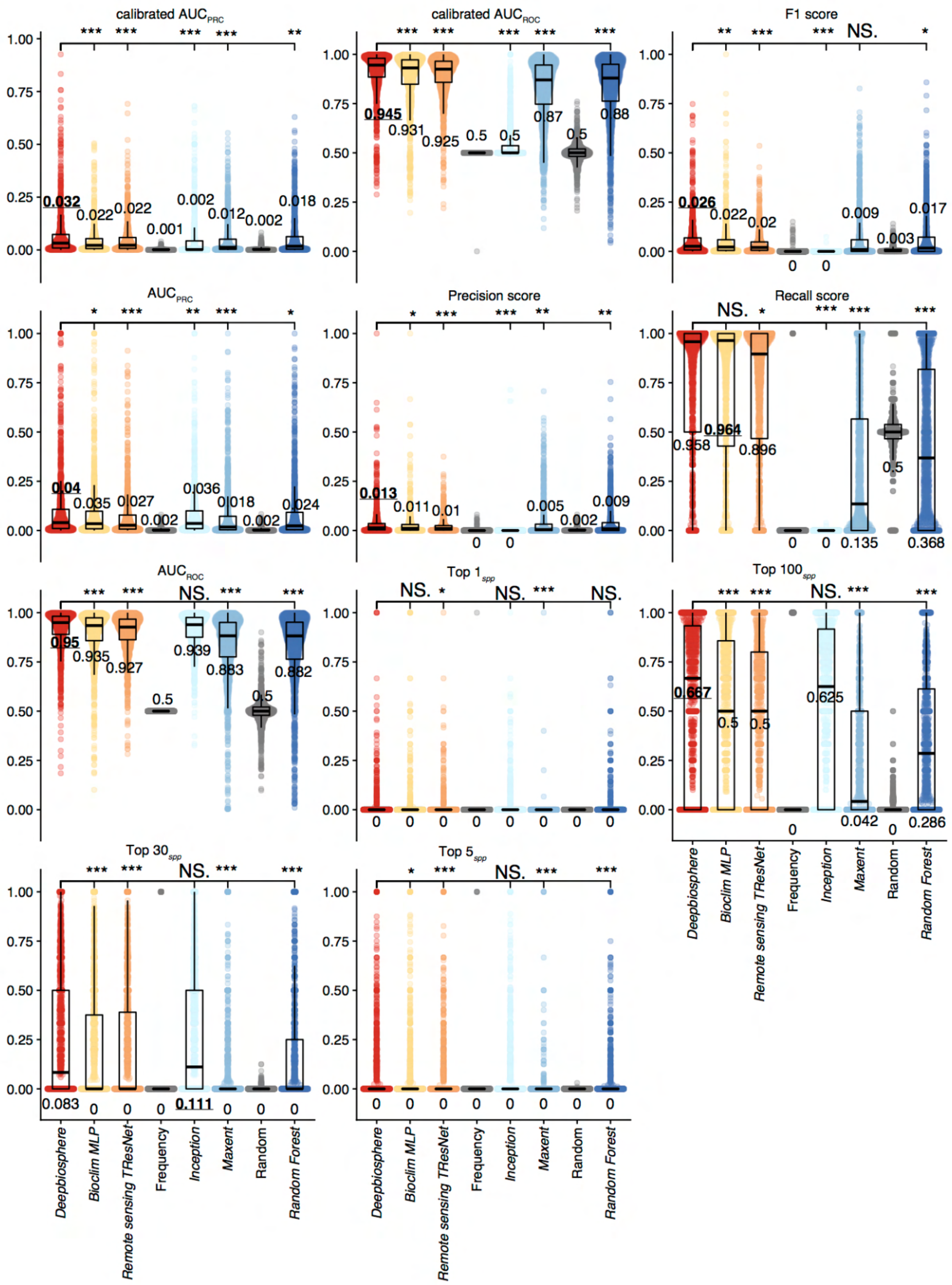

Fig. S5 | Comparison of SDM models' accuracy metrics across species

Here we report the accuracy of *Deepbiosphere* to baseline models on the uniform split of the dataset (**Fig. S4A**) for the eleven accuracy metrics that can be calculated per-species, using species found both in this train and test set split (1,541 species out of 2,221 total species in the dataset). On average, *Deepbiosphere* outperforms the baseline approaches, having a higher median accuracy for seven out of the eleven metrics reported here. Stars represent unpaired student *t*-test comparing *Deepbiosphere's* accuracy per-species to the relevant baseline SDMs with *** indicating a *P*-value of $< 10^{-3}$, ** indicating a *P*-value of $< 10^{-2}$, * indicating a *P*-value of $< 10^{-1}$, and NS. indicating a non-significant *P*-value. Annotated values are the median accuracy for that metric, bolded and underlined for the model with highest accuracy. Batch size, learning rate, and epoch of evaluation can be found in **Table S8**.

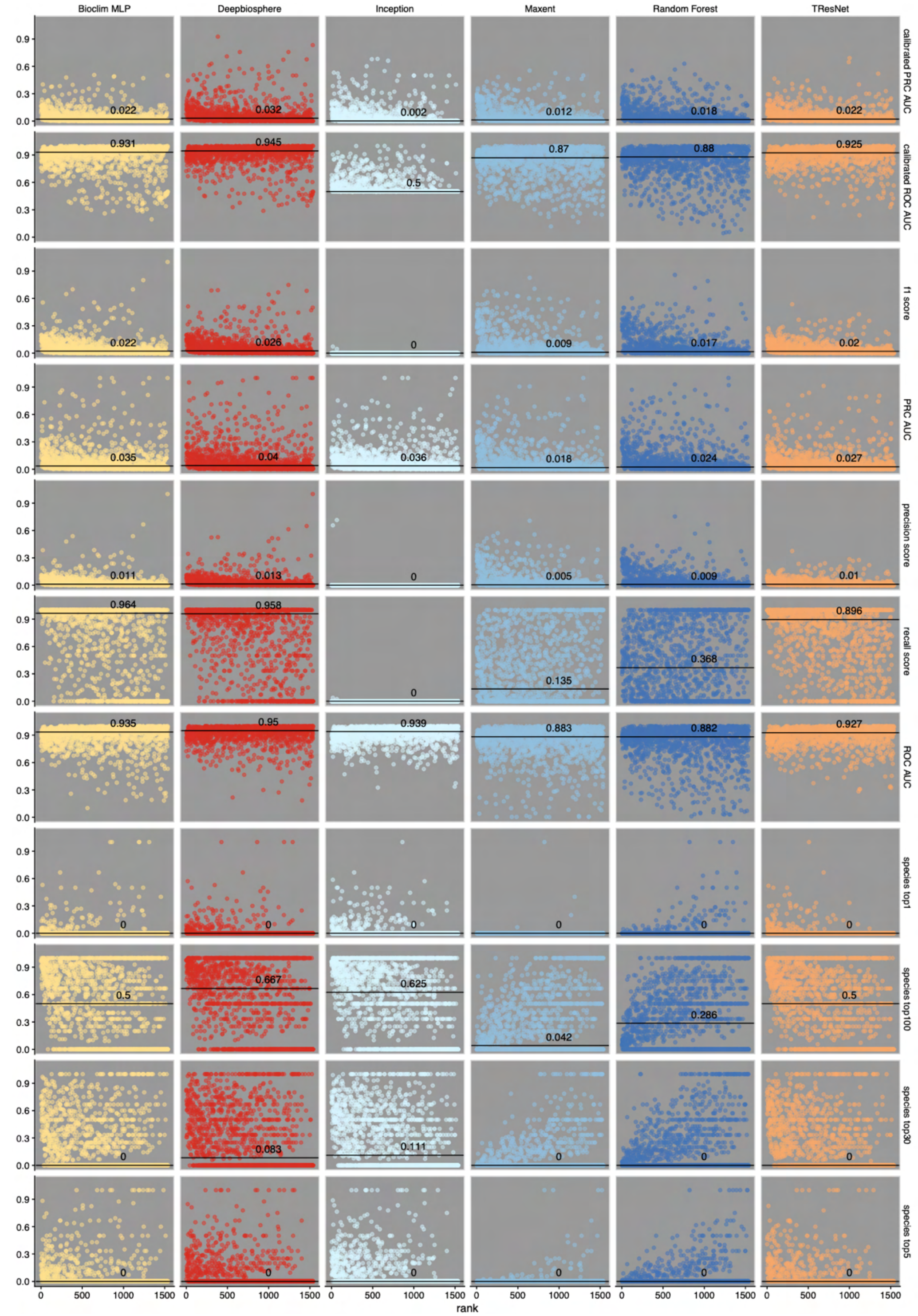

Fig. S6 | Comparison of SDM models' accuracy metrics by species rank in the dataset

Here we visualize all eleven accuracy metrics that can be calculated per-species (columns) for *Deepbiosphere*, its building block models, and all baselines (rows) by breaking down the per-species accuracy by how frequent said species is in the dataset (rank, X axis). In general, we see that performance degrades for all models as species become rarer, but not universally (see species top-K metrics). We also see that oftentimes *Deepbiosphere's* performance on rarer species degrades less severely than other modeling approaches (see precision, AUC_{ROC}), implying that modeling choices such as our sampling-aware loss function and use of co-occurrence data helps improve rare species performance. Species rank was calculated using the un-imputed observations across all train and test splits, with rank 0 corresponding to the most common species. Annotated lines correspond to the median accuracy for that model and metric.

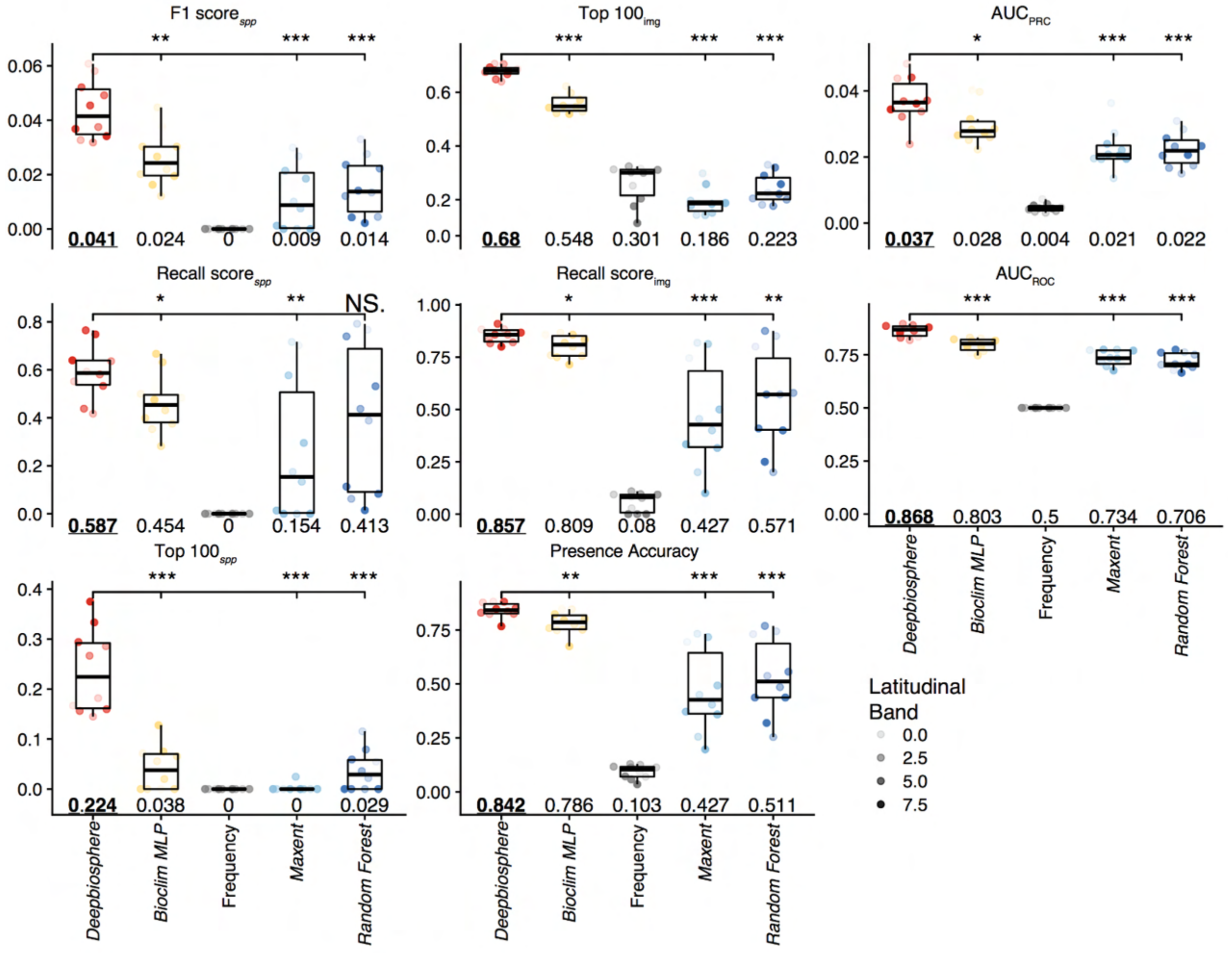

Fig. S7 | Comparison of SDM models' accuracy metrics across latitudinal cross-validation bands

Here we report the accuracy of *Deepbiosphere* to baseline models on the latitudinal cross-validation blocks (**Fig. S4B**) for the eight accuracy metrics from **Table 1**. *Deepbiosphere* outperforms all climate-based baseline approaches and has a higher median accuracy for the eight metrics reported here. Dots are median accuracies per-band. Stars represent unpaired student *t*-test comparing *Deepbiosphere's* accuracy per-band to the relevant baseline SDMs with *** indicating a *P*-value of $< 10^{-3}$, ** indicating a *P*-value of $< 10^{-2}$, * indicating a *P*-value of $< 10^{-1}$, and NS. indicating a non-significant *P*-value. Annotated values are the median accuracy for that metric, bolded and underlined for the model with highest accuracy. Batch size, learning rate, and epoch of evaluation can be found in **Table S10**.

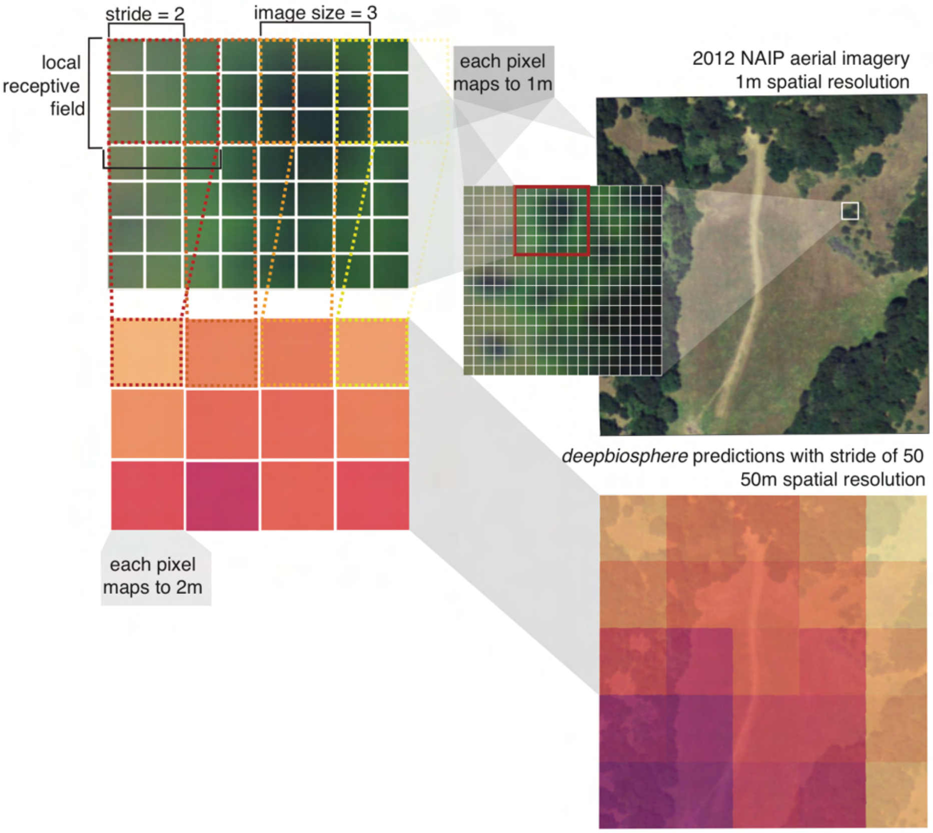

Fig. S8 | Generating high-resolution predictions with *Deepbiosphere*
To generate high resolution predictions from *Deepbiosphere*, a technique from computer vision called striding is employed. Since *Deepbiosphere* is trained with images of size 256 x 256 pixels, we are limited to generating predictions of this size and shape. However, we can get a higher resolution by shifting *Deepbiosphere's* local receptive field (the current 256 x 256 pixels used to make a prediction) by *K* pixels to generate a map of *K*-m resolution. This iterative sliding of the local receptive field is similar to striding, a technique used within the architecture of many CNN models. For example, to generate a 2 m-resolution map from 1 m resolution imagery using a CNN that makes predictions using a 3 x 3 image, we would generate predictions for each 3 x 3 image within the map, striding the predictions by 2, resulting in a final map with a ground resolution of 2 m. When comparing maps made using this approach with *Deepbiosphere* to other maps, each map pixel corresponds to the geographic area covered by each pixel; for example, each map pixel in the bottom right maps to a 50 x 50m block spatial extent and can be co-registered to data from other maps that overlap the same spatial extent.

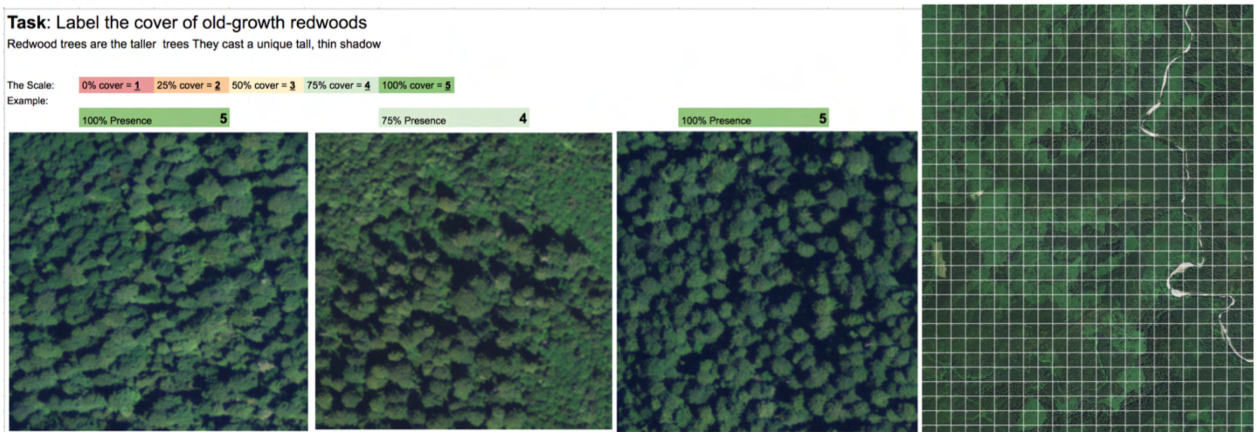

Fig. S9 | Example images from human redwood labeling task
(**A**) Labelers were given these three examples of old-growth redwoods as positive examples of redwood forest before completing the annotation task. Details of the three locations can be found in **Table S11**. (**B**) Block partitions of the imagery used by human annotators to generate redwood cover maps. Each block was annotated by 3 human annotators using the 1-5 scale in (**A**) as guides.

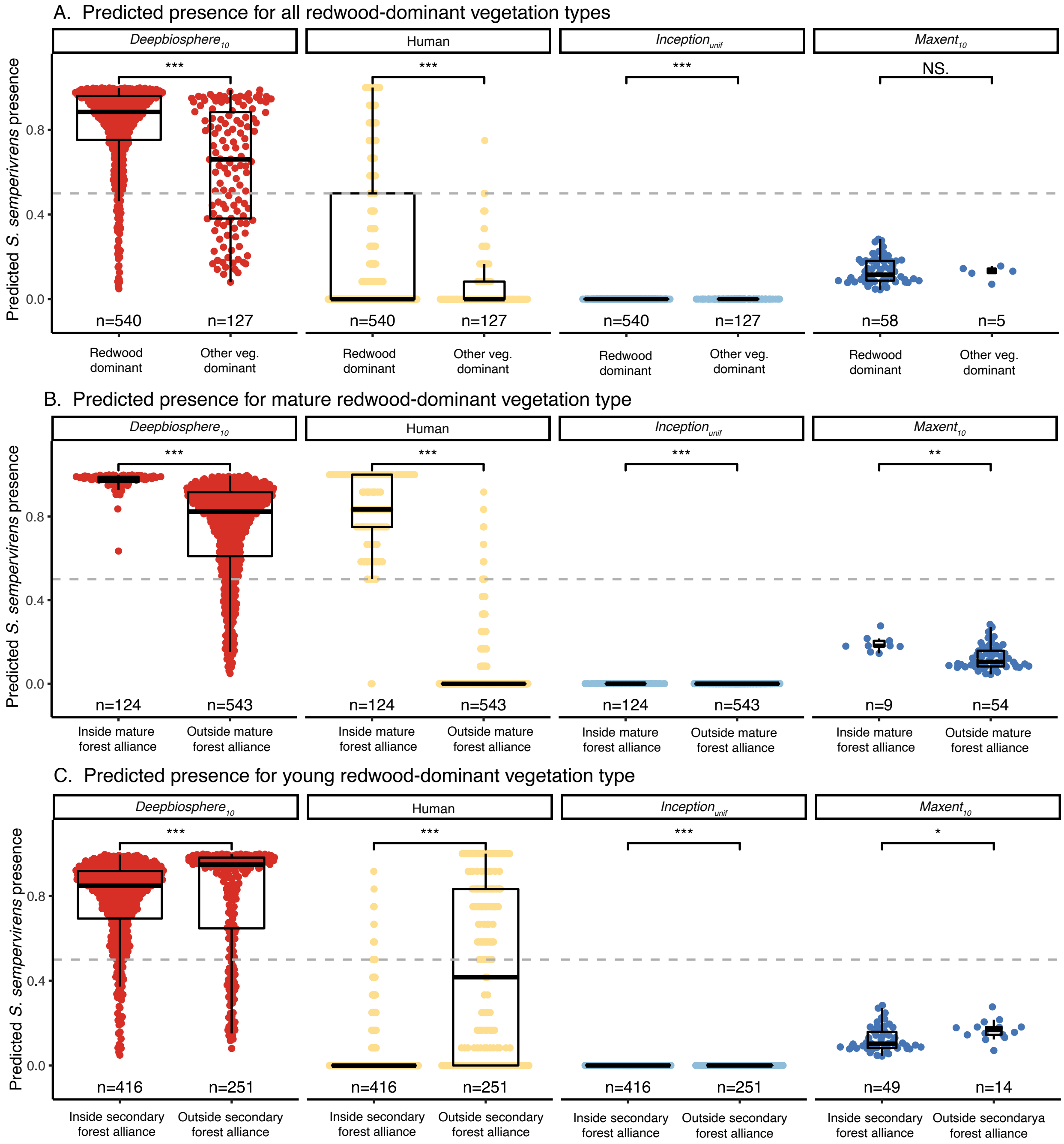

Fig. S10 | Humans can correctly detect mature but not young redwood groves

(**A**) Comparison of the predicted presence of *S. sempervirens* at every pixel for *Deepbiosphere*$_{10}$ and other models based on whether the associated vegetation class for said pixel is redwoods-dominant using the map from **Fig. S11A** at 256 m resolution. *Deepbiosphere*$_{10}$ and *Maxent*$_{10}$ were fitted without any examples from the region, while *Inception*$_{unif}$ did see examples from the study area during training. Using a 0.5 presence-absence threshold, (grey line) for assessing the binary classification accuracy, *Deepbiosphere's* true positive rate is 92.0%, true negative rate is 36.2%, and overall classification accuracy is 81.4%. The human annotator's aggregated true positive rate is 23.5%, true negative rate is 99.2%, and overall classification accuracy is 37.9%. *Inception's* true positive rate is 0.0%, true negative rate is 100.0%, and overall classification accuracy is 19.0%. *Maxent's* true positive rate is 0.0%, true negative rate is 100.0%, and overall classification accuracy is 7.9%. *Deepbiosphere* has the highest classification accuracy and in general, *Deepbiosphere*$_{10}$ is the only model to predict pixels coded as redwood-dominant as likely to contain redwoods, on average. While *Deepbiosphere*$_{10}$ also predicts many pixels not coded as redwood-dominant as likely containing redwoods, redwoods may indeed be present in these areas, just not as the dominant species. (**B**) Comparison of the

predicted presence of *S. sempervirens* at every pixel for $Deepbiosphere_{10}$ and other models based on whether the associated vegetation class for pixels coded as *S. semperivens* Mature Forest alliance using the official National Park Service vegetation map for the region (**Fig. S11A)** at 256 m resolution. Using a 0.5 presence-absence threshold, (grey line) for assessing the binary classification accuracy, *Deepbiosphere's* true positive rate is 100.0%, true negative rate is 16.4%, and overall classification accuracy is 31.9%. The human annotator's aggregated true positive rate is 93.5%, true negative rate is 97.8%, and overall classification accuracy is 97.0%. *Inception's* true positive rate is 0.0%, true negative rate is 100.0%, and overall classification accuracy is 81.4%. *Maxent's* true positive rate is 0.0%, true negative rate is 100.0%, and overall classification accuracy is 85.7%. When only looking at mature redwood groves, human annotators trained using the examples in **Fig. S9** do a better job at distinguishing mature redwood-coded pixels than any other model. $Deepbiosphere_{10}$ has a lower overall accuracy because it predicts many non-mature pixels as containing redwoods, likely detecting pixels that contain secondary growth redwood groves, while both the $Inception_{unif}$ and $Maxent_{10}$ baselines fail to detect any redwoods in the area. (**C**) Comparison of the predicted presence of *S. sempervirens* at every pixel for $Deepbiosphere_{10}$ and other models based on whether the associated vegetation class for pixels coded as *S.sempervirens*-(Other) YG Mixed Forest alliance using the official National Park Service vegetation map for the region (**Fig. S11A)** at 256 m resolution. Using a 0.5 presence-absence threshold, (grey line) for assessing the binary classification accuracy, *Deepbiosphere's* true positive rate is 89.7%, true negative rate is 18.3%, and overall classification accuracy is 62.8%. The human annotator's aggregated true positive rate is 2.6%, true negative rate is 53.4%, and overall classification accuracy is 21.7%. *Inception's* true positive rate is 0.0%, true negative rate is 100.0%, and overall classification accuracy is 37.6%. *Maxent's* true positive rate is 0.0%, true negative rate is 100.0%, and overall classification accuracy is 22.2%. *Deepbiosphere* is the only approach that is able to detect young secondary regrowth redwood forest. Stars indicate an unpaired student's *t*-test with *** indicating a *P*-value of $< 10^{-3}$, ** indicating a *P*-value of $< 10^{-2}$, * indicating a *P*-value of $< 10^{-1}$, and NS. indicating a non-significant *P*-value. Species maps are generated at 256 m resolution or lower to minimize spatial autocorrelation. Batch size, learning rate, and epoch of evaluation can be found in **Table S12**.

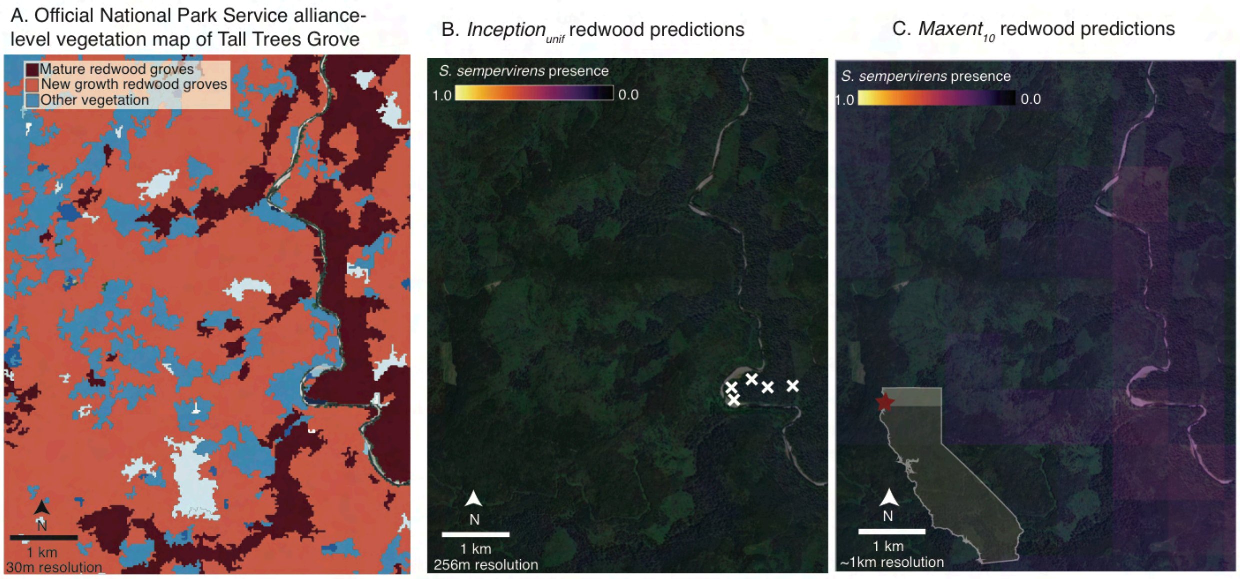

Fig. S11 | Baseline SDMs cannot detect redwoods in Tall Trees Grove

(A) Official National Park Service alliance-level vegetation map of study area (51). Most of the cover in the area is redwoods-dominated with a few other vegetation classes interspersed. **(B)** *Inception$_{unif}$* predictions of redwoods in study area. Since the *Inception* model is trained to predict one species exclusively at a time (courtesy of the softmax transformation in the cross-entropy loss function), the model's outputs cannot be interpreted as species distribution maps reliably as the predicted probability per-class is dependent on the value of other species's predicted presence. Even in this example where *Inception$_{unif}$* has been trained using redwood observations from inside the study area (white xs), *Inception$_{unif}$* still doesn't predict any pixel as present, even for pixels it has seen as containing redwoods before thanks to the softmax transformation. Model weights from the 5th epoch of training were used to generate the area-wide predictions. **(C)** *Maxent$_{10}$* predictions of redwoods in the study area. *Maxent* also does not predict any pixel as present in the study area, likely because it was not trained with any observations in the 10th spatial cross-validation band (light grey region in inset). *Maxent* can reliably extrapolate to new regions if those regions have a similar climate profile to the regions used for fitting. However, the southern and northern populations of redwoods occupy quite dissimilar climates, with the northern population that is largely contained in the 10th cross-validation band living in a much cooler and at times wetter environment than the populations in the Central Coast and Santa Cruz mountains. However, while the climates between populations may differ strongly, the shape of the redwood canopy from remote sensing imagery is still quite similar across environments (see **Fig. S9** for examples from diverse groves), lending more extrapolative prediction power to remote-sensing based modeling approaches. Batch size, learning rate, and epoch of evaluation can be found in **Table S12**.

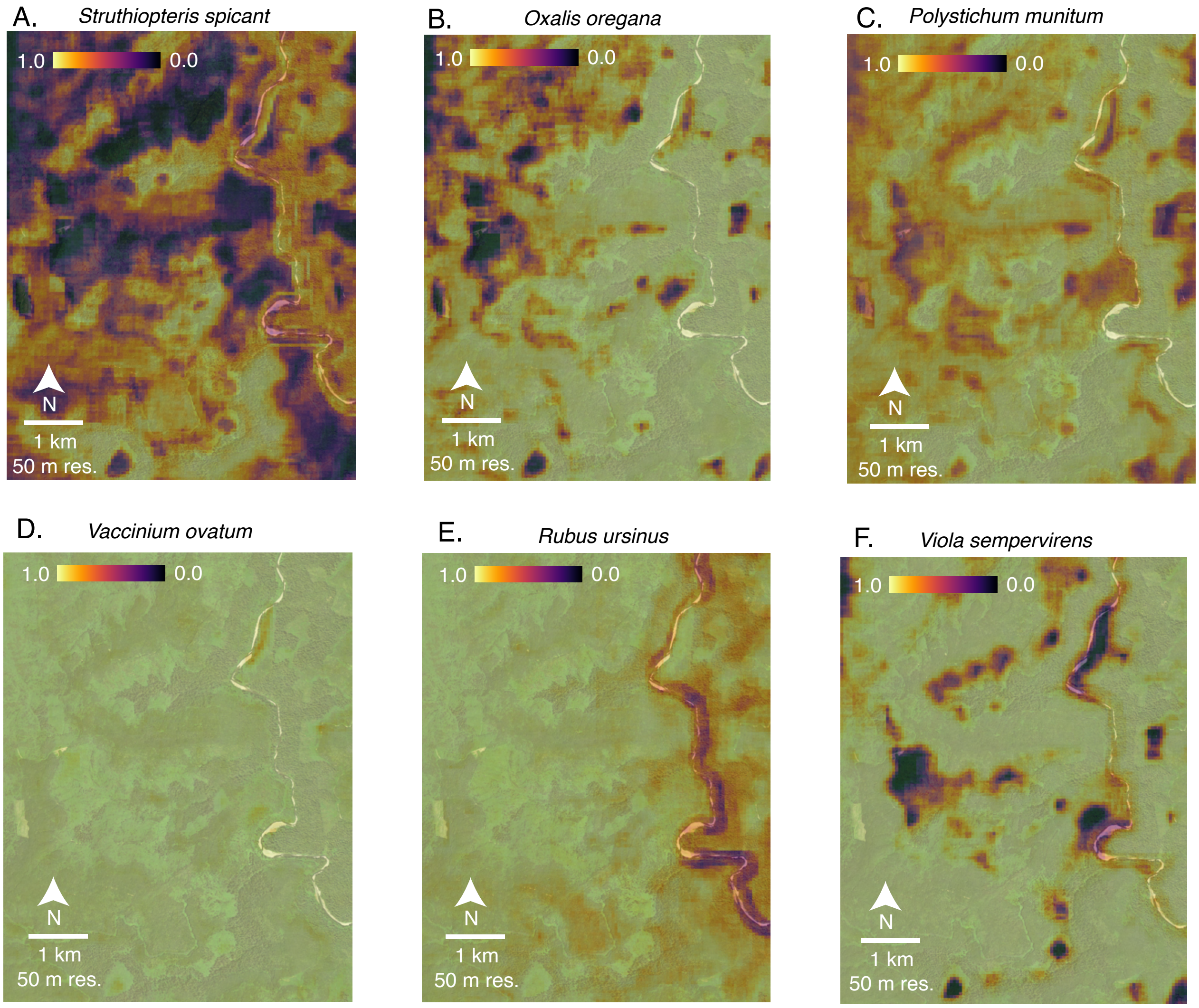

Fig. S12 | *Deepbiosphere*-generated species presence maps for six redwood understory and associated species
(A,B) Species presence maps for *Struthiopteris spicant* and *Oxalis oregana*, respectively. In refs. (51) and (52), both species were shown to have a higher on average constancy (relative number of times the species was observed in field plots of that vegetation type) in mature redwood field plots compared to secondary growth redwood field plots. **(C,D)** Species presence maps for *Polystichum munitum* and *Vaccinium ovatum*, respectively. In refs. (51) and (52), both species had similar average constancies in both mature redwood field plots compared to secondary growth redwood field plots. **(E,F)** Species presence maps for *Rubus ursinus* and *Viola sempervirens*, respectively. In refs. (51) and (52), both species were shown to have a higher on average constancy in secondary-growth redwood field plots compared to mature redwood field plots. Batch size, learning rate, and epoch of evaluation can be found in **Table S12**.

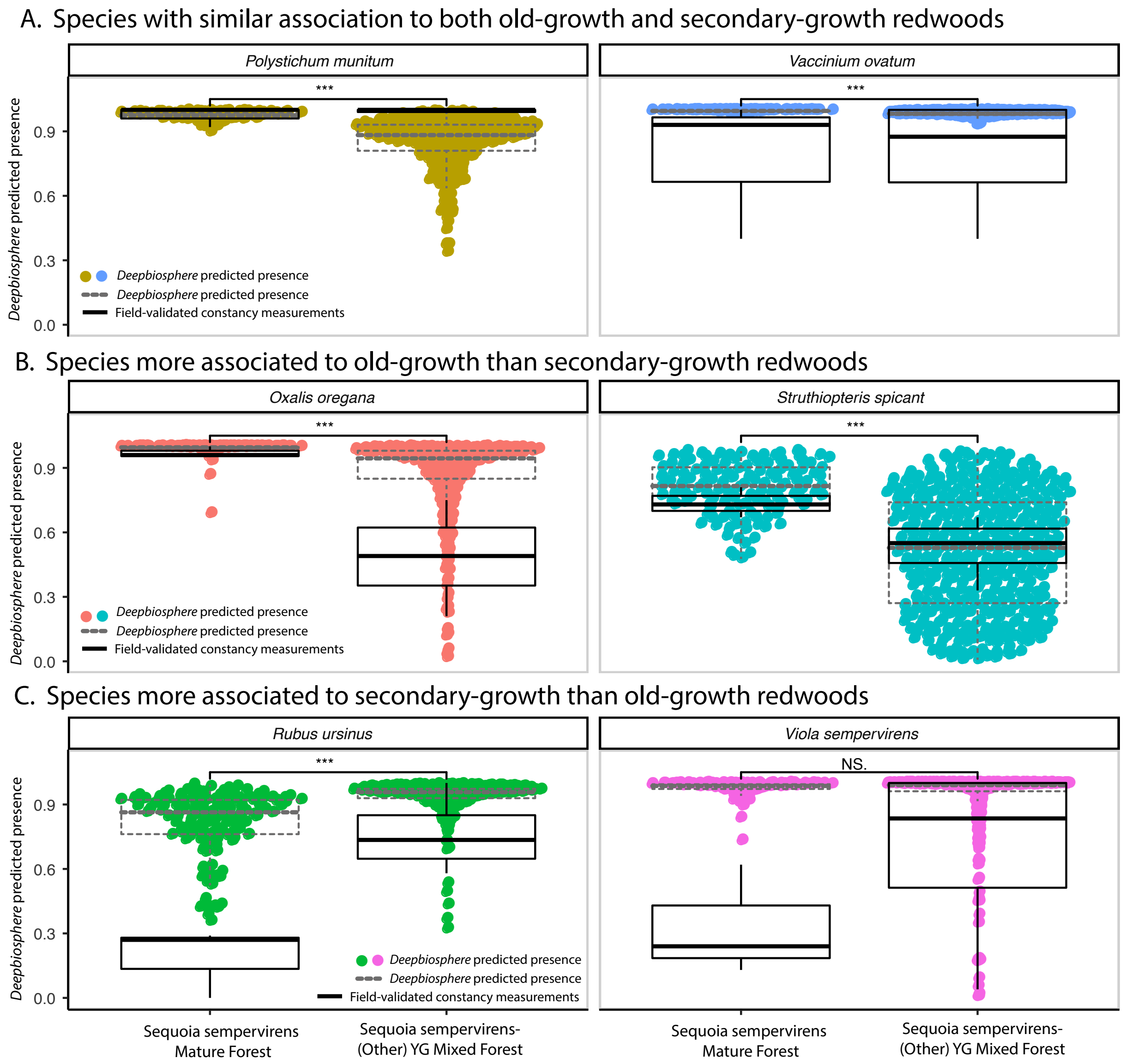

Fig. S13 | *Deepbiosphere* prediction breakdown by forest age for six understory species

Comparing how *Deepbiosphere's* predictions of understory species line up to field-validated measurements of species' relative frequencies (constancy) in both mature and secondary-growth redwood forests. (**A**) Two understory species that co-occur with both old-growth and secondary-growth redwoods. (**B**) Two understory species that co-occur more frequently with old-growth redwoods than secondary-growth redwoods. (**C**) Two understory species that co-occur more frequently with secondary-growth redwoods than old-growth redwoods. For some species (*S. spicant, V. ovatum, P. munitum*), *Deepbiosphere's* predicted presence values are well-calibrated to field-validated presence. For others, (*R. ursinus, O. oregana*), *Deepbiosphere's* predicted presence are higher on average than the field-validated constancies, but the expected relationship between old-growth and secondary-growth-mapped pixels still holds (e.g. *O. oregana* still has a significantly higher predicted presence in old-growth versus secondary-growth redwoods, matching the constancy trends). Finally, *V. sempervirens* is predicted quite broadly and despite having a higher predicted presence in secondary-growth forest (0.990) compared to old-growth forest (0.986) similar to the constancy relationship, the difference between old-growth and secondary-growth predictions isn't significant. Co-occurrence relationships were determined from constancy tables in refs. (51, 52) (**SM 4.4**) and within these studies, constancy values were determined with between 5-24 plots, depending on the association. Predictions were generated at 256 m resolution from *Deepbiosphere* to minimize spatial autocorrelation, then classified by largest overlapping alliance-level vegetation type from ref. (51) and filtered to only keep pixels from redwood-dominant alliances (*Sequoia sempervirens*-(Other) YG Mixed Forest or *Sequoia sempervirens* Mature Forest). Box and whisker plots for *Deepbiosphere's* predictions are overlaid in hatched grey. Box and whisker plots of constancy values from refs. (51, 52) are overlaid in black (**SM 4.4**). YG = young-growth; stars indicate an unpaired student's *t*-test with *** indicating a *P*-value of $< 10^{-3}$ and NS. indicating a *P*-value of $> 10^{-1}$. Batch size, learning rate, and epoch of evaluation can be found in **Table S12**.

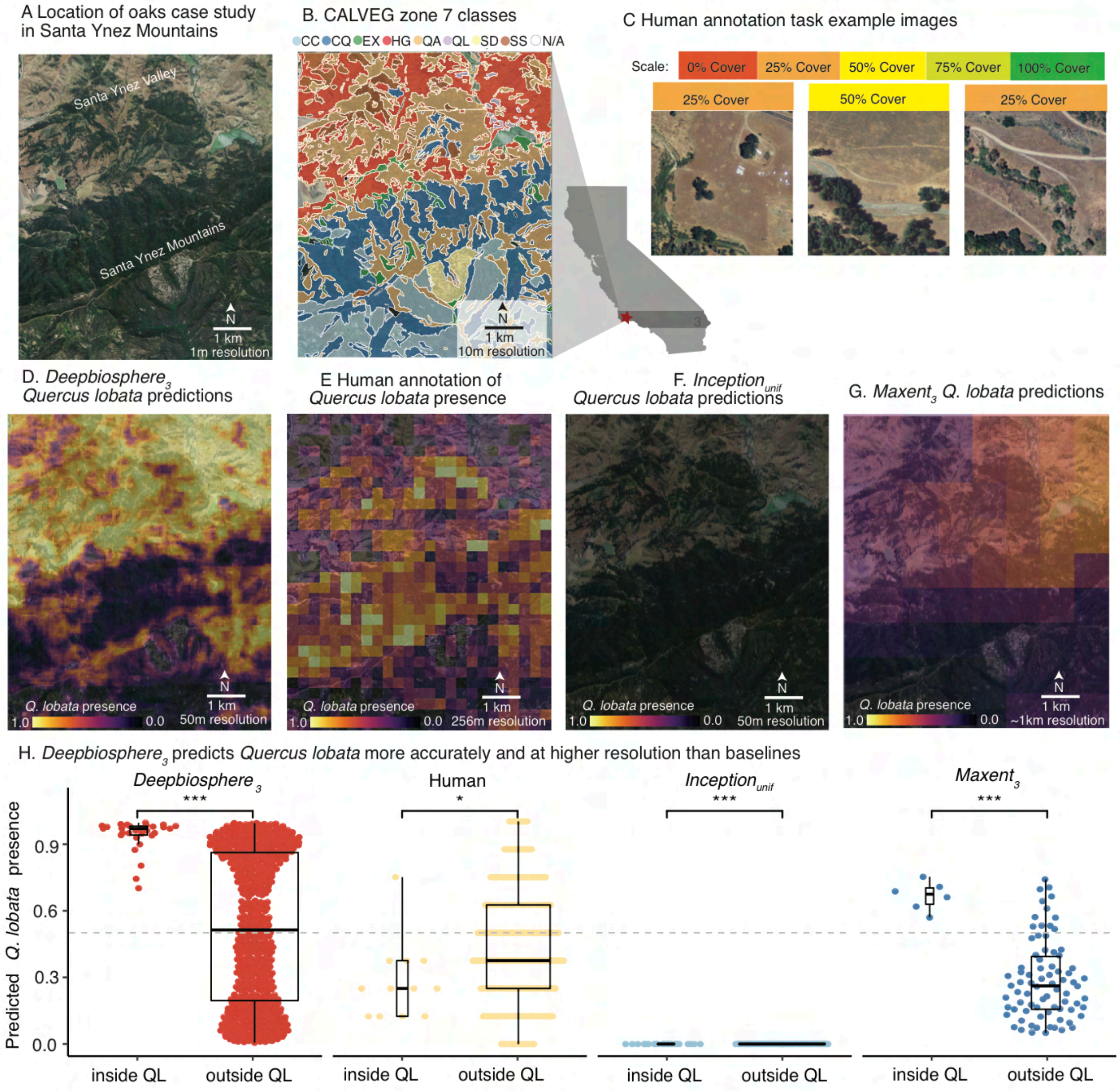

Fig. S14 | Predictions of dominant species in the Santa Ynez Valley and Mountains of Southwest California
(A) The Santa Ynez mountains of Southern California have a rich and varied ecological landscape with many rapid ecosystem transitions—such as dry interior valley savanna to scrubby mountain peak—making it a good case study to explore how well *Deepbiosphere* can capture spatial variation in chaparral ecosystems. **(B)** South Coast existing vegetation map (CALVEG) zone 7 alliance-level vegetation classes for study area (53). Only zones mapping to native vegetation with a sufficient cover in the study area are shown. **(C)** *Quercus lobata* annotation task examples. Detailed information about sites can be found in **Table S11**. **(D)** *Deepbiopshere*$_3$ prediction of *Q. lobata* across the study area. Generally speaking, *Deepbiosphere*$_3$ predicts *Q. lobata* as present in the Santa Ynez valley —its native habitat— and absent in the Santa Ynez mountains. **(E)** Human cover annotations of *Q. lobata* across the study area. Human labelers struggled with correctly labeling squares where *Q. lobata* is expected to be found, favoring the chaparral scrub of the Santa Ynez foothills over the valley floor where the oaks are actually found. **(F)** The *Inception*$_{unif}$ baseline does not predict *Q. lobata* present anywhere within the study area, a consequence of its softmax-based loss function. **(G)** The *Maxent*$_3$ baseline does predict *Q. lobata* as present in most of the valley pixels, but has a much lower resolution than *Deepbiosphere*$_3$, making it impossible for the model to disambiguate the wooded hills centered in the valley from the grassland portions of the valley. **(H)** Comparison of *Q. lobata* presence annotations per-pixel shows that *Deepbiosphere*$_3$ and *Maxent*$_3$ are the only SDMs that correctly disambiguate pixels annotated as *Q. lobata* habitat (QL) by the CALVEG vegetation map from those not annotated as such. Pixels are labeled as *Q. lobata* habitat if any part of the pixel intersects any CALVEG polygon of class QL (*Q. lobata*). Stars indicate an unpaired student's *t*-test per-pixel with *** indicating a *P*-value of $< 10^{-3}$, ** indicating a *P*-value of $< 10^{-2}$, * indicating a *P*-value of $< 10^{-1}$, and NS. indicating a non-significant *P*-value. Species maps were generated at 256 m resolution or lower to minimize spatial autocorrelation and batch size, learning rate, and epoch of evaluation can be found in **Table S13**.

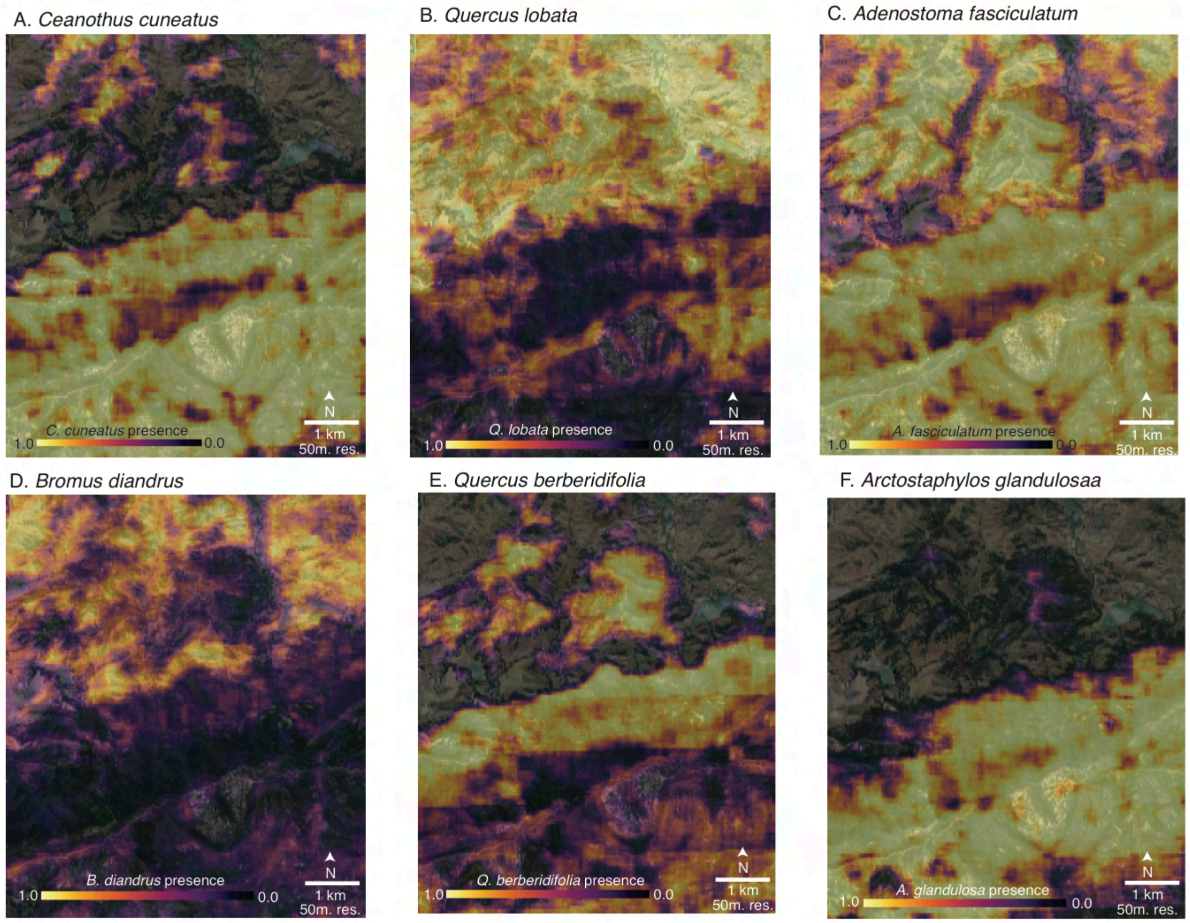

Fig. S15 | *Deepbiosphere*-generated species presence maps for six chaparral indicator species
(A) Species presence map for *Ceanothus cuneatus* (buckbrush), an indicator or associated species for CALVEG zones *Ceanothus* chaparral alliance (CC), lower montane mixed chaparral alliance (CQ), and coastal mixed hardwood alliance (EX). **(B)** Species presence map for *Quercus lobata* (valley oak), an indicator species for the valley oak alliance (QL). **(C)** Species presence map for *Adenostoma fasciculatum* (chamise), an indicator or associated species for the coast live oak alliance (QA), *Ceanothus* chaparral alliance (CC), coastal mixed hardwood alliance (EX), lower montane mixed chaparral alliance (CQ), and California sagebrush alliance (SS). **(D)** Species presence map for *Bromus diandrus* (great brome), an indicator species for the annual grasses and forbs alliance (HG). **(E)** Species presence map for *Quercus berberidifolia* (scrub oak) an associated species in the lower montane mixed chaparral (CQ) alliance. **(F)** Species presence map for *Arctostaphylos glandulosa* (eastwood manzanita), an associated species in the lower montane mixed chaparral (CQ) and manzanita chaparral (SD) alliances. Species maps were generated at 256 m resolution or lower to minimize spatial autocorrelation and batch size, learning rate, and epoch of evaluation can be found in **Table S13**.

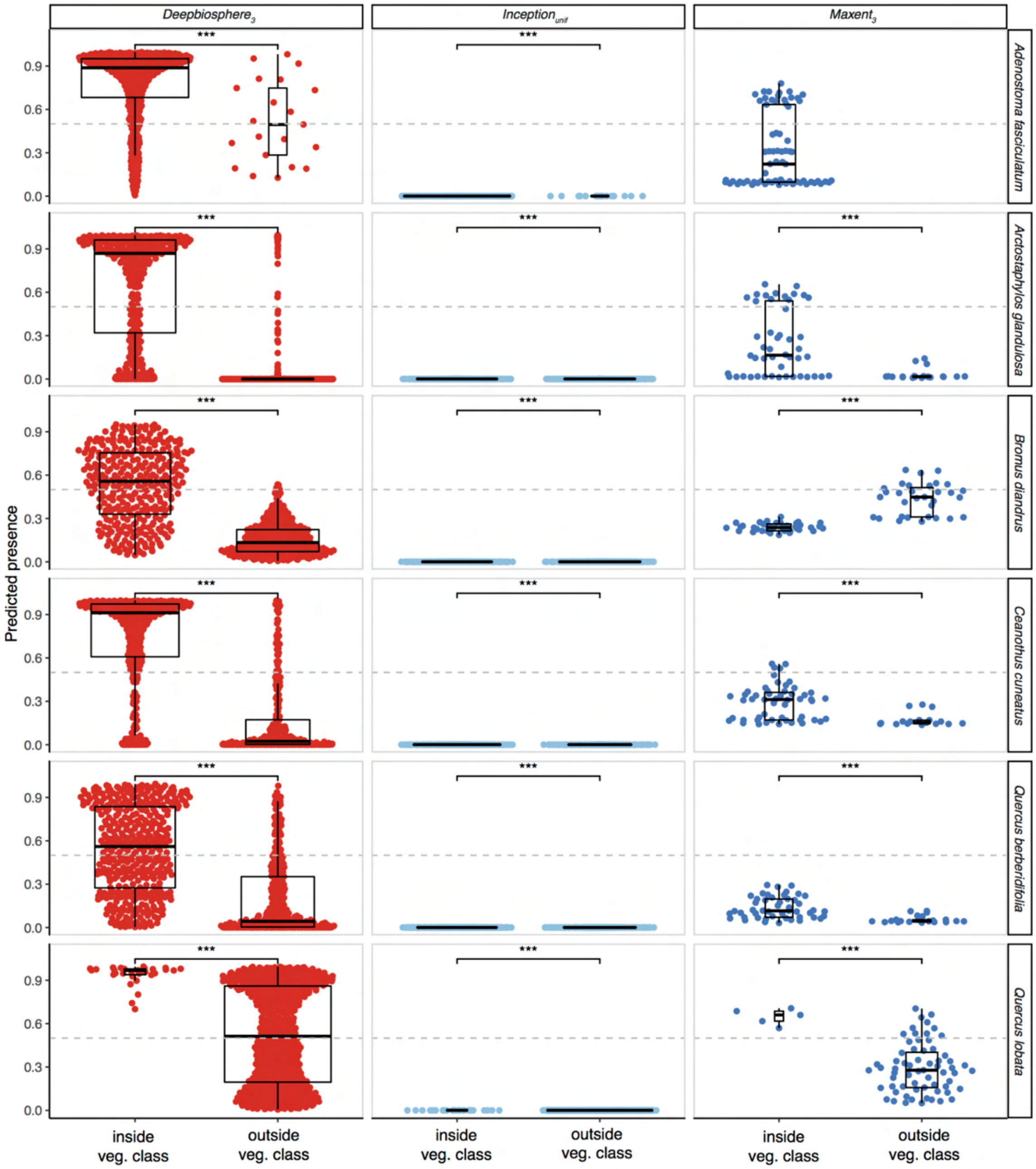

Fig. S16 | *Deepbiosphere* prediction comparisons to baseline SDMs for six chaparral indicator species

Per-pixel comparison of indicator species presence using the USDA Forest Service's South Coast existing vegetation map (CALVEG) (53) for *Deepbiosphere*$_3$ and two baseline SDMs. On average, *Deepbiosphere*$_3$ does a good job of correctly predicting species as present in the pixels located within their associated CALVEG habitats (**Fig. S15B**). All species are predicted as present (above 0.5) on average for pixels inside the associated habitats and as absent (below 0.5) for pixels outside of the associated CALVEG habitats. Meanwhile, both *Maxent*$_3$ and *Inception*$_{unif}$ fail this test for all but one case (*Maxent*$_3$ modeling *Q. lobata*). Pixels are labeled as inside habitat if any part of the pixel intersects any CALVEG polygon from the USDA Forest Service's South Coast existing vegetation map (53) where said species is mentioned in the CALVEG Zone 5 vegetation description for that class (54). Stars indicate an unpaired student's *t*-test per-pixel where *** means a *P*-value of $<10^{-3}$. Species maps were generated at 256 m resolution or lower to minimize spatial autocorrelation and batch size, learning rate, and epoch of evaluation can be found in **Table S13**.

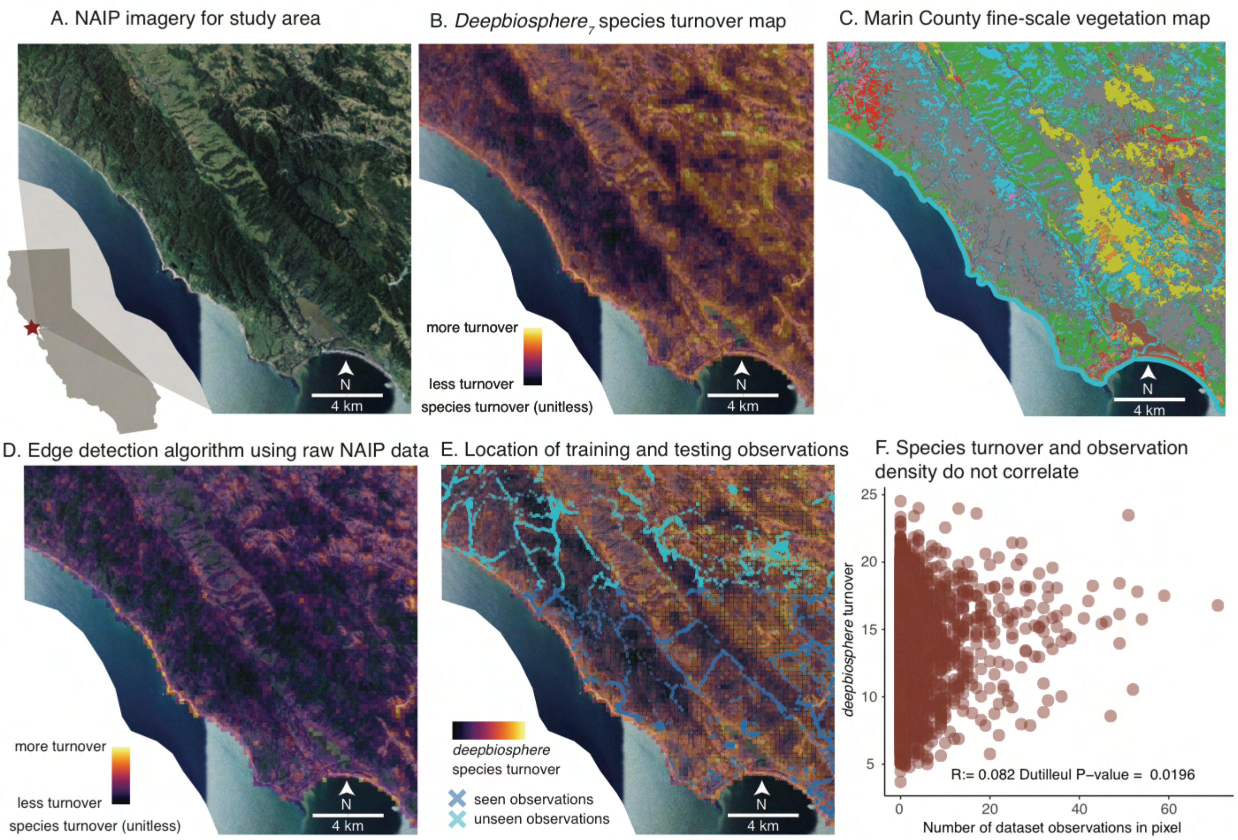

Fig. S17 | *Deepbiosphere* predicts spatial community change in northern Marin county
(A) Northern Marin County is very ecodiverse, located at the boundary between the Coast Range and Central California Foothills and Coastal Mountains level 1 EPA ecoregions, thus making it an ideal location to study spatial transitions in plant communities. NAIP aerial imagery of the area confirms visually that the landscape is highly varied with beach, forest, and grassland ecosystems visible. **(B)** Using the edge detection algorithm from **Fig. S19**, predictions of spatial community change can be generated using *Deepbiosphere*$_7$. These predictions appear to capture rapid spatial plant community composition changes, including beach to forest and forest to grassland. **(C)** An independently-generated fine-scale map of vegetation and land use in the area confirms that the region contains many unique ecosystems and transitions (57). The color key can be found in **Fig. S18**. **(D)** Running the same edge detection algorithm from **Fig. S19** using the raw NAIP bands as input (A) generates a map of the average difference in color between nearby NAIP pixels. Visually, the map looks quite different from the *Deepbiosphere*-generated spatial change predictions, implying that *Deepbiosphere*$_7$ can detect far more than just simple pixel color changes. **(E)** Mapping the location of observations both seen and unseen during training shows that the spatial community change visually also does not appear to naively recapitulate locations it has seen before. **(F)** Correlating the number of observations per-pixel with the spatial community change as predicted by *Deepbiosphere*$_7$ confirms that the spatial community change metric is capturing real changes in the community rather than spurious correlations to either raw pixel greenness (E) or previously seen observations (F). Predictions were generated from *Deepbiosphere* trained with a learning rate of $1x10^{-4}$ and a batch size of 150 evaluated at epoch 5.

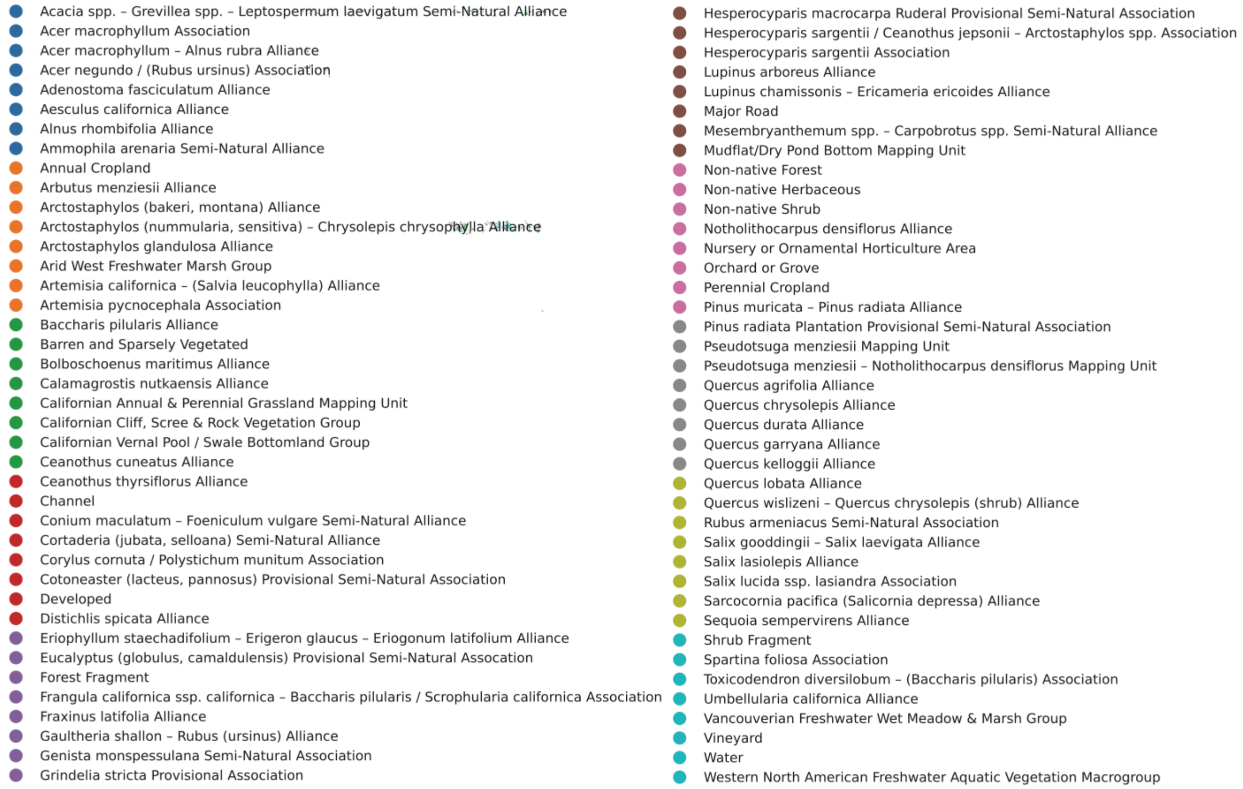

Fig. S18 | Legend for fine-scale Marin vegetation map

Color key for fine scale vegetation map of northern Marin county (57). The map contains 80 unique classes, so a circular color scale was utilized to aid visualization.

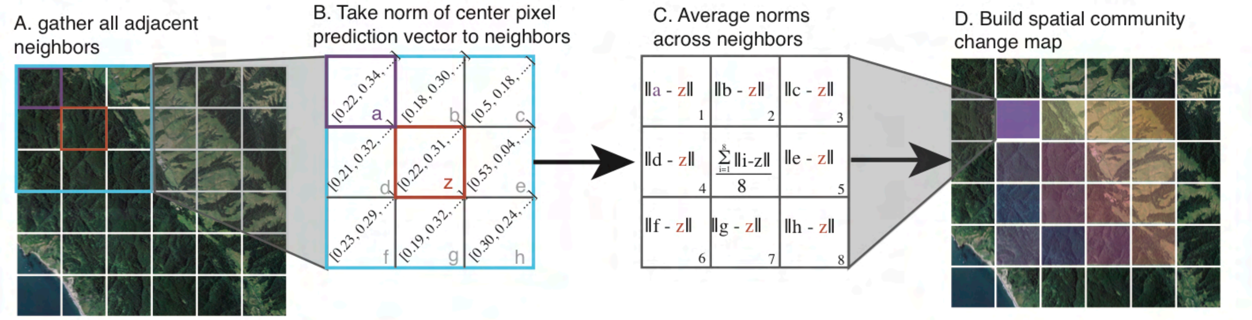

Fig. S19 | Spatial community change algorithm visual explanation

Visual explanation of spatial community change detection algorithm inspired by edge detection filters from computer vision. **(A)** First, the algorithm identifies images adjacent to a given 256 x 256 m square **(B)** Next, taking the predicted probabilities from *Deepbiosphere,* the distance between the predictions made for each neighboring cell and the central pixel is calculated. **(C)** Then, the norm of these differences is used to generate the average local neighborhood change for the central pixel. (**D**) Convolving this norm-of-neighbors pixel-by-pixel generates the full map of spatial community change at the same resolution as the original map, with a one pixel buffer.

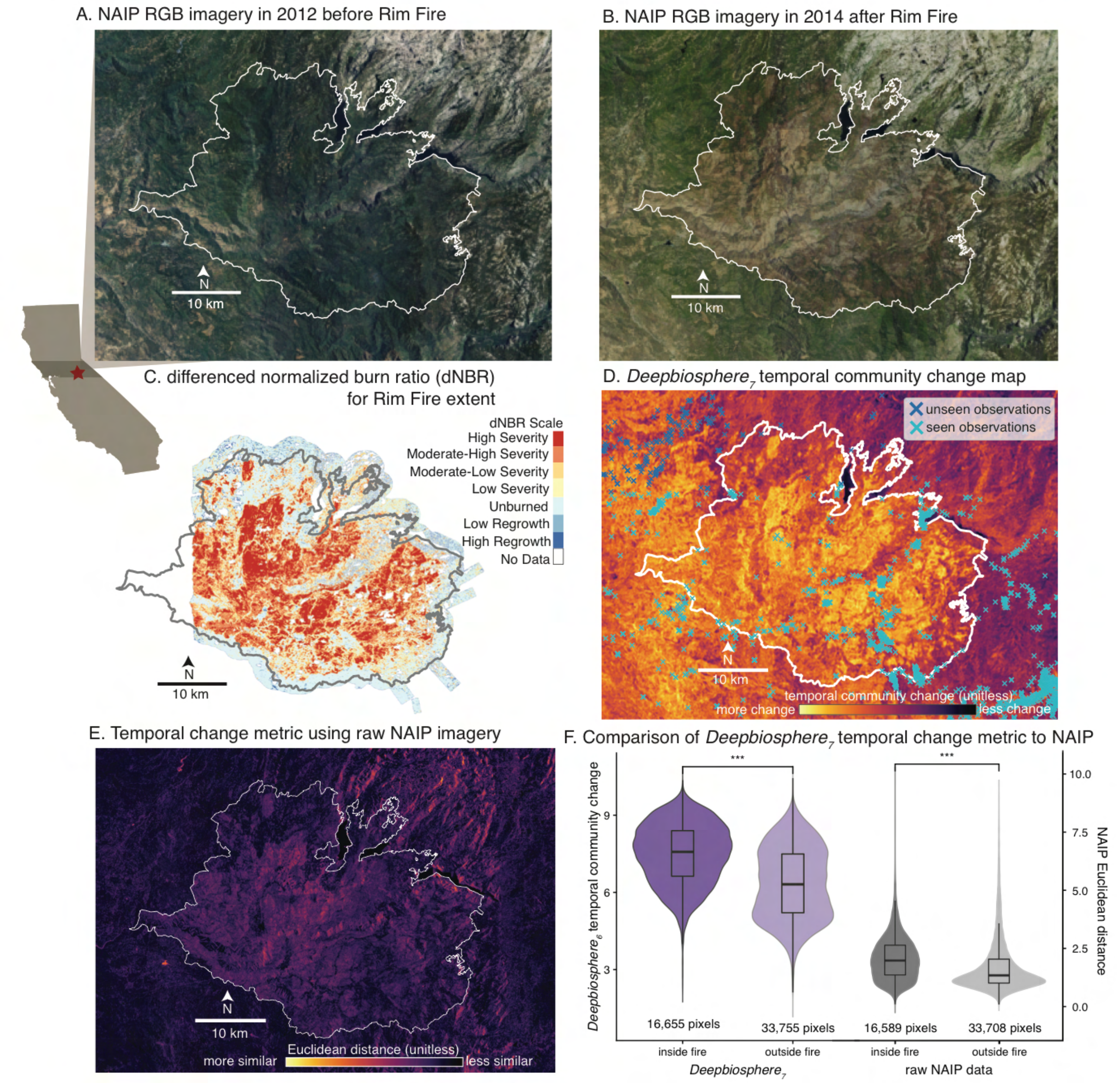

Fig. S20 | Detecting rapid temporal plant community change after a major California wildfire
(A) NAIP imagery from the Sierra Mountain foothills in eastern California in 2012. This area was the site of the major Rim Wildfire in 2013, which at the time was the second-largest wildfire in California history. **(B)** NAIP imagery of 2014 after the 2013 Rim Fire, with boundary outlined in white. Comparing (A) and (B), the burn scar of the wildfire is very visible within the fire perimeter compared to regions outside the burn scar. **(C)** The differenced normalized burn ratio (dNBR) for part of the fire area from ref. (60). The dNBR metric estimates fire severity using a normalized scale based on differences in green and near infrared band wavelengths from hyperspectral data collected before vs. after the fire. Only part of the region was imaged before the fire, so the dNBR coverage does not cover the entire burn scar. **(D)** Temporal community change as predicted by *Deepbiosphere*$_7$ with observations used to train *Deepbiosphere*$_7$ overlaid. **(E)** Temporal Euclidean distance calculated using raw NAIP Red-Green-Blue-Infrared imagery. Visually, the raw pixel difference across years does not appear to match the burn severity metric near as well as the *Deepbiosphere*-based temporal community change prediction. This is curious given that the infrared and green NAIP bands used to calculate the Euclidean distance are nearly the same wavelength as the bands used to calculate dNBR. **(F)** Comparing both the *Deepbiosphere*-based and NAIP-based temporal Euclidean distance predictions per-pixel from inside vs. outside the fire. On average, the change predictions were higher within versus outside the fire bounds as expected (unpaired student's *t*-test; *P*-values $< 2.2 \times 10^{-16}$). Predictions were generated from *Deepbiosphere* trained with a learning rate of 1×10^{-4} and a batch size of 150 evaluated at epoch 5.

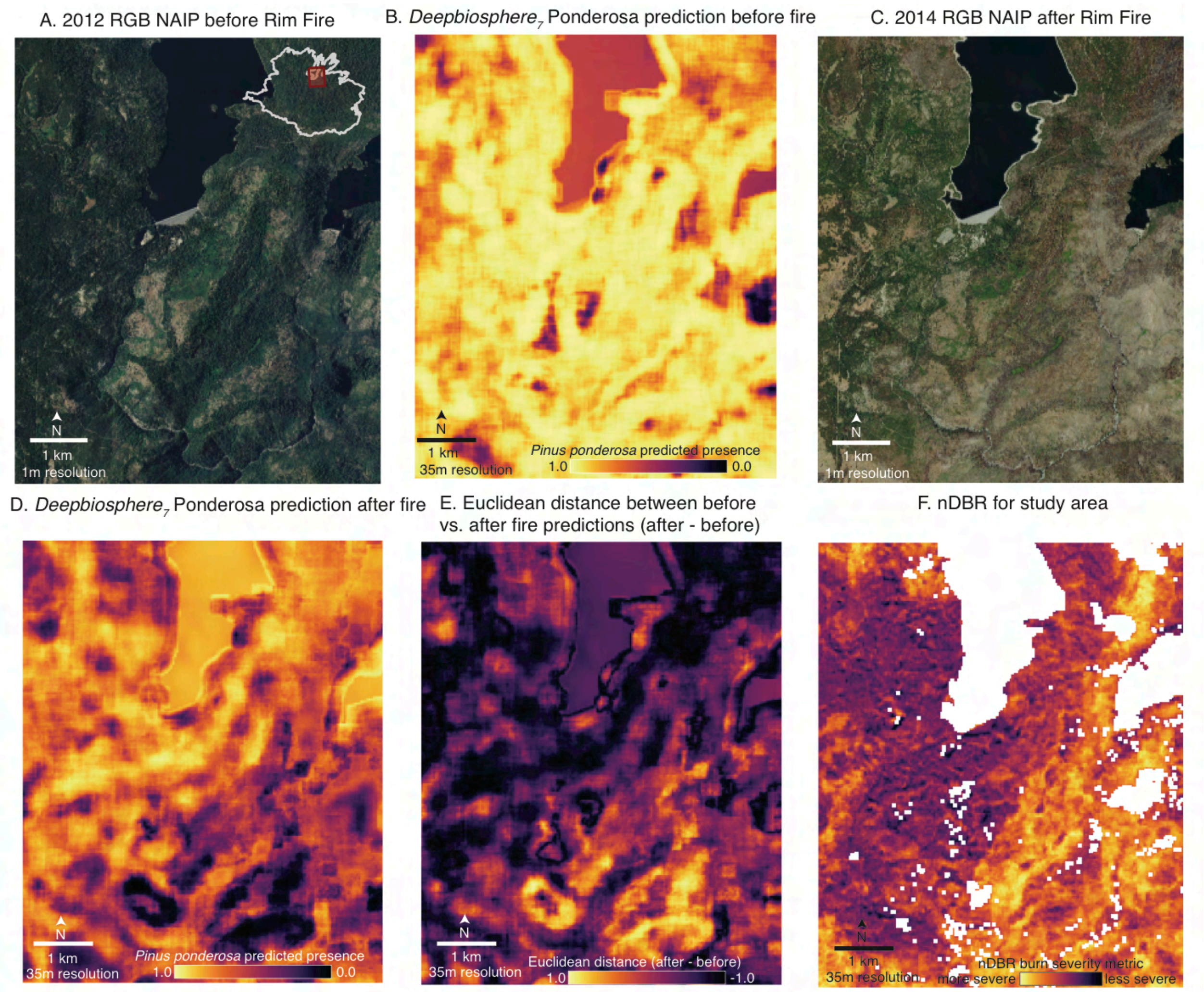

Fig. S21 | Example of temporal Euclidean distance calculation

(A) NAIP imagery before fire in a subset of the fire's extent (location inside perimeter of fire inset). **(B)** *Deepbiosphere*$_7$ predicted probabilities for *Pinus ponderosa* (ponderosa pine) before the fire, using the imagery in (A). **(C)** NAIP imagery after the fire from the same geographic location. Visually, locations in the bottom right of the image appear to have suffered the most severe burning, while forests on the left-hand side of the area have been somewhat spared. **(D)** *Deepbiosphere*$_7$ predicted probabilities for *P. ponderosa* after the fire. Predicted probabilities on the left-hand side of the area have not changed substantially while the more severely burned lower right quadrant now has *P. ponderosa* predicted mostly as absent. **(E)** To capture this temporal change in *P. ponderosa* presence, the Euclidean distance between the probabilities in (B) and (D) are calculated per-pixel. For the one-dimensional case (only one species at a time) the Euclidean distance simplifies to the difference between $\hat{p}_{2012}$ and $\hat{p}_{2014}$. Extending this difference calculation across all species in the dataset and taking the subsequent norm is how the temporal community change metric is generated, capturing both the magnitude and direction of probability shifts across species and across time. (F) Here, the empirical metric of burn severity—difference in normalized burn ratio (dNBR) (60)—is displayed for the region. Predictions were generated from *Deepbiosphere* trained with a learning rate of $1x10^{-4}$ and a batch size of 150 evaluated at epoch 5.

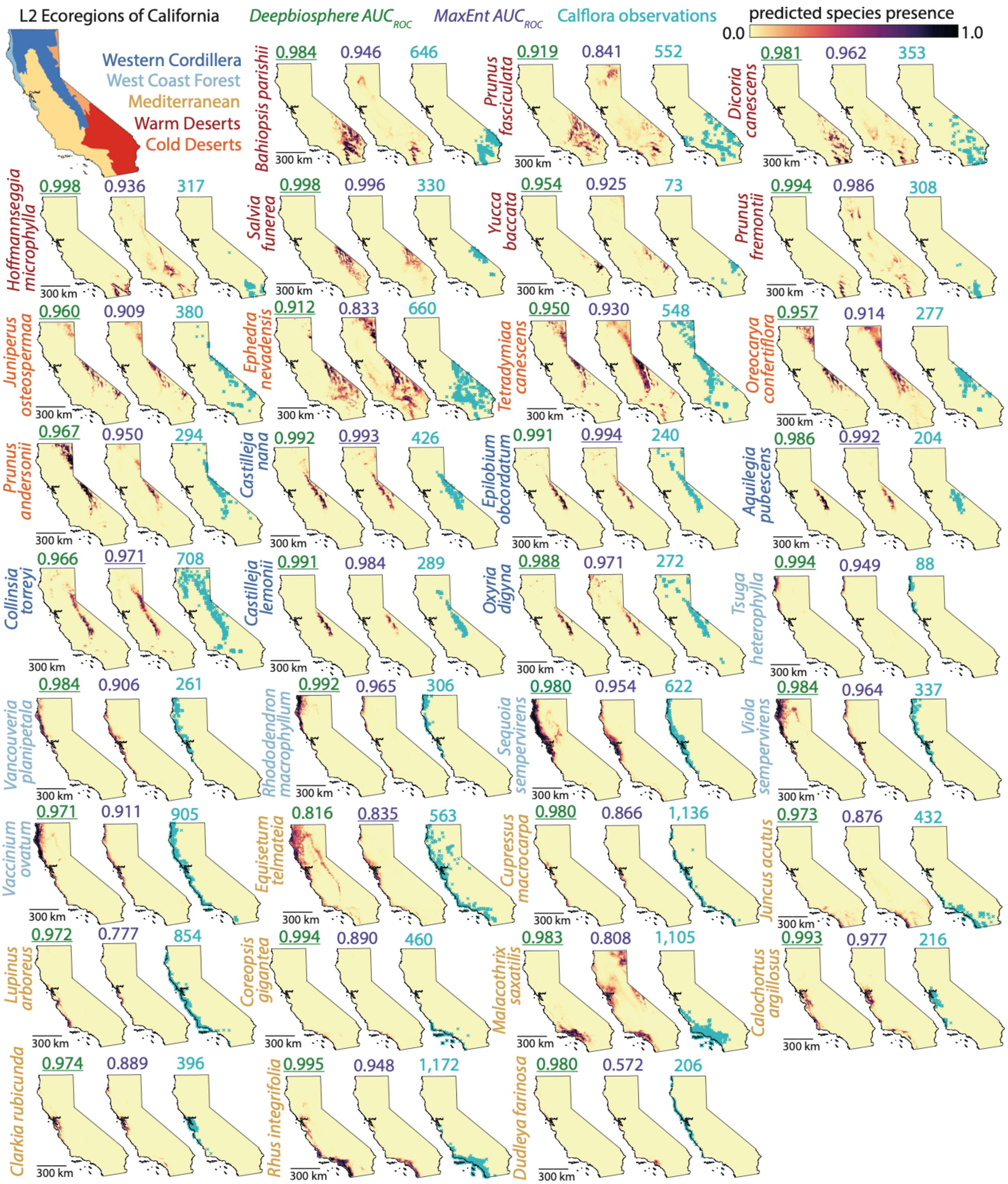

Fig. S22 | Species range maps for a subset of well-predicted species across California.
Range maps of 150 m per-pixel for *Deepbisphere* and ~1 km per-pixel for *Maxent* were generated using the same model evaluated in **Table 1** and **Figure 1**, namely *Deepbiosphere* trained on the uniform split of the dataset with a learning rate of 0.0001 and evaluated at epoch 5. Species were partitioned into the L2 ecoregions that the majority of said species' *Deepbiosphere* training observations were found in, then filtered to only include species for which there were at least 10 observations in the *Deepbiosphere* uniform test set. The top-5 highest accuracy species by AUC_{ROC} on the *Deepbiosphere* uniform test set for both *Deepbiosphere* and *Maxent* were then selected, filtering the top-5 ranking to only include species with an AUC_{ROC} of at least 0.98 for upwards of a total of 10 species per-L2 ecoregion. Not every ecoregion has 10 species plotted if there was overlap between the top-5 best AUC_{ROC}

species for *Deepbiosphere* and *Maxent*, or if one of the two models did not have a high enough AUC_{ROC} on well-supported species. The two model's predictive accuracies were then compared using an independently-derived set of occurrence records from Calflora (64), and in general, we see that *Deepbiosphere* does on average a better job at predicting species' presence at the independently-collected Calflora occurrence locations, with *Deepbiosphere* exhibiting a higher AUC_{ROC} for 29 species as compared to *Maxent's* 5 species (noted using underline). *Deepbiosphere* especially exhibits increased predictive accuracy for relatively range-constrained species with few observations in the study area, such as *Yucca baccata* (73 Calflora observations, 78 *Deepbiosphere* dataset observations, *Tsuga heterophylla* (88 Calflora observations, 78 *Deepbiosphere* dataset observations) and *Clarkia rubicunda*, 396 Calflora observations, 435 *Deepbiosphere* dataset observations).

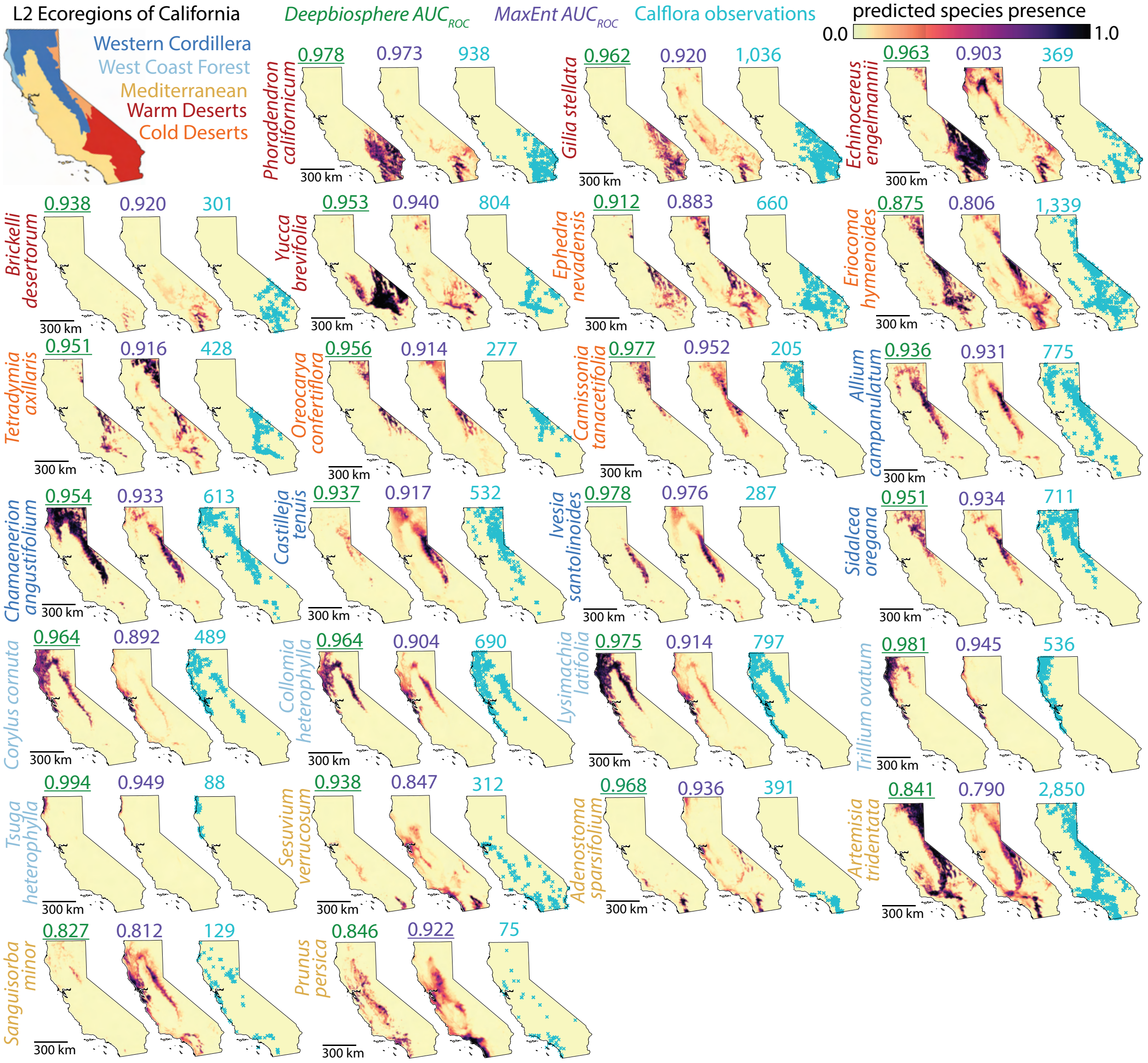

Fig. S23 | Species range maps for a subset of random species across California.
Range maps of 150m per-pixel for *Deepbisphere* and ~1km per-pixel for *Maxent* were generated using the same model evaluated in **Table 1** and **Figure 1**, namely *Deepbiosphere* trained on the uniform split of the dataset with a learning rate of 0.0001 and evaluated at epoch 5. Species were partitioned into the L2 ecoregions that the majority of said species' *Deepbiosphere* training observations were found in, then filtered to only include species for which there were at least 10 observations in the *Deepbiosphere* uniform test set. Five species were chosen at random from each and the two model's predictive accuracies were then compared using an independently-derived set of occurrence records from Calflora (64). Even for randomly-derived species, we see that *Deepbiosphere* does a better job at predicting species' presence at the independently-collected Calflora occurrence locations, with *Deepbiosphere* exhibiting a higher AUC_{ROC} for all but one species compared to *Maxent* (exception is *Prunus persica*, noted using underline).

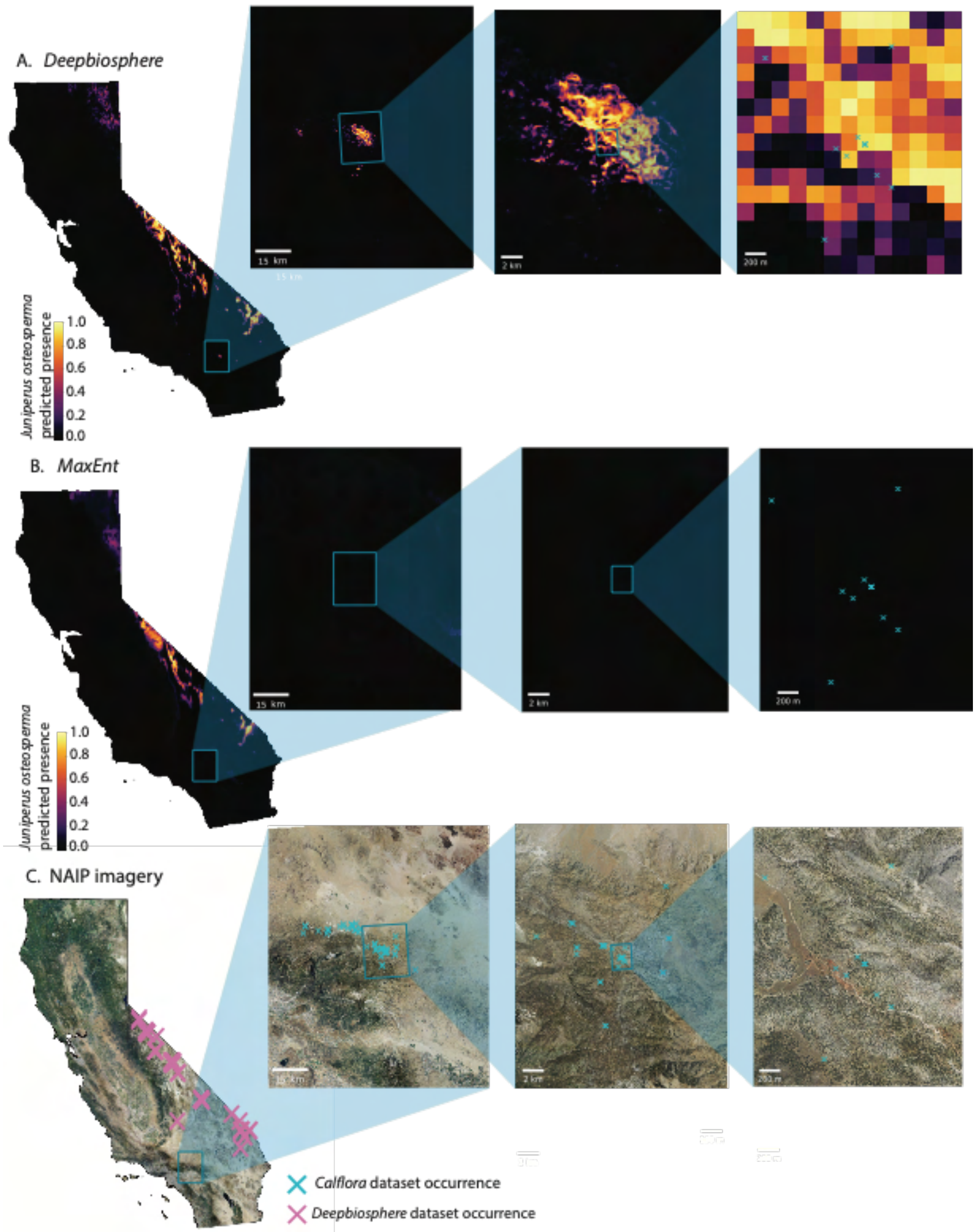

Fig. S24 | High-resolution zoom-in of *Juniperus osteosperma* in Transverse Range.
(A) Progressive ~1 x 1, ~0.1 x 0.1, and ~0.01 x 0.01 degree zoom-ins of the species range maps generated by *Deepbiosphere* at 150m resolution for *J. osteosperma* (see **Fig. S22** for statewide range map). *J. osteosperma* has a very distinctive and visible crown from remote sensing imagery (see highest-resolution inset of (C) for examples), enabling *Deepbiosphere* to detect this population of *J. osteosperma* not included in the training dataset. **(B)** Progressive ~1 x 1, ~0.1 x 0.1, and ~0.01 x 0.01 degree zoom-ins of the species range maps generated by *Maxent* at ~1km resolution for *J. osteosperma* (see **Fig. S22** for statewide range map). *Maxent* does not effectively detect the Transverse Range population of *J. osteosperma.* **(C)** Progressive ~1 x 1, ~0.1 x 0.1, and ~0.0 x 0.01 degree zoom-ins of corresponding NAIP imagery for the same geographic extent overlaid with known species occurrences from both the *Deepbiosphere* training dataset (pink Xs) and a larger collection of contemporary and historic occurrences from *Calflora* (64) (cyan Xs). *Juniperus osteosperma* is present in the Transverse Range (see *Calflora* observations) but has no observations from that region in the *Deepbiosphere* dataset.

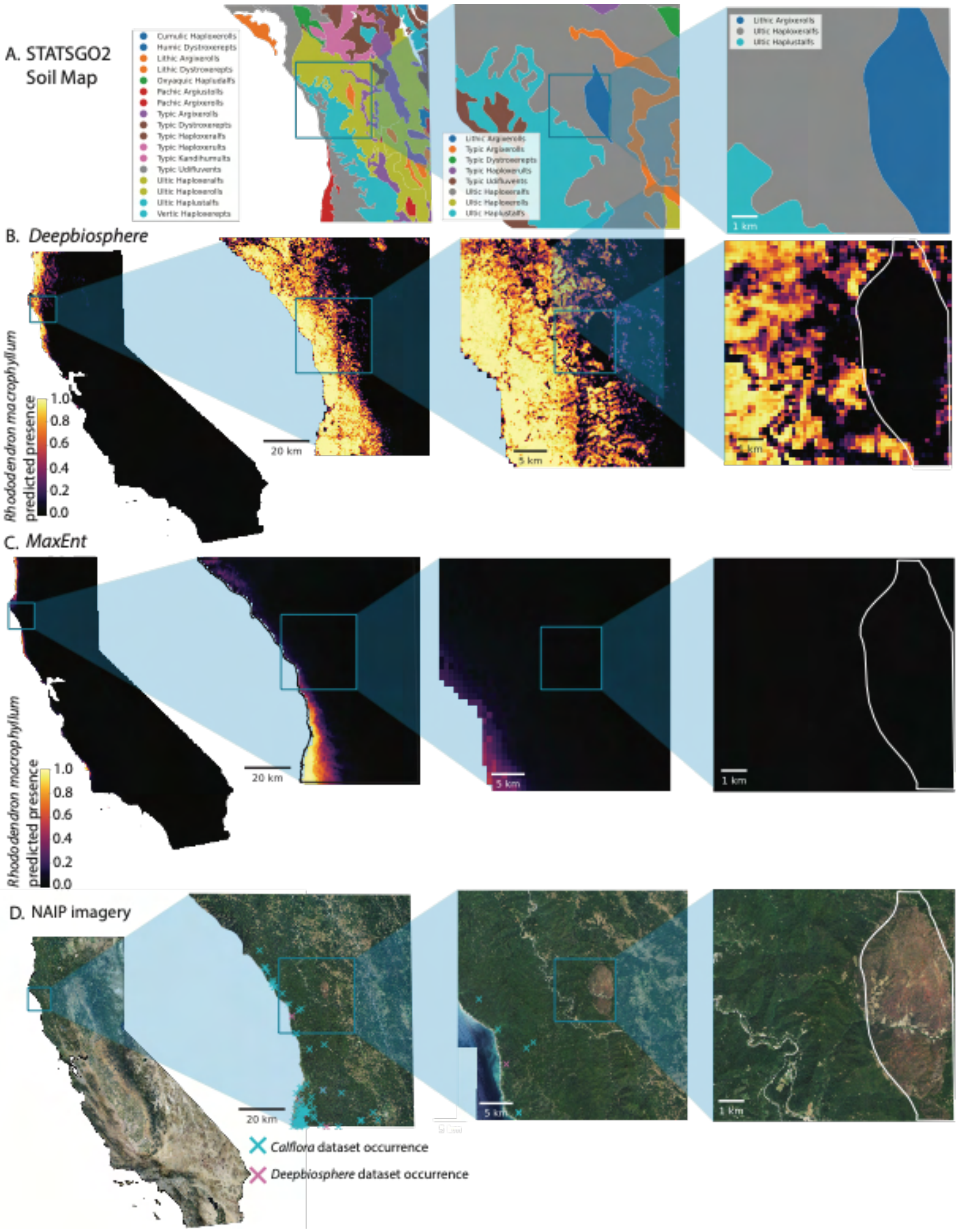

Fig. S25 | High-resolution zoom-in of *Rhododendron macrophyllum* near Red Mountain, Mendocino County.
(A) Progressive ~1 x 1, ~0.1 x 0.1, and ~0.01 x 0.01 degree zoom-ins of soil maps from the USDA's STATSGO2 soil database (65) demonstrating a known area of serpentine soil (Lithic Argixerolls, dark blue). **(B)** Progressive ~1 x 1, ~0.1 x 0.1, and ~0.01 x 0.01 degree zoom-ins of species range maps generated by *Deepbiosphere* at 150m resolution for *R. macrophyllum* (see **Fig. S22** for statewide range map). Beyond better-representing *R. macrophyllum* observations from Calflora (64) (see (C)), *Deepbiosphere* may also have learned substrate-specific relationships between species and the soil they grow on, as *Deepbiosphere* does not predict *R. macrophyllum,* as present in the red mountain serpentine outcrop (Lithic Argixerolls, white outline) (65), which matches the fact that *R. macrophyllum* is not serpentine-associated (66). **(C)** Progressive ~1 x 1, ~0.1 x 0.1, and ~0.01 x 0.01 degree zoom-ins of species range maps generated by *Maxent* at ~1km resolution for *R. macrophyllum* (see **Fig. S22** for statewide range map). **(D)** Progressive ~1 x 1, ~0.1 x 0.1, and ~0.01 x 0.01 degree zoom-ins of NAIP imagery for the same geographic extent overlaid with known species occurrences from both the *Deepbiosphere* training dataset (pink Xs) and a larger collection of contemporary and historic occurrences from *Calflora* (64) (cyan Xs).

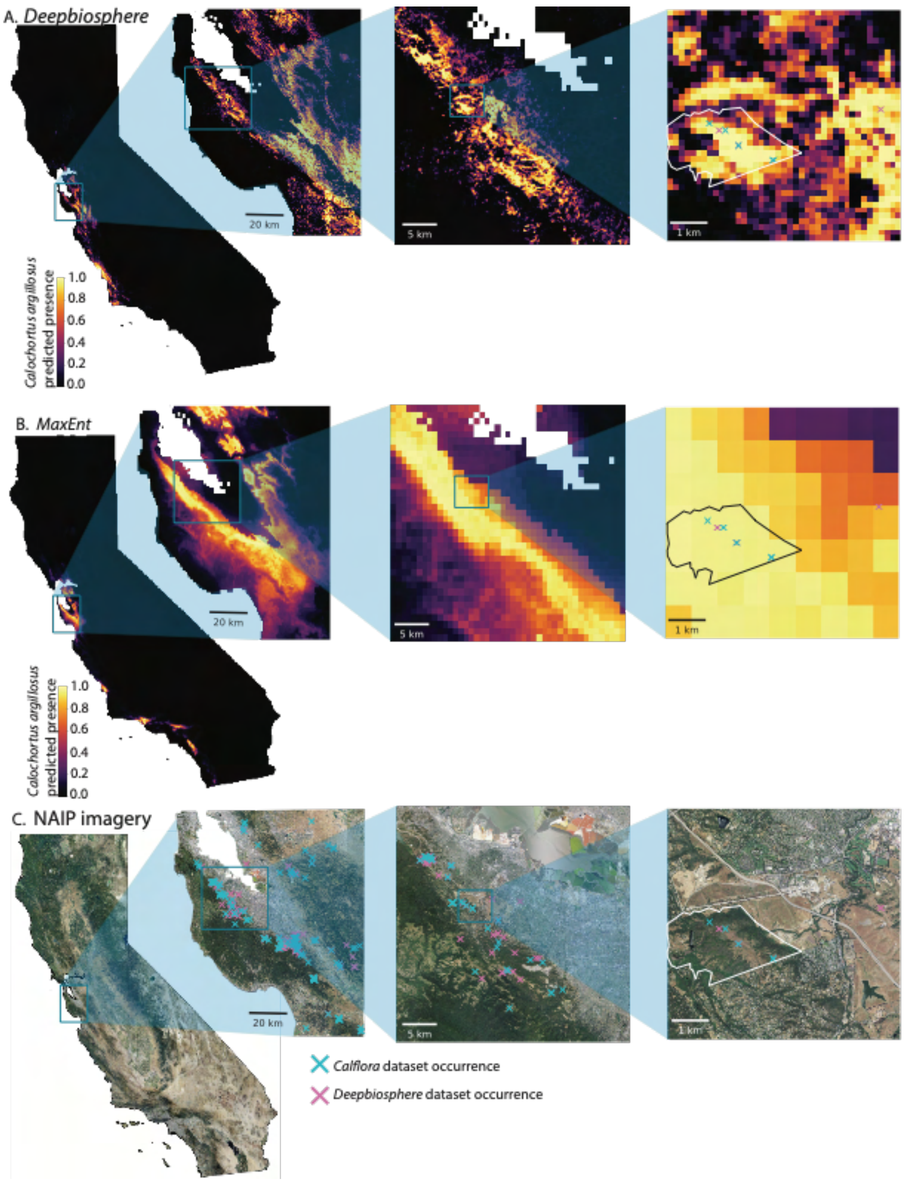

Fig. S26 | High-resolution zoom-in of *Calochortus argillosus* in the Bay Area region.
(A) Progressive ~1 x 1, ~0.1 x 0.1, and ~0.01 x 0.01 degree zoom-ins of the species range maps generated by *Deepbiosphere* at 150m resolution for *C. argillosus* (see **Fig. S22** for statewide range map). At the coarsest scale, *Deepbiosphere* visually appears to have a more tightly-resolved species distribution map compared to both training observations from the *Deepbiosphere* dataset and unseen Calflora observations (see (C)), as compared to *Maxent* (see (B)). At the finest scale, *Deepbiosphere* can finely resolve the outlines of Jasper Ridge Biological Preserve (outlined in white). **(B)** Progressive ~1 x 1, ~0.1 x 0.1, and ~0.01 x 0.01 degree zoom-ins of the species range maps generated by *Maxent* at ~1km resolution for *C. argillosus* (see **Fig. S22** for statewide range map). At the finest scale, *Maxent's* climate predictors lack the land use information present in remote sensing imagery to distinguish residential zones from protected habitat at Jasper Ridge Biological Preserve (outlined in black) **(C)** Progressive ~1 x 1, ~0.1 x 0.1, and ~0.01 x 0.01 degree zoom-ins of NAIP imagery for the same geographic extent overlaid with known species occurrences from both the *Deepbiosphere* training dataset (pink Xs) and a larger collection of contemporary and historic occurrences from *Calflora* (64) (cyan Xs).

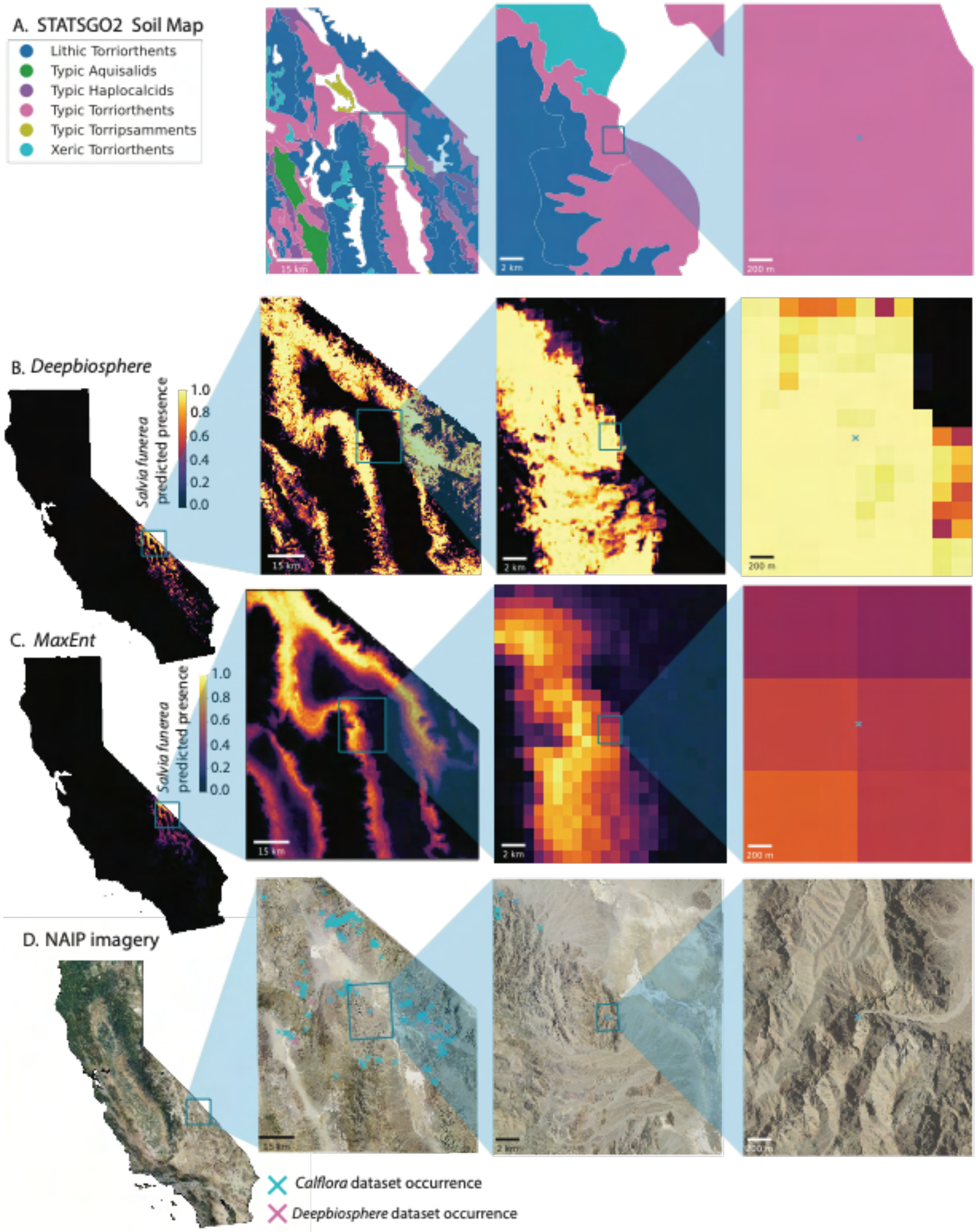

Fig. S27 | High-resolution zoom-in of *Salvia funerea* in Death Valley.
(A) Progressive ~1 x 1, ~0.1 x 0.1, and ~0.01 x 0.01 degree zoom-ins of soil maps from the USDA's STATSGO2 soil database (65). **(B)** Progressive ~1 x 1, ~0.1 x 0.1, and ~0.01 x 0.01 degree zoom-ins of the species range maps generated by *Deepbiosphere* at 150m resolution for *S. funerea* (see **Fig. S22** for statewide range map). *Deepbiosphere's* remote sensing-based predictors enable it to finely resolve *S. funerea's* distribution along the limestone canyons flanking Death Valley and finely resolve the washes from the canyon foothills in the highest-resolution scale. **(C)** Progressive ~1 x 1, ~0.1 x 0.1, and ~0.01 x 0.01 degree zoom-ins of the species range maps generated by *Maxent* at ~1km resolution for *S. funerea* (see **Fig. S22** for statewide range map). **(D)** NAIP imagery for the same geographic extent overlaid with known species occurrences from both the *Deepbiosphere* training dataset (pink Xs) and a larger collection of contemporary and historic occurrences from *Calflora* (64) (cyan Xs). Only one observation from *Calflora* is present in the example area (centered in the zoom-ins).

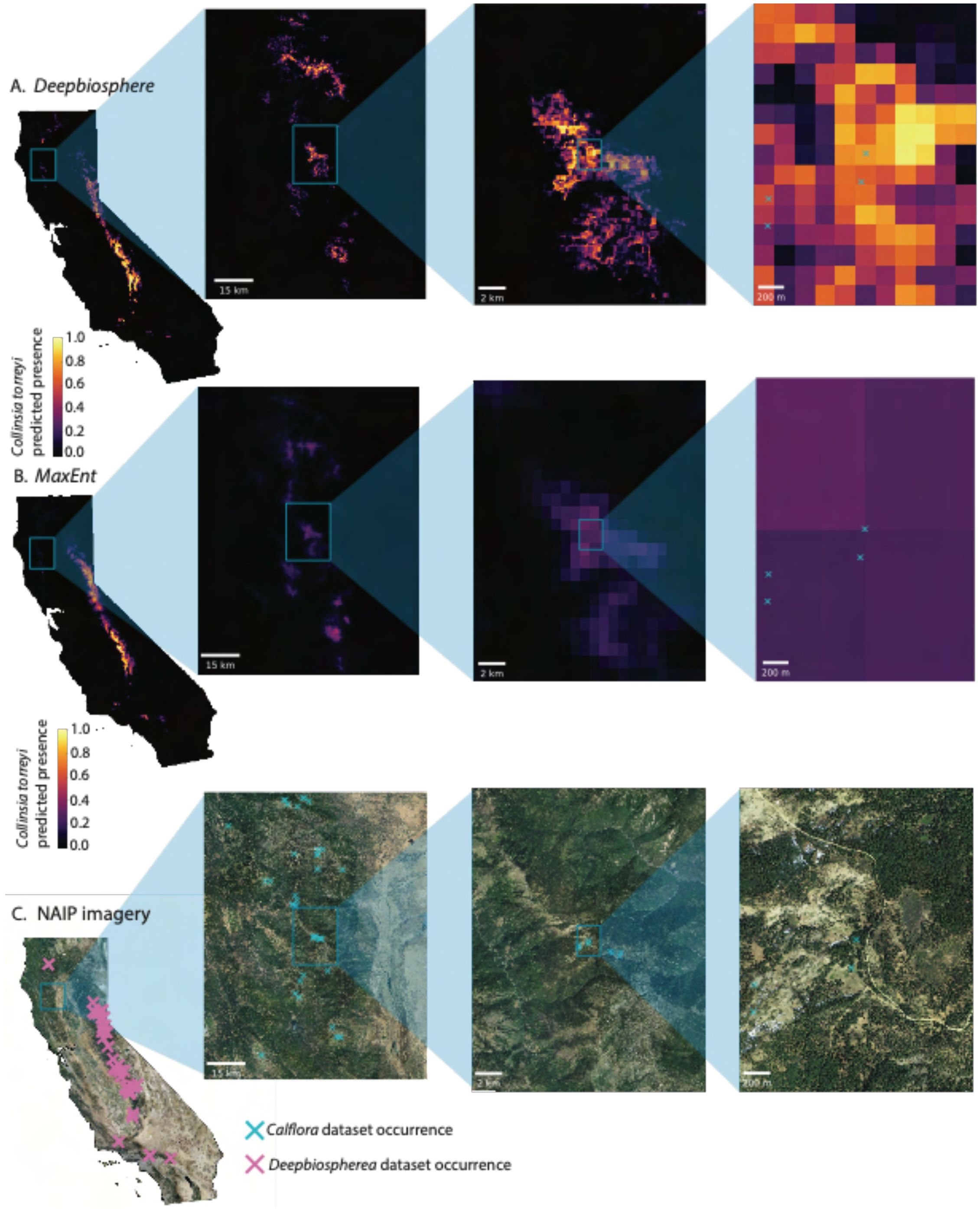

Fig. S28 | High-resolution zoom-in of *Collinsia torreyi* in the North Coast Mountains.
(A) Progressive ~1 x 1, ~0.1 x 0.1, and ~0.01 x 0.01 degree zoom-ins of the species range maps generated by *Deepbiosphere* at 150m resolution for *C. torreyi* (see **Fig. S22** for statewide range map). Since *Deepbiosphere* has access to remote sensing predictors, it is able to pick up the presence of the species in the region despite having seen no training examples from the area. **(B)** Progressive ~1 x 1, ~0.1 x 0.1, and ~0.01 x 0.01 degree zoom-ins of the species range maps generated by *Maxent* at ~1km resolution for *C. torreyi* (see **Fig. S22** for statewide range map). As *Maxent* is restricted to climate variables and no observations were present in the *Deepbiosphere* dataset from that climate, the model does not predict the region as having *C. torreyi* presence. **(C)** Progressive ~1 x 1, ~0.1 x 0.1, and ~0.01 x 0.01 degree zoom-ins of NAIP imagery for the same geographic extent overlaid with known species occurrences from both the *Deepbiosphere* training dataset (pink Xs) and a larger collection of contemporary and historic occurrences from *Calflora* (64) (cyan Xs). In the *Deepbiosphere* dataset (pink Xs), there are no observations of *C. torreyi* in the Northern Coast Mountains. However, the species does grow in the region according to the expanded *Calflora* observation list (blue Xs).

Supplemental Tables

Plant biodiversity dataset	Observation date range	1/1/2015 - 5/1/2022
	Number of unique vascular species	2,221
	Total plant diversity present	29.216%
	Number of unique genera	878
	Number of unique families	153
	Number of unique observations	652,027 images
	Number of linked observations	614,727 images
	Threshold for species inclusion	500 linked observations
	Shannon diversity index	6.825
	Gini inequality index	0.334
	Simpson index	1.000
NAIP Aerial Imagery	Spatial resolution of imagery	1 meter ground sample distance
	Bands used	Blue (428-492 nm)<br>Green (533-587 nm)<br>Red (608-662 nm)<br>Near-Infrared (883-887 nm)
	Year of observation	2012
	Number of unique images	11,095

Table S1 | Key metrics of dataset
Relevant metrics for the biodiversity dataset, including number of observations, images, and summary statistics for observations. Information is also included about the National Agriculture Imagery Program aerial imagery used for convolutional neural network training.

Layer (type:depth-idx)	Output Shape	# Params	Layers Cont'd	Output Shape Cont'd	# Params Cont'd
├─Sequential: 1-1	[65, 2048, 8, 8]	--	│ │ └─Bottleneck: 3-13	[65, 1024, 16, 16]	1,183,104
│ └─SpaceToDepthModule: 2-1	[65, 64, 64, 64]	--	│ │ └─Bottleneck: 3-14	[65, 1024, 16, 16]	1,183,104
│ └─Sequential: 2-2	[65, 64, 64, 64]	--	│ │ └─Bottleneck: 3-15	[65, 1024, 16, 16]	1,183,104
│ │ └─Conv2d: 3-1	[65, 64, 64, 64]	36,864	│ │ └─Bottleneck: 3-16	[65, 1024, 16, 16]	1,183,104
│ │ └─InPlaceABN: 3-2	[65, 64, 64, 64]	128	│ │ └─Bottleneck: 3-17	[65, 1024, 16, 16]	1,183,104
│ └─Sequential: 2-3	[65, 64, 64, 64]	--	│ │ └─Bottleneck: 3-18	[65, 1024, 16, 16]	1,183,104
│ │ └─BasicBlock: 3-3	[65, 64, 64, 64]	82,304	│ │ └─Bottleneck: 3-19	[65, 1024, 16, 16]	1,183,104
│ │ └─BasicBlock: 3-4	[65, 64, 64, 64]	82,304	│ │ └─Bottleneck: 3-20	[65, 1024, 16, 16]	1,183,104
│ │ └─BasicBlock: 3-5	[65, 64, 64, 64]	82,304	│ └─Sequential: 2-6	[65, 2048, 8, 8]	--
│ └─Sequential: 2-4	[65, 128, 32, 32]	--	│ │ └─Bottleneck: 3-21	[65, 2048, 8, 8]	6,039,552
│ │ └─BasicBlock: 3-6	[65, 128, 32, 32]	246,720	│ │ └─Bottleneck: 3-22	[65, 2048, 8, 8]	4,462,592
│ │ └─BasicBlock: 3-7	[65, 128, 32, 32]	312,000	│ │ └─Bottleneck: 3-23	[65, 2048, 8, 8]	4,462,592
│ │ └─BasicBlock: 3-8	[65, 128, 32, 32]	312,000	├─Sequential: 1-2	[65, 2048]	--
│ │ └─BasicBlock: 3-9	[65, 128, 32, 32]	312,000	│ └─FastAvgPool2d: 2-7	[65, 2048]	--
│ └─Sequential: 2-5	[65, 1024, 16, 16]	--	├─Linear: 1-3	[65, 2221]	4,550,829
│ │ └─Bottleneck: 3-10	[65, 1024, 16, 16]	1,086,848	├─Linear: 1-4	[65, 878]	1,799,022
│ │ └─Bottleneck: 3-11	[65, 1024, 16, 16]	1,183,104	├─Linear: 1-5	[65, 153]	313,497
│ │ └─Bottleneck: 3-12	[65, 1024, 16, 16]	1,183,104			
Total params: 36,012,596					
Batch size: 150 images					
Input size (MB):157.29					
Forward/backward pass size (MB) :22,343.79					
Params size (MB): 135.60					
Estimated Total Size (MB): 22,636.67					
GPU: NVIDIA P100 GPU					

Table S2 | Model summary of *TResNet* architecture
Summary of training statistics and parameters of the modified *TResNet* CNN architecture. The right-hand side columns are the continuation of the model summary. Summary generated using torchinfo version 1.7.0 (63).

Layer (type:depth-idx)	Output Shape	# Params	Layers Cont'd	Output Shape Cont'd	# Params Cont'd
├─Sequential: 1-1	[150, 2048, 8, 8]	--	│ │ └─Bottleneck: 3-22	[150, 2048, 8, 8]	4,462,592
│ └─SpaceToDepthModule: 2-1	[150, 64, 64, 64]	--	│ │ └─Bottleneck: 3-23	[150, 2048, 8, 8]	4,462,592
│ └─Sequential: 2-2	[150, 64, 64, 64]	--	├─Sequential: 1-2	[150, 2048]	--
│ │ └─Conv2d: 3-1	[150, 64, 64, 64]	36,864	│ └─FastAvgPool2d: 2-7	[150, 2048]	--
│ │ └─InPlaceABN: 3-2	[150, 64, 64, 64]	128	├─Sequential: 1-3	[150, 2048]	--
│ └─Sequential: 2-3	[150, 64, 64, 64]	--	│ └─Linear: 2-8	[150, 2048]	4,196,352
│ │ └─BasicBlock: 3-3	[150, 64, 64, 64]	82,304	│ └─BatchNorm1d: 2-9	[150, 2048]	4,096
│ │ └─BasicBlock: 3-4	[150, 64, 64, 64]	82,304	│ └─ReLU: 2-10	[150, 2048]	--
│ │ └─BasicBlock: 3-5	[150, 64, 64, 64]	82,304	├─Sequential: 1-4	[150, 2048]	--
│ └─Sequential: 2-4	[150, 128, 32, 32]	--	│ └─Linear: 2-11	[150, 1000]	20,000
│ │ └─BasicBlock: 3-6	[150, 128, 32, 32]	246,720	│ └─ELU: 2-12	[150, 1000]	--
│ │ └─BasicBlock: 3-7	[150, 128, 32, 32]	312,000	│ └─Linear: 2-13	[150, 1000]	1,001,000
│ │ └─BasicBlock: 3-8	[150, 128, 32, 32]	312,000	│ └─ELU: 2-14	[150, 1000]	--
│ │ └─BasicBlock: 3-9	[150, 128, 32, 32]	312,000	│ └─Linear: 2-15	[150, 2000]	2,002,000
│ └─Sequential: 2-5	[150, 1024, 16, 16]	--	│ └─ELU: 2-16	[150, 2000]	--
│ │ └─Bottleneck: 3-10	[150, 1024, 16, 16]	1,086,848	│ └─Dropout: 2-17	[150, 2000]	--
│ │ └─Bottleneck: 3-11	[150, 1024, 16, 16]	1,183,104	│ └─Linear: 2-18	[150, 2000]	4,002,000
│ │ └─Bottleneck: 3-12	[150, 1024, 16, 16]	1,183,104	│ └─ELU: 2-19	[150, 2000]	--
│ │ └─Bottleneck: 3-13	[150, 1024, 16, 16]	1,183,104	│ └─Linear: 2-20	[150, 2048]	4,098,048
│ │ └─Bottleneck: 3-14	[150, 1024, 16, 16]	1,183,104	│ └─BatchNorm1d: 2-21	[150, 2048]	4,096
│ │ └─Bottleneck: 3-15	[150, 1024, 16, 16]	1,183,104	│ └─ELU: 2-22	[150, 2048]	--
│ │ └─Bottleneck: 3-16	[150, 1024, 16, 16]	1,183,104	├─Sequential: 1-5	[150, 2048]	--
│ │ └─Bottleneck: 3-17	[150, 1024, 16, 16]	1,183,104	│ └─Linear: 2-23	[150, 2048]	8,390,656
│ │ └─Bottleneck: 3-18	[150, 1024, 16, 16]	1,183,104	│ └─ReLU: 2-24	[150, 2048]	--
│ │ └─Bottleneck: 3-19	[150, 1024, 16, 16]	1,183,104	├─Linear: 1-6	[150, 2221]	4,550,829
│ │ └─Bottleneck: 3-20	[150, 1024, 16, 16]	1,183,104	├─Linear: 1-7	[150, 878]	1,799,022
│ └─Sequential: 2-6	[150, 2048, 8, 8]	--	├─Linear: 1-8	[150, 153]	313,497
│ │ └─Bottleneck: 3-21	[150, 2048, 8, 8]	6,039,552			
Total params: 59,730,844					
Batch size: 150 images					
Input size (MB): 157.30					
Forward/backward pass size (MB): 22,364.51					
Params size (MB): 238.92					
Estimated Total Size (MB): 22,760.73					
GPU: NVIDIA A100					

Table S3 | Model summary of *Deepbiosphere* architecture

Summary of training statistics and parameters of the *Deepbiosphere* remote sensing + climate architecture. The right-hand side columns are the continuation of the model summary. Summary generated using torchinfo version 1.7.0 (63).

Layer (type:depth-idx)	Output Shape	# Params	Layers Cont'd	Output Shape Cont'd	# Params Cont'd
├─BasicConv2d: 1-1	[100, 32, 254, 254]	--	│ │ └─Conv2d: 3-87	[100, 192, 30, 30]	215,040
│ └─Conv2d: 2-1	[100, 32, 254, 254]	1,152	│ │ └─BatchNorm2d: 3-88	[100, 192, 30, 30]	384
│ └─BatchNorm2d: 2-2	[100, 32, 254, 254]	64	│ └─BasicConv2d: 2-55	[100, 192, 30, 30]	--
├─BasicConv2d: 1-2	[100, 32, 252, 252]	--	│ │ └─Conv2d: 3-89	[100, 192, 30, 30]	147,456
│ └─Conv2d: 2-3	[100, 32, 252, 252]	9,216	│ │ └─BatchNorm2d: 3-90	[100, 192, 30, 30]	384
│ └─BatchNorm2d: 2-4	[100, 32, 252, 252]	64	├─InceptionC: 1-14	[100, 768, 30, 30]	--
├─BasicConv2d: 1-3	[100, 64, 252, 252]	--	│ └─BasicConv2d: 2-56	[100, 192, 30, 30]	--
│ └─Conv2d: 2-5	[100, 64, 252, 252]	18,432	│ │ └─Conv2d: 3-91	[100, 192, 30, 30]	147,456
│ └─BatchNorm2d: 2-6	[100, 64, 252, 252]	128	│ │ └─BatchNorm2d: 3-92	[100, 192, 30, 30]	384
├─MaxPool2d: 1-4	[100, 64, 125, 125]	--	│ └─BasicConv2d: 2-57	[100, 160, 30, 30]	--
├─BasicConv2d: 1-5	[100, 80, 125, 125]	--	│ │ └─Conv2d: 3-93	[100, 160, 30, 30]	122,880
│ └─Conv2d: 2-7	[100, 80, 125, 125]	5,120	│ │ └─BatchNorm2d: 3-94	[100, 160, 30, 30]	320
│ └─BatchNorm2d: 2-8	[100, 80, 125, 125]	160	│ └─BasicConv2d: 2-58	[100, 160, 30, 30]	--
├─BasicConv2d: 1-6	[100, 192, 123, 123]	--	│ │ └─Conv2d: 3-95	[100, 160, 30, 30]	179,200
│ └─Conv2d: 2-9	[100, 192, 123, 123]	138,240	│ │ └─BatchNorm2d: 3-96	[100, 160, 30, 30]	320
│ └─BatchNorm2d: 2-10	[100, 192, 123, 123]	384	│ └─BasicConv2d: 2-59	[100, 192, 30, 30]	--
├─MaxPool2d: 1-7	[100, 192, 61, 61]	--	│ │ └─Conv2d: 3-97	[100, 192, 30, 30]	215,040
├─InceptionA: 1-8	[100, 256, 61, 61]	--	│ │ └─BatchNorm2d: 3-98	[100, 192, 30, 30]	384
│ └─BasicConv2d: 2-11	[100, 64, 61, 61]	--	│ └─BasicConv2d: 2-60	[100, 160, 30, 30]	--
│ │ └─Conv2d: 3-1	[100, 64, 61, 61]	12,288	│ │ └─Conv2d: 3-99	[100, 160, 30, 30]	122,880
│ │ └─BatchNorm2d: 3-2	[100, 64, 61, 61]	128	│ │ └─BatchNorm2d: 3-100	[100, 160, 30, 30]	320
│ └─BasicConv2d: 2-12	[100, 48, 61, 61]	--	│ └─BasicConv2d: 2-61	[100, 160, 30, 30]	--
│ │ └─Conv2d: 3-3	[100, 48, 61, 61]	9,216	│ │ └─Conv2d: 3-101	[100, 160, 30, 30]	179,200
│ │ └─BatchNorm2d: 3-4	[100, 48, 61, 61]	96	│ │ └─BatchNorm2d: 3-102	[100, 160, 30, 30]	320
│ └─BasicConv2d: 2-13	[100, 64, 61, 61]	--	│ └─BasicConv2d: 2-62	[100, 160, 30, 30]	--
│ │ └─Conv2d: 3-5	[100, 64, 61, 61]	76,800	│ │ └─Conv2d: 3-103	[100, 160, 30, 30]	179,200
│ │ └─BatchNorm2d: 3-6	[100, 64, 61, 61]	128	│ │ └─BatchNorm2d: 3-104	[100, 160, 30, 30]	320
│ └─BasicConv2d: 2-14	[100, 64, 61, 61]	--	│ └─BasicConv2d: 2-63	[100, 160, 30, 30]	--
│ │ └─Conv2d: 3-7	[100, 64, 61, 61]	12,288	│ │ └─Conv2d: 3-105	[100, 160, 30, 30]	179,200
│ │ └─BatchNorm2d: 3-8	[100, 64, 61, 61]	128	│ │ └─BatchNorm2d: 3-106	[100, 160, 30, 30]	320
│ └─BasicConv2d: 2-15	[100, 96, 61, 61]	--	│ └─BasicConv2d: 2-64	[100, 192, 30, 30]	--
│ │ └─Conv2d: 3-9	[100, 96, 61, 61]	55,296	│ │ └─Conv2d: 3-107	[100, 192, 30, 30]	215,040
│ │ └─BatchNorm2d: 3-10	[100, 96, 61, 61]	192	│ │ └─BatchNorm2d: 3-108	[100, 192, 30, 30]	384
│ └─BasicConv2d: 2-16	[100, 96, 61, 61]	--	│ └─BasicConv2d: 2-65	[100, 192, 30, 30]	--
│ │ └─Conv2d: 3-11	[100, 96, 61, 61]	82,944	│ │ └─Conv2d: 3-109	[100, 192, 30, 30]	147,456
│ │ └─BatchNorm2d: 3-12	[100, 96, 61, 61]	192	│ │ └─BatchNorm2d: 3-110	[100, 192, 30, 30]	384
│ └─BasicConv2d: 2-17	[100, 32, 61, 61]	--	├─InceptionC: 1-15	[100, 768, 30, 30]	--
│ │ └─Conv2d: 3-13	[100, 32, 61, 61]	6,144	│ └─BasicConv2d: 2-66	[100, 192, 30, 30]	--
│ │ └─BatchNorm2d: 3-14	[100, 32, 61, 61]	64	│ │ └─Conv2d: 3-111	[100, 192, 30, 30]	147,456
├─InceptionA: 1-9	[100, 288, 61, 61]	--	│ │ └─BatchNorm2d: 3-112	[100, 192, 30, 30]	384
│ └─BasicConv2d: 2-18	[100, 64, 61, 61]	--	│ └─BasicConv2d: 2-67	[100, 192, 30, 30]	--
│ │ └─Conv2d: 3-15	[100, 64, 61, 61]	16,384	│ │ └─Conv2d: 3-113	[100, 192, 30, 30]	147,456
│ │ └─BatchNorm2d: 3-16	[100, 64, 61, 61]	128	│ │ └─BatchNorm2d: 3-114	[100, 192, 30, 30]	384
│ └─BasicConv2d: 2-19	[100, 48, 61, 61]	--	│ └─BasicConv2d: 2-68	[100, 192, 30, 30]	--
│ │ └─Conv2d: 3-17	[100, 48, 61, 61]	12,288	│ │ └─Conv2d: 3-115	[100, 192, 30, 30]	258,048
│ │ └─BatchNorm2d: 3-18	[100, 48, 61, 61]	96	│ │ └─BatchNorm2d: 3-116	[100, 192, 30, 30]	384
│ └─BasicConv2d: 2-20	[100, 64, 61, 61]	--	│ └─BasicConv2d: 2-69	[100, 192, 30, 30]	--
│ │ └─Conv2d: 3-19	[100, 64, 61, 61]	76,800	│ │ └─Conv2d: 3-117	[100, 192, 30, 30]	258,048
│ │ └─BatchNorm2d: 3-20	[100, 64, 61, 61]	128	│ │ └─BatchNorm2d: 3-118	[100, 192, 30, 30]	384
│ └─BasicConv2d: 2-21	[100, 64, 61, 61]	--	│ └─BasicConv2d: 2-70	[100, 192, 30, 30]	--
│ │ └─Conv2d: 3-21	[100, 64, 61, 61]	16,384	│ │ └─Conv2d: 3-119	[100, 192, 30, 30]	147,456
│ │ └─BatchNorm2d: 3-22	[100, 64, 61, 61]	128	│ │ └─BatchNorm2d: 3-120	[100, 192, 30, 30]	384
│ └─BasicConv2d: 2-22	[100, 96, 61, 61]	--	│ └─BasicConv2d: 2-71	[100, 192, 30, 30]	--
│ │ └─Conv2d: 3-23	[100, 96, 61, 61]	55,296	│ │ └─Conv2d: 3-121	[100, 192, 30, 30]	258,048
│ │ └─BatchNorm2d: 3-24	[100, 96, 61, 61]	192	│ │ └─BatchNorm2d: 3-122	[100, 192, 30, 30]	384
│ └─BasicConv2d: 2-23	[100, 96, 61, 61]	--	│ └─BasicConv2d: 2-72	[100, 192, 30, 30]	--
│ │ └─Conv2d: 3-25	[100, 96, 61, 61]	82,944	│ │ └─Conv2d: 3-123	[100, 192, 30, 30]	258,048
│ │ └─BatchNorm2d: 3-26	[100, 96, 61, 61]	192	│ │ └─BatchNorm2d: 3-124	[100, 192, 30, 30]	384
│ └─BasicConv2d: 2-24	[100, 64, 61, 61]	--	│ └─BasicConv2d: 2-73	[100, 192, 30, 30]	--
│ │ └─Conv2d: 3-27	[100, 64, 61, 61]	16,384	│ │ └─Conv2d: 3-125	[100, 192, 30, 30]	258,048

│ │ └─BatchNorm2d: 3-28	[100, 64, 61, 61]	128
├─InceptionA: 1-10	[100, 288, 61, 61]	--
│ └─BasicConv2d: 2-25	[100, 64, 61, 61]	--
│ │ └─Conv2d: 3-29	[100, 64, 61, 61]	18,432
│ │ └─BatchNorm2d: 3-30	[100, 64, 61, 61]	128
│ └─BasicConv2d: 2-26	[100, 48, 61, 61]	--
│ │ └─Conv2d: 3-31	[100, 48, 61, 61]	13,824
│ │ └─BatchNorm2d: 3-32	[100, 48, 61, 61]	96
│ └─BasicConv2d: 2-27	[100, 64, 61, 61]	--
│ │ └─Conv2d: 3-33	[100, 64, 61, 61]	76,800
│ │ └─BatchNorm2d: 3-34	[100, 64, 61, 61]	128
│ └─BasicConv2d: 2-28	[100, 64, 61, 61]	--
│ │ └─Conv2d: 3-35	[100, 64, 61, 61]	18,432
│ │ └─BatchNorm2d: 3-36	[100, 64, 61, 61]	128
│ └─BasicConv2d: 2-29	[100, 96, 61, 61]	--
│ │ └─Conv2d: 3-37	[100, 96, 61, 61]	55,296
│ │ └─BatchNorm2d: 3-38	[100, 96, 61, 61]	192
│ └─BasicConv2d: 2-30	[100, 96, 61, 61]	--
│ │ └─Conv2d: 3-39	[100, 96, 61, 61]	82,944
│ │ └─BatchNorm2d: 3-40	[100, 96, 61, 61]	192
│ └─BasicConv2d: 2-31	[100, 64, 61, 61]	--
│ │ └─Conv2d: 3-41	[100, 64, 61, 61]	18,432
│ │ └─BatchNorm2d: 3-42	[100, 64, 61, 61]	128
├─InceptionB: 1-11	[100, 768, 30, 30]	--
│ └─BasicConv2d: 2-32	[100, 384, 30, 30]	--
│ │ └─Conv2d: 3-43	[100, 384, 30, 30]	995,328
│ │ └─BatchNorm2d: 3-44	[100, 384, 30, 30]	768
│ └─BasicConv2d: 2-33	[100, 64, 61, 61]	--
│ │ └─Conv2d: 3-45	[100, 64, 61, 61]	18,432
│ │ └─BatchNorm2d: 3-46	[100, 64, 61, 61]	128
│ └─BasicConv2d: 2-34	[100, 96, 61, 61]	--
│ │ └─Conv2d: 3-47	[100, 96, 61, 61]	55,296
│ │ └─BatchNorm2d: 3-48	[100, 96, 61, 61]	192
│ └─BasicConv2d: 2-35	[100, 96, 30, 30]	--
│ │ └─Conv2d: 3-49	[100, 96, 30, 30]	82,944
│ │ └─BatchNorm2d: 3-50	[100, 96, 30, 30]	192
├─InceptionC: 1-12	[100, 768, 30, 30]	--
│ └─BasicConv2d: 2-36	[100, 192, 30, 30]	--
│ │ └─Conv2d: 3-51	[100, 192, 30, 30]	147,456
│ │ └─BatchNorm2d: 3-52	[100, 192, 30, 30]	384
│ └─BasicConv2d: 2-37	[100, 128, 30, 30]	--
│ │ └─Conv2d: 3-53	[100, 128, 30, 30]	98,304
│ │ └─BatchNorm2d: 3-54	[100, 128, 30, 30]	256
│ └─BasicConv2d: 2-38	[100, 128, 30, 30]	--
│ │ └─Conv2d: 3-55	[100, 128, 30, 30]	114,688
│ │ └─BatchNorm2d: 3-56	[100, 128, 30, 30]	256
│ └─BasicConv2d: 2-39	[100, 192, 30, 30]	--
│ │ └─Conv2d: 3-57	[100, 192, 30, 30]	172,032
│ │ └─BatchNorm2d: 3-58	[100, 192, 30, 30]	384
│ └─BasicConv2d: 2-40	[100, 128, 30, 30]	--
│ │ └─Conv2d: 3-59	[100, 128, 30, 30]	98,304
│ │ └─BatchNorm2d: 3-60	[100, 128, 30, 30]	256
│ └─BasicConv2d: 2-41	[100, 128, 30, 30]	--
│ │ └─Conv2d: 3-61	[100, 128, 30, 30]	114,688
│ │ └─BatchNorm2d: 3-62	[100, 128, 30, 30]	256
│ └─BasicConv2d: 2-42	[100, 128, 30, 30]	--
│ │ └─Conv2d: 3-63	[100, 128, 30, 30]	114,688
│ │ └─BatchNorm2d: 3-64	[100, 128, 30, 30]	256
│ └─BasicConv2d: 2-43	[100, 128, 30, 30]	--
│ │ └─Conv2d: 3-65	[100, 128, 30, 30]	114,688
│ │ └─BatchNorm2d: 3-66	[100, 128, 30, 30]	256
│ │ └─BatchNorm2d: 3-126	[100, 192, 30, 30]	384
│ └─BasicConv2d: 2-74	[100, 192, 30, 30]	--
│ │ └─Conv2d: 3-127	[100, 192, 30, 30]	258,048
│ │ └─BatchNorm2d: 3-128	[100, 192, 30, 30]	384
│ └─BasicConv2d: 2-75	[100, 192, 30, 30]	--
│ │ └─Conv2d: 3-129	[100, 192, 30, 30]	147,456
│ │ └─BatchNorm2d: 3-130	[100, 192, 30, 30]	384
├─InceptionAux: 1-16	--	--
│ └─BasicConv2d: 2-76	--	--
│ │ └─Conv2d: 3-131	--	98,304
│ │ └─BatchNorm2d: 3-132	--	256
│ └─BasicConv2d: 2-77	--	--
│ │ └─Conv2d: 3-133	--	2,457,600
│ │ └─BatchNorm2d: 3-134	--	1,536
│ └─Linear: 2-78	--	1,707,949
├─InceptionD: 1-17	[100, 1280, 14, 14]	--
│ └─BasicConv2d: 2-79	[100, 192, 30, 30]	--
│ │ └─Conv2d: 3-135	[100, 192, 30, 30]	147,456
│ │ └─BatchNorm2d: 3-136	[100, 192, 30, 30]	384
│ └─BasicConv2d: 2-80	[100, 320, 14, 14]	--
│ │ └─Conv2d: 3-137	[100, 320, 14, 14]	552,960
│ │ └─BatchNorm2d: 3-138	[100, 320, 14, 14]	640
│ └─BasicConv2d: 2-81	[100, 192, 30, 30]	--
│ │ └─Conv2d: 3-139	[100, 192, 30, 30]	147,456
│ │ └─BatchNorm2d: 3-140	[100, 192, 30, 30]	384
│ └─BasicConv2d: 2-82	[100, 192, 30, 30]	--
│ │ └─Conv2d: 3-141	[100, 192, 30, 30]	258,048
│ │ └─BatchNorm2d: 3-142	[100, 192, 30, 30]	384
│ └─BasicConv2d: 2-83	[100, 192, 30, 30]	--
│ │ └─Conv2d: 3-143	[100, 192, 30, 30]	258,048
│ │ └─BatchNorm2d: 3-144	[100, 192, 30, 30]	384
│ └─BasicConv2d: 2-84	[100, 192, 14, 14]	--
│ │ └─Conv2d: 3-145	[100, 192, 14, 14]	331,776
│ │ └─BatchNorm2d: 3-146	[100, 192, 14, 14]	384
├─InceptionE: 1-18	[100, 2048, 14, 14]	--
│ └─BasicConv2d: 2-85	[100, 320, 14, 14]	--
│ │ └─Conv2d: 3-147	[100, 320, 14, 14]	409,600
│ │ └─BatchNorm2d: 3-148	[100, 320, 14, 14]	640
│ └─BasicConv2d: 2-86	[100, 384, 14, 14]	--
│ │ └─Conv2d: 3-149	[100, 384, 14, 14]	491,520
│ │ └─BatchNorm2d: 3-150	[100, 384, 14, 14]	768
│ └─BasicConv2d: 2-87	[100, 384, 14, 14]	--
│ │ └─Conv2d: 3-151	[100, 384, 14, 14]	442,368
│ │ └─BatchNorm2d: 3-152	[100, 384, 14, 14]	768
│ └─BasicConv2d: 2-88	[100, 384, 14, 14]	--
│ │ └─Conv2d: 3-153	[100, 384, 14, 14]	442,368
│ │ └─BatchNorm2d: 3-154	[100, 384, 14, 14]	768
│ └─BasicConv2d: 2-89	[100, 448, 14, 14]	--
│ │ └─Conv2d: 3-155	[100, 448, 14, 14]	573,440
│ │ └─BatchNorm2d: 3-156	[100, 448, 14, 14]	896
│ └─BasicConv2d: 2-90	[100, 384, 14, 14]	--
│ │ └─Conv2d: 3-157	[100, 384, 14, 14]	1,548,288
│ │ └─BatchNorm2d: 3-158	[100, 384, 14, 14]	768
│ └─BasicConv2d: 2-91	[100, 384, 14, 14]	--
│ │ └─Conv2d: 3-159	[100, 384, 14, 14]	442,368
│ │ └─BatchNorm2d: 3-160	[100, 384, 14, 14]	768
│ └─BasicConv2d: 2-92	[100, 384, 14, 14]	--
│ │ └─Conv2d: 3-161	[100, 384, 14, 14]	442,368
│ │ └─BatchNorm2d: 3-162	[100, 384, 14, 14]	768
│ └─BasicConv2d: 2-93	[100, 192, 14, 14]	--
│ │ └─Conv2d: 3-163	[100, 192, 14, 14]	245,760

│ └─BasicConv2d: 2-44	[100, 192, 30, 30]	--	│ │ └─BatchNorm2d: 3-164	[100, 192, 14, 14]	384
│ │ └─Conv2d: 3-67	[100, 192, 30, 30]	172,032	├─InceptionE: 1-19	[100, 2048, 14, 14]	--
│ │ └─BatchNorm2d: 3-68	[100, 192, 30, 30]	384	│ └─BasicConv2d: 2-94	[100, 320, 14, 14]	--
│ └─BasicConv2d: 2-45	[100, 192, 30, 30]	--	│ │ └─Conv2d: 3-165	[100, 320, 14, 14]	655,360
│ │ └─Conv2d: 3-69	[100, 192, 30, 30]	147,456	│ │ └─BatchNorm2d: 3-166	[100, 320, 14, 14]	640
│ │ └─BatchNorm2d: 3-70	[100, 192, 30, 30]	384	│ └─BasicConv2d: 2-95	[100, 384, 14, 14]	--
├─InceptionC: 1-13	[100, 768, 30, 30]	--	│ │ └─Conv2d: 3-167	[100, 384, 14, 14]	786,432
│ └─BasicConv2d: 2-46	[100, 192, 30, 30]	--	│ │ └─BatchNorm2d: 3-168	[100, 384, 14, 14]	768
│ │ └─Conv2d: 3-71	[100, 192, 30, 30]	147,456	│ └─BasicConv2d: 2-96	[100, 384, 14, 14]	--
│ │ └─BatchNorm2d: 3-72	[100, 192, 30, 30]	384	│ │ └─Conv2d: 3-169	[100, 384, 14, 14]	442,368
│ └─BasicConv2d: 2-47	[100, 160, 30, 30]	--	│ │ └─BatchNorm2d: 3-170	[100, 384, 14, 14]	768
│ │ └─Conv2d: 3-73	[100, 160, 30, 30]	122,880	│ └─BasicConv2d: 2-97	[100, 384, 14, 14]	--
│ │ └─BatchNorm2d: 3-74	[100, 160, 30, 30]	320	│ │ └─Conv2d: 3-171	[100, 384, 14, 14]	442,368
│ └─BasicConv2d: 2-48	[100, 160, 30, 30]	--	│ │ └─BatchNorm2d: 3-172	[100, 384, 14, 14]	768
│ │ └─Conv2d: 3-75	[100, 160, 30, 30]	179,200	│ └─BasicConv2d: 2-98	[100, 448, 14, 14]	--
│ │ └─BatchNorm2d: 3-76	[100, 160, 30, 30]	320	│ │ └─Conv2d: 3-173	[100, 448, 14, 14]	917,504
│ └─BasicConv2d: 2-49	[100, 192, 30, 30]	--	│ │ └─BatchNorm2d: 3-174	[100, 448, 14, 14]	896
│ │ └─Conv2d: 3-77	[100, 192, 30, 30]	215,040	│ └─BasicConv2d: 2-99	[100, 384, 14, 14]	--
│ │ └─BatchNorm2d: 3-78	[100, 192, 30, 30]	384	│ │ └─Conv2d: 3-175	[100, 384, 14, 14]	1,548,288
│ └─BasicConv2d: 2-50	[100, 160, 30, 30]	--	│ │ └─BatchNorm2d: 3-176	[100, 384, 14, 14]	768
│ │ └─Conv2d: 3-79	[100, 160, 30, 30]	122,880	│ └─BasicConv2d: 2-100	[100, 384, 14, 14]	--
│ │ └─BatchNorm2d: 3-80	[100, 160, 30, 30]	320	│ │ └─Conv2d: 3-177	[100, 384, 14, 14]	442,368
│ └─BasicConv2d: 2-51	[100, 160, 30, 30]	--	│ │ └─BatchNorm2d: 3-178	[100, 384, 14, 14]	768
│ │ └─Conv2d: 3-81	[100, 160, 30, 30]	179,200	│ └─BasicConv2d: 2-101	[100, 384, 14, 14]	--
│ │ └─BatchNorm2d: 3-82	[100, 160, 30, 30]	320	│ │ └─Conv2d: 3-179	[100, 384, 14, 14]	442,368
│ └─BasicConv2d: 2-52	[100, 160, 30, 30]	--	│ │ └─BatchNorm2d: 3-180	[100, 384, 14, 14]	768
│ │ └─Conv2d: 3-83	[100, 160, 30, 30]	179,200	│ └─BasicConv2d: 2-102	[100, 192, 14, 14]	--
│ │ └─BatchNorm2d: 3-84	[100, 160, 30, 30]	320	│ │ └─Conv2d: 3-181	[100, 192, 14, 14]	393,216
│ └─BasicConv2d: 2-53	[100, 160, 30, 30]	--	│ │ └─BatchNorm2d: 3-182	[100, 192, 14, 14]	384
│ │ └─Conv2d: 3-85	[100, 160, 30, 30]	179,200	├─AdaptiveAvgPool2d: 1-20	[100, 2048, 1, 1]	--
│ │ └─BatchNorm2d: 3-86	[100, 160, 30, 30]	320	├─Dropout: 1-21	[100, 2048, 1, 1]	--
│ └─BasicConv2d: 2-54	[100, 192, 30, 30]	--	├─Linear: 1-22	[100, 2221]	4,550,829

Total params: 30,602,330

Batch size: 100 images

Input size (MB): 104.86

Forward/backward pass size (MB): 43,286.00

Params size (MB): 122.41

Estimated Total Size (MB): 43,513.27

GPU: NVIDIA A100

Table S4 | Model summary of *Inception V3* architecture

Summary of training statistics and parameters of the *Inception V3* architecture baseline from ref. (42). The right-hand side columns are the continuation of the model summary. Summary generated using torchinfo version 1.7.0 (63).

Layer (type:depth-idx)	Output Shape	# Params
Bioclim_MLP	[1000, 2221]	--
├─Sequential: 1-1	[1000, 2000]	--
│ └─Linear: 2-1	[1000, 1000]	20,000
│ └─ELU: 2-2	[1000, 1000]	--
│ └─Linear: 2-3	[1000, 1000]	1,001,000
│ └─ELU: 2-4	[1000, 1000]	--
│ └─Linear: 2-5	[1000, 2000]	2,002,000
│ └─ELU: 2-6	[1000, 2000]	--
│ └─Dropout: 2-7	[1000, 2000]	--
│ └─Linear: 2-8	[1000, 2000]	4,002,000
│ └─ELU: 2-9	[1000, 2000]	--
├─Linear: 1-2	[1000, 153]	306,153
├─Linear: 1-3	[1000, 878]	1,756,878
├─Linear: 1-4	[1000, 2221]	4,444,221
Total params: 13,532,252		
Batch size: 1,000		
Input size (MB): 0.08		
Forward/backward pass size (MB): 65.77		
Params size (MB): 45.88		
Estimated Total Size (MB): 111.72		
GPU: NVIDIA GRID M60-8Q		

Table S5 | Model summary of *Bioclim MLP* architecture
Summary of training statistics and parameters of the *Bioclim MLP* architecture inspired by ref (50). Summary generated using torchinfo version 1.7.0 (63).

model	loss	LR	BS	epoch	Precision per-species	Precision per-image	Recall per-species	Recall per-image
+ *taxonomy* + *nearby species*	Sampling-aware BCE	$1x10^{-5}$	150	12	**0.01 [0.0025-0.025]**	**0.0091 [0.0044-0.0238]**	0.8958 [0.4667-1.0]	**1.0 [0.8286-1.0]**
+ *taxonomy* - *nearby species*	Sampling-aware BCE	$1x10^{-5}$	150	11	0.009 [0.0023-0.0224]	0.0082 [0.004-0.0216]	**0.9062 [0.4762-1.0]**	**1.0 [0.825-1.0]**
- *taxonomy* + *nearby species*	Sampling-aware BCE	$1x10^{-5}$	150	13	**0.01 [0.0027-0.0252]**	0.0089 [0.0043-0.0227]	0.9028 [0.5-1.0]	**1.0 [0.8333-1.0]**

model cont'd	loss	LR	BS	epoch	F1 per-species	F1 per-image	mAP	Presence accuracy
+ *taxonomy* + *nearby species*	Sampling-aware BCE	$1x10^{-5}$	150	12	**0.0198 [0.0051-0.0479]**	**0.018 [0.0088-0.0463]**	**0.1426**	0.8645
+ *taxonomy* - *nearby species*	Sampling-aware BCE	$1x10^{-5}$	150	11	0.0178 [0.0046-0.0435]	0.0162 [0.008-0.0423]	0.1378	0.863
- *taxonomy* + *nearby species*	Sampling-aware BCE	$1x10^{-5}$	150	13	0.0195 [0.0054-0.0491]	0.0177 [0.0085-0.0444]	0.1420	**0.8673**

model cont'd	loss	LR	BS	epoch	Top 1 per-image	Top 5 per-image	Top 30 per-image	Top 100 per-image
+ *taxonomy* + *nearby species*	Sampling-aware BCE	$1x10^{-5}$	150	12	**0.0366**	**0.1281**	**0.3975**	**0.6779**
+ *taxonomy* - *nearby species*	Sampling-aware BCE	$1x10^{-5}$	150	11	0.0362	0.1183	0.3752	0.6509
- *taxonomy* + *nearby species*	Sampling-aware BCE	$1x10^{-5}$	150	13	0.0348	0.1241	0.3896	0.6772

model cont'd	loss	LR	BS	epoch	Top 1 per-species	Top 5 per-species	Top 30 per-species	Top 100 per-species
+ *taxonomy* + *nearby species*	Sampling-aware BCE	$1x10^{-5}$	150	12	0.0 [0.0-0.0]	0.0 [0.0-0.0]	0.0 [0.0-0.3889]	**0.5 [0.0-0.8]**
+ *taxonomy* - *nearby species*	Sampling-aware BCE	$1x10^{-5}$	150	11	0.0 [0.0-0.0]	0.0 [0.0-0.0]	0.0 [0.0-0.3158]	0.4 [0.0-0.7647]
- *taxonomy* + *nearby species*	Sampling-aware BCE	$1x10^{-5}$	150	13	0.0 [0.0-0.0]	0.0 [0.0-0.0]	0.0 [0.0-0.3478]	**0.5 [0.0-0.8]**

model cont'd	loss	LR	BS	epoch	AUC_{ROC}	AUC_{PRC}	calibrated AUC_{ROC}	calibrated AUC_{PRC}
+ *taxonomy* + *nearby species*	Sampling-aware BCE	$1x10^{-5}$	150	12	**0.9268 [0.8627-0.9671]**	**0.0265 [0.0072-0.0787]**	**0.9247 [0.8579-0.965]**	**0.0224 [0.0072-0.0588]**
+ *taxonomy* - *nearby species*	Sampling-aware BCE	$1x10^{-5}$	150	11	0.923 [0.853-0.9639]	0.0224 [0.0065-0.0678]	0.9212 [0.8512-0.9617]	0.0193 [0.0063-0.0558]
- *taxonomy* + *nearby species*	Sampling-aware BCE	$1x10^{-5}$	150	13	0.9265 [0.858-0.9665]	0.0262 [0.0072-0.0767]	0.9213 [0.8533-0.9634]	0.0222 [0.0071-0.056]

Table S6 | Including species co-occurrence and taxonomic information improves species-level prediction
Ablation comparison for including co-occurring species and higher taxonomic information during model training. Using the modified *TResNet* architecture (**Table S2**), when co-occurring species information (+ *nearby species*) and when higher taxonomic information (+ *taxonomy*) is provided during training, model performance is higher on 13/20 metrics (bolded entries), indicating that both species co-occurrence signal (from the nearby species) and phylogenetic signal (from the higher taxonomic information) provides increased predictive power for species distribution modeling. Accuracies are reported for held-out observations from the uniform split of the dataset (**Fig. S4A)**. Reported statistics are median [1st quartile - 3rd quartile] calculated for the epoch of evaluation determined using early stopping from AUC_{ROC}. Abbreviations: LR = learning rate; BS = batch size; mAP = mean average precision; ROC = receiver operating characteristic curve; AUC = area under the curve; PRC = precision-recall curve; Sampling-aware BCE = sampling-aware binary cross-entropy loss.

model	loss	LR	BS	epoch	Precision per-species	Precision per-image	Recall per-species	Recall per-image
TResNet	Sampling-aware BCE	$1x10^{-5}$	150	12	**0.01 [0.0025-0.025]**	**0.0091 [0.0044-0.0238]**	**0.8958 [0.4667-1.0]**	**1.0 [0.8286-1.0]**
TResNet	CE	$1x10^{-5}$	150	12	0.0 [0.0-0.0]	0.0 [0.0-0.0]	0.0 [0.0-0.0]	0.0 [0.0-0.0]
TResNet	BCE	$1x10^{-5}$	150	13	0.0 [0.0-0.0]	0.0 [0.0-0.0]	0.0 [0.0-0.0]	0.0 [0.0-0.0]
TResNet	ASL	$1x10^{-5}$	150	10	0.0 [0.0-0.0337]	0.0 [0.0-0.0833]	0.0 [0.0-0.0549]	0.0 [0.0-0.2]

model cont'd	loss	LR	BS	epoch	F1 per-species	F1 per-image	mAP	Presence accuracy
TResNet	Sampling-aware BCE	$1x10^{-5}$	150	12	**0.0198 [0.0051-0.0479]**	**0.018 [0.0088-0.0463]**	0.1426	**0.8645**
TResNet	CE	$1x10^{-5}$	150	12	0.0 [0.0-0.0]	0.0 [0.0-0.0]	**0.145**	0
TResNet	BCE	$1x10^{-5}$	150	13	0.0 [0.0-0.0]	0.0 [0.0-0.0]	0.1255	0.0116
TResNet	ASL	$1x10^{-5}$	150	10	0.0 [0.0-0.0396]	0.0 [0.0-0.1111]	0.1217	0.1533

model cont'd	loss	LR	BS	epoch	Top 1 per-image	Top 5 per-image	Top 30 per-image	Top 100 per-image
TResNet	Sampling-aware BCE	$1x10^{-5}$	150	12	0.0366	0.1281	**0.3975**	**0.6779**
TResNet	CE	$1x10^{-5}$	150	12	**0.0391**	**0.1306**	0.3938	0.6682
TResNet	BCE	$1x10^{-5}$	150	13	0.0305	0.1042	0.3357	0.602
TResNet	ASL	$1x10^{-5}$	150	10	0.0289	0.0981	0.3202	0.5824

model cont'd	loss	LR	BS	epoch	Top 1 per-species	Top 5 per-species	Top 30 per-species	Top 100 per-species
TResNet	Sampling-aware BCE	$1x10^{-5}$	150	12	0.0 [0.0-0.0]	0.0 [0.0-0.0]	0.0 [0.0-0.3889]	**0.5 [0.0-0.8]**
TResNet	CE	$1x10^{-5}$	150	12	0.0 [0.0-0.0]	0.0 [0.0-0.0]	0.0 [0.0-0.3571]	0.4706 [0.0-0.8]
TResNet	BCE	$1x10^{-5}$	150	13	0.0 [0.0-0.0]	0.0 [0.0-0.0]	0.0 [0.0-0.2667]	0.3333 [0.0-0.6923]
TResNet	ASL	$1x10^{-5}$	150	10	0.0 [0.0-0.0]	0.0 [0.0-0.0]	0.0 [0.0-0.2857]	0.3333 [0.0-0.6667]

model cont'd	loss	LR	BS	epoch	AUC_{ROC}	AUC_{PRC}	calibrated AUC_{ROC}	calibrated AUC_{PRC}
TResNet	Sampling-aware BCE	$1x10^{-5}$	150	12	**0.9268 [0.8627-0.9671]**	**0.0265 [0.0072-0.0787]**	**0.9247 [0.8579-0.965]**	**0.0224 [0.0072-0.0588]**
TResNet	CE	$1x10^{-5}$	150	12	0.9248 [0.8543-0.966]	0.0241 [0.0069-0.0703]	0.5 [0.5-0.5]	0.0011 [0.0004-0.0088]
TResNet	BCE	$1x10^{-5}$	150	13	0.907 [0.828-0.9589]	0.0194 [0.0046-0.0696]	0.6586 [0.4991-0.8485]	0.0129 [0.0009-0.059]
TResNet	ASL	$1x10^{-5}$	150	10	0.9056 [0.8142-0.9577]	0.0192 [0.0041-0.0728]	0.9039 [0.8131-0.9546]	0.0182 [0.0043-0.0664]

Table S7 | Comparing the performance of different loss functions on uniform data split

Comparison of a variety of common loss functions to novel sampling-aware binary cross-entropy loss function. The remote sensing-only modified *TResNet* trained with our novel sampling-aware loss had the highest accuracy on 14/20 metrics, while the remote sensing-only modified *TResNet* trained with the classic single-label cross-entropy (CE) loss had the highest accuracy on 3/20 metrics. While the CE loss performed slightly better on ranking metrics than our sampling-aware loss, it had very low performance on the binary accuracy metrics, a consequence of the single-label target loss which optimizes the model to predict only one species at a time. Accuracies are reported for held-out observations from the uniform split of the dataset (**Fig. S4A)**. Reported statistics are median [1st quartile - 3rd quartile] calculated for the epoch of evaluation determined using early stopping from AUC_{ROC}. Abbreviations: LR = learning rate; BS = batch size; mAP = mean average precision; ROC = receiver operating characteristic curve; AUC = area under the curve; PRC = precision-recall curve; Sampling-aware BCE = Sampling-aware binary cross-entropy loss; BCE = binary cross-entropy loss; CE = cross-entropy loss; ASL = asymmetric focal loss.

model	loss	LR	BS	epoch	Precision per-species	Precision per-image	Recall per-species	Recall per-image
Deepbiosphere	Sampling-aware BCE	1x10^{-5}	150	8	**0.0131 [0.0036-0.0355]**	**0.0116 [0.0055-0.0294]**	0.9583 [0.5-1.0]	**1.0 [0.8889-1.0]**
Bioclim MLP	Sampling-aware BCE	1x10^{-5}	1,000	61	0.0111 [0.0023-0.0305]	0.0103 [0.0047-0.0265]	**0.9643 [0.4286-1.0]**	**1.0 [0.8627-1.0]**
TResNet	Sampling-aware BCE	1x10^{-5}	150	12	0.01 [0.0025-0.025]	0.0091 [0.0044-0.0238]	0.8958 [0.4667-1.0]	**1.0 [0.8286-1.0]**
Inception V3	CE	1x10^{-4}	100	11	0.0 [0.0-0.0]	0.0 [0.0-0.0]	0.0 [0.0-0.0]	0.0 [0.0-0.0]
Maxent	N/A	N/A	N/A	N/A	0.0048 [0.0-0.0323]	0.0 [0.0-0.0238]	0.1348 [0.0-0.566]	0.0 [0.0-0.5]
Random Forest	N/A	N/A	N/A	N/A	0.0086 [0.0-0.04]	0.0076 [0.0-0.0317]	0.3684 [0.0-0.8182]	0.2821 [0.0-0.875]
random	N/A	N/A	N/A	N/A	0.0016 [0.0005-0.0052]	0.0016 [0.0008-0.0039]	0.5 [0.4667-0.5333]	0.5 [0.4-0.6]
frequency	N/A	N/A	N/A	N/A	0.0 [0.0-0.0]	0.0 [0.0-0.0]	0.0 [0.0-0.0]	0.0 [0.0-0.0]

model	loss	LR	BS	epoch	F1 per-species	F1 per-image	mAP	Presence accuracy
Deepbiosphere	Sampling-aware BCE	1x10^{-5}	150	8	**0.0258 [0.0071-0.0685]**	**0.0229 [0.0109-0.0569]**	0.1721	**0.8918**
Bioclim MLP	Sampling-aware BCE	1x10^{-5}	1,000	61	0.0218 [0.0045-0.0592]	0.0203 [0.0094-0.0515]	0.1421	0.882
TResNet	Sampling-aware BCE	1x10^{-5}	150	12	0.0198 [0.0051-0.0479]	0.018 [0.0088-0.0463]	0.1426	0.8645
Inception V3	CE	1x10^{-4}	100	11	0.0 [0.0-0.0]	0.0 [0.0-0.0]	**0.173**	0.0013
Maxent	N/A	N/A	N/A	N/A	0.0089 [0.0-0.059]	0.0 [0.0-0.0426]	0.0366	0.2761
Random Forest	N/A	N/A	N/A	N/A	0.0166 [0.0-0.0721]	0.0152 [0.0-0.0588]	0.0642	0.3943
random	N/A	N/A	N/A	N/A	0.0031 [0.001-0.0102]	0.0031 [0.0016-0.0077]	0.0093	0.5005
frequency	N/A	N/A	N/A	N/A	0.0 [0.0-0.0]	0.0 [0.0-0.0]	0.0389	0.0656

model	loss	LR	BS	epoch	Top 1 per-image	Top 5 per-image	Top 30 per-image	Top 100 per-image
Deepbiosphere	Sampling-aware BCE	1x10^{-5}	150	8	0.044	0.1536	0.4651	**0.7613**
Bioclim MLP	Sampling-aware BCE	1x10^{-5}	1,000	61	0.034	0.1168	0.391	0.7035
TResNet	Sampling-aware BCE	1x10^{-5}	150	12	0.0366	0.1281	0.3975	0.6779
Inception V3	CE	1x10^{-4}	100	11	**0.0525**	**0.1663**	**0.4683**	0.7533
Maxent	N/A	N/A	N/A	N/A	0.0004	0.0053	0.0815	0.291
Random Forest	N/A	N/A	N/A	N/A	0.0138	0.046	0.184	0.3709
random	N/A	N/A	N/A	N/A	0.0006	0.0022	0.0142	0.0451
frequency	N/A	N/A	N/A	N/A	0.0066	0.0274	0.0846	0.1952

model	loss	LR	BS	epoch	Top 1 per-species	Top 5 per-species	Top 30 per-species	Top 100 per-species
Deepbiosphere	Sampling-aware BCE	1x10^{-5}	150	8	0.0 [0.0-0.0]	0.0 [0.0-0.0]	0.0833 [0.0-0.5]	**0.6667 [0.0-0.9333]**
Bioclim MLP	Sampling-aware BCE	1x10^{-5}	1,000	61	0.0 [0.0-0.0]	0.0 [0.0-0.0]	0.0 [0.0-0.375]	0.5 [0.0-0.8571]
TResNet	Sampling-aware BCE	1x10^{-5}	150	12	0.0 [0.0-0.0]	0.0 [0.0-0.0]	0.0 [0.0-0.3889]	0.5 [0.0-0.8]
Inception V3	CE	1x10^{-4}	100	11	0.0 [0.0-0.0]	0.0 [0.0-0.0]	**0.1111 [0.0-0.5]**	0.625 [0.0-0.9167]
Maxent	N/A	N/A	N/A	N/A	0.0 [0.0-0.0]	0.0 [0.0-0.0]	0.0 [0.0-0.0]	0.0417 [0.0-0.5]
Random Forest	N/A	N/A	N/A	N/A	0.0 [0.0-0.0]	0.0 [0.0-0.0]	0.0 [0.0-0.25]	0.2857 [0.0-0.6129]
random	N/A	N/A	N/A	N/A	0.0 [0.0-0.0]	0.0 [0.0-0.0]	0.0 [0.0-0.016]	0.0333 [0.0-0.0667]
frequency	N/A	N/A	N/A	N/A	0.0 [0.0-0.0]	0.0 [0.0-0.0]	0.0 [0.0-0.0]	0.0 [0.0-0.0]

model	loss	LR	BS	epoch	AUC$_{ROC}$	AUC$_{PRC}$	calibrated AUC$_{ROC}$	calibrated AUC$_{PRC}$
Deepbiosphere	Sampling-aware BCE	1x10^{-5}	150	8	**0.9496 [0.8896-0.9815]**	**0.0398 [0.0102-0.1074]**	**0.9452 [0.8847-0.9798]**	**0.0322 [0.0094-0.0733]**
Bioclim MLP	Sampling-aware BCE	1x10^{-5}	1,000	61	0.9346 [0.8579-0.9739]	0.0346 [0.0088-0.0979]	0.9311 [0.8483-0.972]	0.0219 [0.0064-0.0536]
TResNet	Sampling-aware BCE	1x10^{-5}	150	12	0.9268 [0.8627-0.9671]	0.0265 [0.0072-0.0787]	0.9247 [0.8579-0.965]	0.0224 [0.0072-0.0588]
Inception V3	CE	0.0001	100	11	0.9391 [0.8753-0.9755]	0.0359 [0.0098-0.0998]	0.5 [0.5-0.5366]	0.0015 [0.0004-0.0429]
Maxent	N/A	N/A	N/A	N/A	0.8825 [0.7754-0.9505]	0.018 [0.0039-0.0725]	0.8701 [0.7471-0.9455]	0.0125 [0.0031-0.051]
Random Forest	N/A	N/A	N/A	N/A	0.882 [0.7639-0.9515]	0.0237 [0.0044-0.0925]	0.8797 [0.7623-0.9493]	0.0176 [0.0035-0.0631]
random	N/A	N/A	N/A	N/A	0.4995 [0.4815-0.5174]	0.0022 [0.001-0.0064]	0.4997 [0.4834-0.5165]	0.002 [0.0009-0.0061]
frequency	N/A	N/A	N/A	N/A	0.5 [0.5-0.5]	0.0016 [0.0005-0.0052]	0.5 [0.5-0.5]	0.0009 [0.0003-0.0029]

Table S8 | Comparison of *Deepbiosphere* to baseline SDMs and previous deep-learning-based approaches

Comparison of *Deepbiosphere* to previous approaches using held-out observations uniformly sampled from across California across twenty different accuracy metrics. Cumulatively, *Deepbiosphere* had the highest accuracy on 11/20 metrics (bolded entries),

BioClim MLP on 1/20 metrics, and *Inception V3* on 5/20 metrics. Accuracies are reported for held-out observations from the uniform split of the dataset (**Fig. S4A)**. Reported statistics are median [1st quartile - 3rd quartile] calculated for the epoch of evaluation determined using early stopping on AUC_{ROC}. Abbreviations: LR = learning rate; BS = batch size; MLP = multilayer perceptron; mAP = mean average precision; ROC = receiver operating characteristic curve; AUC = area under the curve; PRC = precision-recall curve; Sampling-aware BCE = Sampling-aware binary cross-entropy loss; CE = cross-entropy loss.

model	loss	LR	BS	epoch	AUC_{ROC}	AUC_{PRC}	calibrated AUC_{ROC}	calibrated AUC_{PRC}
Deepbiosphere	Sampling-aware BCE	$1x10^{-5}$	150	8	**0.9612 [0.8964-0.9877]**	**0.0172 [0.004-0.058]**	**0.9563 [0.8792-0.9849]**	**0.0146 [0.004-0.0449]**
Bioclim MLP	Sampling-aware BCE	$1x10^{-5}$	1,000	61	0.9487 [0.8654-0.9814]	0.016 [0.0034-0.0516]	0.9425 [0.8295-0.9794]	0.0101 [0.003-0.0288]
TResNet	Sampling-aware BCE	$1x10^{-5}$	150	12	0.9413 [0.8732-0.9754]	0.0111 [0.0033-0.0443]	0.9382 [0.8624-0.9726]	0.0111 [0.003-0.033]
Inception V3	CE	$1x10^{-4}$	100	11	0.9566 [0.8964-0.985]	0.0152 [0.0045-0.0571]	0.5 [0.5-0.5]	0.0004 [0.0002-0.001]
Maxent	N/A	N/A	N/A	N/A	0.8861 [0.7502-0.9581]	0.0067 [0.0017-0.0259]	0.8695 [0.7259-0.955]	0.0046 [0.0013-0.0176]
Random Forest	N/A	N/A	N/A	N/A	0.8886 [0.7194-0.965]	0.0079 [0.0017-0.0338]	0.8858 [0.7298-0.9621]	0.0055 [0.0016-0.0225]

model	loss	LR	BS	epoch	Top 1 per-species	Top 5 per-species	Top 30 per-species	Top 100 per-species
Deepbiosphere	Sampling-aware BCE	$1x10^{-5}$	150	8	0.0 [0.0-0.0]	0.0 [0.0-0.0]	0.0 [0.0-0.0]	0.0 [0.0-0.8]
Bioclim MLP	Sampling-aware BCE	$1x10^{-5}$	1,000	61	0.0 [0.0-0.0]	0.0 [0.0-0.0]	0.0 [0.0-0.0]	0.0 [0.0-0.75]
TResNet	Sampling-aware BCE	$1x10^{-5}$	150	12	0.0 [0.0-0.0]	0.0 [0.0-0.0]	0.0 [0.0-0.0]	0.0 [0.0-0.5]
Inception V3	CE	$1x10^{-4}$	100	11	0.0 [0.0-0.0]	0.0 [0.0-0.0]	0.0 [0.0-0.0]	0.0 [0.0-0.75]
Maxent	N/A	N/A	N/A	N/A	0.0 [0.0-0.0]	0.0 [0.0-0.0]	0.0 [0.0-0.125]	0.0 [0.0-0.6667]
Random Forest	N/A	N/A	N/A	N/A	0.0 [0.0-0.0]	0.0 [0.0-0.0]	0.0 [0.0-0.5]	**0.3333 [0.0-1.0]**

model	loss	LR	BS	epoch	Precision per-species	Recall per-species	F1 per-species
Deepbiosphere	Sampling-aware BCE	$1x10^{-5}$	150	8	**0.0054 [0.0-0.0168]**	0.6111 [0.0-1.0]	**0.0108 [0.0-0.0325]**
Bioclim MLP	Sampling-aware BCE	$1x10^{-5}$	1,000	61	0.0039 [0.0-0.0141]	**0.6667 [0.0-1.0]**	0.0078 [0.0-0.0278]
TResNet	Sampling-aware BCE	$1x10^{-5}$	150	12	0.0039 [0.0-0.0121]	0.5294 [0.0-0.9722]	0.0076 [0.0-0.024]
Inception V3	CE	$1x10^{-4}$	100	11	0.0 [0.0-0.0]	0.0 [0.0-0.0]	0.0 [0.0-0.0]
Maxent	N/A	N/A	N/A	N/A	0.0 [0.0-0.007]	0.0 [0.0-0.6667]	0.0 [0.0-0.0139]
Random Forest	N/A	N/A	N/A	N/A	0.0022 [0.0-0.0102]	0.2727 [0.0-1.0]	0.0041 [0.0-0.0201]

Table S9 | Comparison of *Deepbiosphere* to baseline SDMs on rarest species

Comparison of *Deepbiosphere* to previous approaches for species with fewer than 100 observations in the original dataset before neighbor imputation (529 out of 2,221 species), using held-out observations uniformly sampled from across California (**Fig S4A**) across eleven different accuracy metrics. Cumulatively, *Deepbiosphere* had the highest accuracy on all but two metrics (bolded entries), *BioClim MLP* on 1/11 metrics, and *Random Forest* on 1/11 metrics. While for most metrics accuracy is lower, intriguingly AUC_{ROC} increased for the deep learning-based models (*Deepbiosphere, Bioclim MLP, Inception V3*) compared to the entire dataset. Reported statistics are median [1st quartile - 3rd quartile] calculated for the epoch of evaluation determined using early stopping on AUC_{ROC}. Abbreviations: LR = learning rate; BS = batch size; MLP = multilayer perceptron; mAP = mean average precision; ROC = receiver operating characteristic curve; AUC = area under the curve; PRC = precision-recall curve; Sampling-aware BCE = sampling-aware binary cross-entropy loss; CE = cross-entropy loss.

model	loss	LR	BS	epoch	Precision per-species	Precision per-image	Recall per-species	Recall per-image
Deepbiosphere	Sampling-aware BCE	$1x10^{-5}$	150	8	**0.0219 [0.0183-0.0287]**	**0.0554 [0.037-0.0739]**	**0.5865 [0.5377-0.6388]**	**0.8571 [0.8237-0.8785]**
Bioclim MLP	Sampling-aware BCE	$1x10^{-5}$	1,000	61	0.0129 [0.0102-0.0169]	0.0451 [0.0306-0.0637]	0.4536 [0.381-0.496]	0.8091 [0.756-0.8512]
Maxent	N/A	N/A	N/A	N/A	0.0045 [0.0002-0.0108]	0.0237 [0.0221-0.0318]	0.1541 [0.0034-0.5063]	0.4273 [0.3202-0.6838]
Random Forest	N/A	N/A	N/A	N/A	0.0073 [0.0034-0.0121]	0.0231 [0.0192-0.0283]	0.4129 [0.0908-0.6874]	0.5714 [0.4023-0.7447]
Frequency	N/A	N/A	N/A	N/A	0.0 [0.0-0.0]	0.0526 [0.0125-0.0937]	0.0 [0.0-0.0]	0.0801 [0.0069-0.0933]
**model cont'd**	**loss**	**LR**	**BS**	**epoch**	**F1 per-species**	**F1 per-image**	**mAP**	**Presence accuracy**
Deepbiosphere	Sampling-aware BCE	$1x10^{-5}$	150	8	**0.0414 [0.0348-0.0513]**	**0.1034 [0.0703-0.1359]**	**0.2181 [0.1934-0.2558]**	**0.8425 [0.8262-0.8706]**
Bioclim MLP	Sampling-aware BCE	$1x10^{-5}$	1,000	61	0.0242 [0.0196-0.0302]	0.0849 [0.059-0.1181]	0.1596 [0.1357-0.1838]	0.7856 [0.753-0.8179]
Maxent	N/A	N/A	N/A	N/A	0.0088 [0.0003-0.0207]	0.0439 [0.0387-0.0604]	0.055 [0.0515-0.0595]	0.4268 [0.3619-0.6443]
Random Forest	N/A	N/A	N/A	N/A	0.0137 [0.0064-0.0232]	0.0435 [0.0361-0.0541]	0.0636 [0.0533-0.0731]	0.5113 [0.4369-0.6873]
Frequency	N/A	N/A	N/A	N/A	0.0 [0.0-0.0]	0.0739 [0.0096-0.0899]	0.0994 [0.0626-0.1042]	0.103 [0.0701-0.1158]
**model cont'd**	**loss**	**LR**	**BS**	**epoch**	**Top 1 per-image**	**Top 5 per-image**	**Top 30 per-image**	**Top 100 per-image**
Deepbiosphere	Sampling-aware BCE	$1x10^{-5}$	150	8	**0.0262 [0.0237-0.0296]**	**0.1051 [0.1013-0.1106]**	**0.3771 [0.3721-0.3902]**	**0.6803 [0.6693-0.691]**
Bioclim MLP	Sampling-aware BCE	$1x10^{-5}$	1,000	61	0.0152 [0.0129-0.0192]	0.0667 [0.0631-0.0716]	0.2789 [0.2665-0.2866]	0.5482 [0.5313-0.5803]
Maxent	N/A	N/A	N/A	N/A	0.0004 [0.0001-0.0018]	0.0037 [0.0019-0.0073]	0.0455 [0.04-0.06]	0.1862 [0.1582-0.1942]
Random Forest	N/A	N/A	N/A	N/A	0.0036 [0.0025-0.0047]	0.0209 [0.0128-0.023]	0.0974 [0.0747-0.1096]	0.2234 [0.2013-0.282]
Frequency	N/A	N/A	N/A	N/A	0.0091 [0.005-0.0111]	0.036 [0.0297-0.0429]	0.133 [0.0995-0.1495]	0.3008 [0.2161-0.3123]
**model cont'd**	**loss**	**LR**	**BS**	**epoch**	**Top 1 per-species**	**Top 5 per-species**	**Top 30 per-species**	**Top 100 per-species**
Deepbiosphere	Sampling-aware BCE	$1x10^{-5}$	150	8	0.0 [0.0-0.0]	0.0 [0.0-0.0]	0.0 [0.0-0.0]	**0.2242 [0.1613-0.292]**
Bioclim MLP	Sampling-aware BCE	$1x10^{-5}$	1,000	61	0.0 [0.0-0.0]	0.0 [0.0-0.0]	0.0 [0.0-0.0]	0.0378 [0.0-0.07]
Maxent	N/A	N/A	N/A	N/A	0.0 [0.0-0.0]	0.0 [0.0-0.0]	0.0 [0.0-0.0]	0.0 [0.0-0.0]
Random Forest	N/A	N/A	N/A	N/A	0.0 [0.0-0.0]	0.0 [0.0-0.0]	0.0 [0.0-0.0]	0.0288 [0.0-0.058]
Frequency	N/A	N/A	N/A	N/A	0.0 [0.0-0.0]	0.0 [0.0-0.0]	0.0 [0.0-0.0]	0.0 [0.0-0.0]
**model cont'd**	**loss**	**LR**	**BS**	**epoch**	**AUC_{ROC}**	**AUC_{PRC}**	**calibrated AUC_{ROC}**	**calibrated AUC_{PRC}**
Deepbiosphere	Sampling-aware BCE	$1x10^{-5}$	150	8	**0.8682 [0.8388-0.8839]**	**0.0365 [0.0339-0.0421]**	**0.8425 [0.8164-0.8718]**	**0.0338 [0.0302-0.0387]**
Bioclim MLP	Sampling-aware BCE	$1x10^{-5}$	1,000	61	0.8025 [0.7722-0.8214]	0.0279 [0.0261-0.0307]	0.7645 [0.7259-0.7901]	0.0196 [0.0171-0.0222]
Maxent	N/A	N/A	N/A	N/A	0.7339 [0.7071-0.7713]	0.0207 [0.0195-0.0235]	0.7223 [0.6932-0.7525]	0.0146 [0.0132-0.0174]
Random Forest	N/A	N/A	N/A	N/A	0.7056 [0.6945-0.7576]	0.0219 [0.0182-0.0251]	0.7072 [0.6957-0.76]	0.0166 [0.0131-0.0196]
Frequency	N/A	N/A	N/A	N/A	0.5 [0.5-0.5]	0.0045 [0.0038-0.0052]	0.5 [0.5-0.5]	0.0023 [0.0019-0.0028]

Table S10 | Comparison of *Deepbiosphere* to baseline SDMs across spatial cross-validation bands.
Comparison of *Deepbiosphere* to baseline SDMs using a ten-fold spatial cross-validation approach across twenty different accuracy metrics. *Deepbiosphere* exhibited superior performance on all metrics (bolded entries). Furthermore, while all approaches in general see a reduction in performance compared to the uniform data split (**Table S8**), in general *Deepbiosphere* exhibits a less steep drop in accuracy, and even improves for some metrics. Reported statistics are median [1st quartile - 3rd quartile] for the median value of each band from the ten-fold spatial cross-validation (**Fig. S4B**). Accuracies were calculated using the early stopping epoch on AUC_{ROC} from the uniform test split. MLP = multilayer perceptron; LR = learning rate; BS = batch size; Sampling-aware BCE = Sampling-aware binary cross-entropy loss; mAP = mean average precision; ROC = receiver operating characteristic curve; AUC = area under the curve; PRC = precision-recall curve.

**Redwoods case study location:** (41.209, -124.01)		**Oaks case study location:** (34.533, -120.17)		
Redwoods example locations	Grove – Park	Oaks example locations	Species	Calflora ID
(40.3527, -123.9894)	Bull Creek Flats – Humboldt Redwoods	(36.108322, -120.561226)	*Quercus lobata*	po68973
(41.7564, -124.1087)	Grove of Titans – Jedediah Smith Redwoods State Park	(36.151438, -120.770939)	*Quercus lobata*	po68984
(40.6554, -124.0998)	Elk River Trail Grove – Headwaters Preserve	(36.635690, -121.242532)	*Quercus lobata*	po122185

Table S11 | Site details for individual species case studies and human annotation experiments
Two locations within both species' predicted range on Calscape (https://calscape.org) were selected as case studies. For the redwoods case study (left-hand side), example locations were chosen from Calflora (64) based on known remaining old-growth redwood groves and Tall Trees Grove in Redwoods National and State Parks was selected as the case study location, including a known Calflora redwood observation (first row). For the oaks case study (right-hand side), a list of candidate locations was generated from Calflora (64) *Quercus lobata* occurrence records, with the ultimate case study site being selected from a region with multiple observed oaks from an undersampled region in the uniform dataset. For each site, the most centered NAIP imagery tile was selected as the extent of the case study. Each NAIP imagery tile is approximately 5 x 6 km in extent.

model	LR	BS	epoch	AUC$_{PRC}$	AUC$_{ROC}$	Recall	F1	Top 100$_{spp}$	AUC$_{PRC}$	AUC$_{ROC}$	Recall	F1	Top 100$_{spp}$
				Sequoia sempervirens - tested with 1,292 observations									
Deepbiosphere$_{10}$	1x10^{-4}	150	5	**0.3832**	**0.857**	**0.7957**	**0.4855**	**0.9771**					
Bioclim MLP$_{10}$	1x10^{-5}	1000	61	0.1939	0.6814	0.0426	0.0545	0.0534					
Maxent$_{10}$	N/A	N/A	N/A	0.3283	0.8258	0.0108	0.0182	0.0					
Random Forest$_{10}$	N/A	N/A	N/A	0.3384	0.8437	0.0	0.0	0.0					
**Species associated with mature redwood forest**													
				Oxalis oregana - tested with 1,680 observations					*Struthiopteris spicant*- tested with 1,157 observations				
Deepbiosphere$_{10}$	1x10^{-4}	150	5	**0.4125**	**0.8286**	**0.6863**	**0.4989**	**0.8947**	**0.3507**	**0.8625**	**0.7373**	**0.7373**	**0.9277**
Bioclim MLP$_{10}$	1x10^{-5}	1000	61	0.3297	0.7852	0.8083	0.4957	0.5639	0.2633	0.7816	0.6612	0.6612	0.4337
Maxent$_{10}$	N/A	N/A	N/A	0.3783	0.7871	0.0345	0.0514	0.0	0.2777	0.7468	0.2377	0.2377	0.0241
Random Forest$_{10}$	N/A	N/A	N/A	0.3631	0.7749	0.0083	0.0164	0.0	0.2842	0.7517	0.8211	0.8211	0.7711
**Species associated with secondary growth redwood forest**													
				Rubus ursinus - tested with 687 observations					*Viola sempervirens* - tested with 1,309 observations				
Deepbiosphere$_{10}$	1x10^{-4}	150	5	**0.1857**	**0.7866**	0.9185	**0.2099**	**0.8077**	**0.3783**	**0.8421**	**0.8648**	0.4587	**0.8875**
Bioclim MLP$_{10}$	1x10^{-5}	1000	61	0.1467	0.72	**0.9753**	0.1897	0.6923	0.2074	0.6504	0.3759	0.274	0.2875
Maxent$_{10}$	N/A	N/A	N/A	0.0947	0.4853	0.0146	0.0215	0.0	0.3124	0.6792	0.5286	**0.4671**	0.2375
Random Forest$_{10}$	N/A	N/A	N/A	0.089	0.4857	0.0	0.0	0.0	0.2682	0.6641	0.1467	0.1829	0.05
**Species associated both with mature redwood forest**													
				Polystichum munitum - tested with 1,804 observations					*Vaccinium ovatum* - tested with 1,771 observations				
Deepbiosphere$_{10}$	1x10^{-4}	150	5	**0.5717**	**0.8866**	**0.9429**	**0.5325**	**0.96**	**0.5137**	**0.8635**	**0.9989**	**0.5441**	0.9802
Bioclim MLP$_{10}$	1x10^{-5}	1000	61	0.3603	0.7846	0.9191	0.5296	0.63	0.3731	0.8113	1	0.5234	**0.9901**
Maxent$_{10}$	N/A	N/A	N/A	0.3623	0.6771	0.1142	0.1758	0	0.3521	0.6798	0.2778	0.3386	0
Random Forest$_{10}$	N/A	N/A	N/A	0.3613	0.6838	0.1258	0.1891	0	0.3273	0.6985	0.122	0.1788	0.0099

Table S12 | Accuracy of various SDMs on redwood + understory species in northern California
Five accuracy metrics for the seven species from the Redwoods National and State Parks case study using held-out dataset observations from inside the tenth spatial cross-validation block (**Fig. S4B**). While accuracy varies across species, *Deepbiosphere* exhibits superior performance for all species and all metrics save three (*R. ursinus* recall, *V. sempervirens* F1, *V. ovatum* Top 100), highlighting how including remote sensing as a predictor improves performance of species distribution models in unseen regions. The *Inception$_{unif}$* SDM baseline was left out from this analysis as the model was trained using anywhere from 95% to 100% of all held-out test observations in this spatial band. The number of neighbor-imputed observations used to calculate the accuracies is denoted alongside the species name. Reported statistics were calculated for the epoch of evaluation determined using early stopping on AUC$_{ROC}$ using the uniform data split. MLP = multilayer perceptron; ROC = receiver operating characteristic curve; AUC = area under the curve; *spp* = per-species.

				Ceanothus cuneatus - tested with 1,682 observations						*Quercus lobata* - tested with 4,460 observations					
model	LR	BS	epoch	% seen	AUC$_{PRC}$	AUC$_{ROC}$	Recall	F1	Top 100$_{spp}$	% seen	AUC$_{PRC}$	AUC$_{ROC}$	Recall	F1	Top 100$_{spp}$
Deepbiosphere$_3$	1x10^{-4}	150	5	0.0%	**0.0396**	**0.7287**	0.1843	**0.0509**	**0.4821**	0.0%	**0.1163**	**0.7893**	0.6415	**0.2062**	**0.7626**
Bioclim MLP$_3$	1x10^{-5}	1000	61	0.0%	0.0275	0.6622	0.1379	0.0263	0.3036	0.0%	0.0673	0.6951	**0.92**	0.1245	0.7071
Maxent$_3$	N/A	N/A	N/A	0.0%	0.0222	0.6028	**0.201**	0.0371	0	0.0%	0.0812	0.7092	0.5511	0.1541	0.1313
Random Forest$_3$	N/A	N/A	N/A	0.0%	0.0279	0.6949	0.085	0.0406	0.0357	0.0%	0.0413	0.5154	0.0397	0.0228	0.0051

				Adenostoma fasciculatum - tested with 16,680 observations						*Bromus diandrus* - tested with 8,793 observations					
model	LR	BS	epoch	% seen	AUC$_{PRC}$	AUC$_{ROC}$	Recall	F1	Top 100$_{spp}$	% seen	AUC$_{PRC}$	AUC$_{ROC}$	Recall	F1	Top 100$_{spp}$
Deepbiosphere$_3$	1x10^{-4}	150	5	0.0%	**0.2855**	**0.7243**	0.6472	**0.3934**	**0.7147**	0.0%	**0.1338**	0.637	0.5169	0.2206	**0.3946**
Bioclim MLP$_3$	1x10^{-5}	1000	61	0.0%	0.2717	0.7083	**0.8438**	0.379	0.6006	0.0%	0.122	0.6693	**0.9883**	0.1843	0.2378
Maxent$_3$	N/A	N/A	N/A	0.0%	0.2829	0.6992	0.3058	0.3088	0.0157	0.0%	0.1331	**0.6716**	0.6659	**0.2211**	0.0
Random Forest$_3$	N/A	N/A	N/A	0.0%	0.2273	0.6332	0.2974	0.2304	0.0713	0.0%	0.1097	0.6351	0.9377	0.203	0.1189

				Quercus berberidifolia - tested with 5,034 observations						*Arctostaphylos glandulosa* - tested with 2,091 observations					
model	LR	BS	epoch	% seen	AUC$_{PRC}$	AUC$_{ROC}$	Recall	F1	Top 100$_{spp}$	% seen	AUC$_{PRC}$	AUC$_{ROC}$	Recall	F1	Top 100$_{spp}$
Deepbiosphere$_3$	1x10^{-4}	150	5	0.0%	**0.0936**	**0.7301**	0.2978	**0.1298**	**0.3871**	0.0%	**0.0711**	**0.79**	0.197	0.1328	**0.3644**
Bioclim MLP$_3$	1x10^{-5}	1000	61	0.0%	0.0674	0.6376	0.4543	0.1246	0.1774	0.0%	0.0603	0.7816	0.133	0.0761	0.1695
Maxent$_3$	N/A	N/A	N/A	0.0%	0.0671	0.6231	0.2086	0.1036	0.1371	0.0%	0.07	0.7129	0.2195	**0.1587**	0.0339
Random Forest$_3$	N/A	N/A	N/A	0.0%	0.0876	0.7255	**0.4994**	0.136	0.1935	0.0%	0.0452	0.6102	**0.3046**	0.0982	0.1102

Table S13 | Accuracy of various SDMs on chaparral indicator species in southern California

Five accuracy metrics for the six species from the chaparral case study using held-out dataset observations from inside the third spatial cross-validation block (**Fig. S4B**). While the accuracy varies across species, *Deepbiosphere* still exhibits superior performance on at least two metrics for all species. Climate-based SDMs performed best for *B. diandrus*,—a wind-dispersed invasive annual grass—and *A. glandulosa*, an chaparral endemic shrub. *Deepbiosphere's* performance was likely lower on average for chaparral species compared to redwoods-associated species because the third spatial cross-validation block has nearly 100,000 fewer training examples than the tenth (546,621 vs. 642,661 observations), leaving fewer observations to sufficiently extract patterns of chaparral species' distributions from *Deepbiosphere's* remote sensing data.The *Inception*$_{unif}$ SDM baseline was left out from this analysis as the model was trained using anywhere from 98% to 100% of all held-out test observations in this spatial band. The number of neighbor-imputed observations used to calculate the accuracies is denoted alongside the species name. Reported statistics were calculated for the epoch of evaluation determined using early stopping on AUC$_{ROC}$ using the uniform data split. MLP = multilayer perceptron; ROC = receiver operating characteristic curve; AUC = area under the curve; *spp* = per-species.

Supplemental References

1. E. Cole, *et al.*, The GeoLifeCLEF 2020 Dataset. *arXiv [cs.CV]* (2020).

2. Global Biodiversity Information Facility, GBIF Occurrence Download (2022) https:/doi.org/10.15468/dl.gt624q (July 30, 2022).

3. K. A. Uyeda, D. A. Stow, C. H. Richart, Assessment of volunteered geographic information for vegetation mapping. *Environ. Monit. Assess.* **192**, 554 (2020).

4. S. Gaiji, *et al.*, Content assessment of the primary biodiversity data published through GBIF network: Status, challenges and potentials. *Biodivers. Inf.* **8** (2013).

5. Y. L. Dupont, K. Trøjelsgaard, J. M. Olesen, Scaling down from species to individuals: a flower-visitation network between individual honeybees and thistle plants. *Oikos* **120**, 170–177 (2011).

6. M. S. Wisz, *et al.*, The role of biotic interactions in shaping distributions and realised assemblages of species: implications for species distribution modelling. *Biol. Rev. Camb. Philos. Soc.* **88**, 15–30 (2013).

7. R. G. Pearson, T. P. Dawson, Predicting the impacts of climate change on the distribution of species: are bioclimate envelope models useful? *Glob. Ecol. Biogeogr.* **12**, 361–371 (2003).

8. Earth Resources Observation and Science (EROS) Center, National Agriculture Imagery Program (NAIP) (2012) https:/doi.org/10.5066/F7QN651G (2022).

9. A. E. Maxwell, T. A. Warner, B. C. Vanderbilt, C. A. Ramezan, Land cover classification and feature extraction from national agriculture imagery program (NAIP) orthoimagery: A review. *Photogramm. Eng. Remote Sensing* **83**, 737–747 (2017).

10. Y. Zhang, *et al.*, Prediction of Soil Organic Carbon based on Landsat 8 Monthly NDVI Data for the Jianghan Plain in Hubei Province, China. *Remote Sensing* **11**, 1683 (2019).

11. S. E. Fick, R. J. Hijmans, WorldClim 2: new 1-km spatial resolution climate surfaces for global land areas. *Int. J. Climatol.* **37**, 4302–4315 (2017).

12. A. Cotrina Sánchez, *et al.*, Biogeographic Distribution of Cedrela spp. Genus in Peru Using MaxEnt Modeling: A Conservation and Restoration Approach. *Diversity* **13**, 261 (2021).

13. L. Poggio, E. Simonetti, A. Gimona, Enhancing the WorldClim data set for national and regional applications. *Sci. Total Environ.* **625**, 1628–1643 (2018).

14. R. Benestad, Downscaling climate information. *Oxford research encyclopedia of climate science* https:/doi.org/10.1093/acrefore/9780190228620.001.0001/acrefore-9780190228620-e-27.

15. M. R. Trivedi, P. M. Berry, M. D. Morecroft, T. P. Dawson, Spatial scale affects bioclimate model projections of climate change impacts on mountain plants. *Glob. Chang. Biol.* **14**, 1089–1103 (2008).

16. B. J. Enquist, *et al.*, The commonness of rarity: Global and future distribution of rarity across land plants. *Sci Adv* **5**, eaaz0414 (2019).

17. N. Japkowicz, S. Stephen, The class imbalance problem: A systematic study1. *Intell. Data Anal.* **6**, 429–449 (2002).

18. M. Zhu, *et al.*, Class Weights Random Forest Algorithm for Processing Class Imbalanced Medical Data. *IEEE Access* **6**, 4641–4652 (2018).

19. P. Chu, X. Bian, S. Liu, H. Ling, Feature Space Augmentation for Long-Tailed Data. *arXiv [cs.CV]* (2020).

20. A. T. Taylor, T. Hafen, C. T. Holley, A. González, J. M. Long, Spatial sampling bias and model complexity in stream-based species distribution models: A case study of Paddlefish (Polyodon spathula) in the Arkansas River basin, USA. *Ecol. Evol.* **10**, 705–717 (2020).

21. E. A. Freeman, G. G. Moisen, A comparison of the performance of threshold criteria for binary classification in terms of predicted prevalence and kappa. *Ecol. Modell.* **217**, 48–58 (2008).

22. C. R. Lawson, J. A. Hodgson, R. J. Wilson, S. A. Richards, Prevalence, thresholds and the performance of presence-absence models. *Methods Ecol. Evol.* **5**, 54–64 (2014).

23. F. Pedregosa, *et al.*, " Scikit-learn: Machine Learning in Python," Journal of Machine Learning Research, vol. 12, p (2011).

24. A. Kubany, *et al.*, Comparison of state-of-the-art deep learning APIs for image multi-label classification using semantic metrics. *Expert Syst. Appl.* **161**, 113656 (2020).

25. A. Jiménez-Valverde, J. M. Lobo, Threshold criteria for conversion of probability of species presence to either–or presence–absence. *Acta Oecol.* **31**, 361–369 (2007).

26. C. F. Dormann, *et al.*, Correlation and process in species distribution models: bridging a dichotomy. *J. Biogeogr.* **39**, 2119–2131 (2012).

27. H. Hamilton, *et al.*, Increasing taxonomic diversity and spatial resolution clarifies opportunities for protecting US imperiled species. *Ecol. Appl.* **32**, e2534 (2022).

28. M. W. Tobler, *et al.*, Joint species distribution models with species correlations and imperfect detection. *Ecology* **100**, e02754 (2019).

29. J. Roughgarden, The Fundamental and Realized Niche of a Solitary Population. *Am. Nat.* **108**, 232–235 (1974).

30. W. Godsoe, J. Jankowski, R. D. Holt, D. Gravel, Integrating Biogeography with Contemporary Niche Theory. *Trends Ecol. Evol.* **32**, 488–499 (2017).

31. K. C. Rosenblad, D. L. Perret, D. F. Sax, Niche syndromes reveal climate-driven extinction threat to island endemic conifers. *Nat. Clim. Chang.* **9**, 627–631 (2019).

32. E. A. Beever, *et al.*, Improving Conservation Outcomes with a New Paradigm for Understanding Species' Fundamental and Realized Adaptive Capacity. *Conserv. Lett.* **9**, 131–137 (2016).

33. B. G. Baldwin, *et al.*, Species richness and endemism in the native flora of California. *Am. J. Bot.* **104**, 487–501 (2017).

34. Y. Lecun, L. Bottou, Y. Bengio, P. Haffner, Gradient-based learning applied to document recognition. *Proc. IEEE* **86**, 2278–2324 (1998).

35. A. Krizhevsky, I. Sutskever, G. E. Hinton, ImageNet classification with deep convolutional neural

networks. *Commun. ACM* **60**, 84–90 (2017).

36. O. Ronneberger, P. Fischer, T. Brox, U-Net: Convolutional Networks for Biomedical Image Segmentation in *Medical Image Computing and Computer-Assisted Intervention – MICCAI 2015*, (Springer International Publishing, 2015), pp. 234–241.

37. N. Jean, *et al.*, Combining satellite imagery and machine learning to predict poverty. *Science* **353**, 790–794 (2016).

38. T. Ridnik, *et al.*, TResNet: High Performance GPU-Dedicated Architecture. *arXiv [cs.CV]* (2020).

39. T. Ridnik, *et al.*, TResNet: High Performance GPU-Dedicated Architecture in *2021 IEEE Winter Conference on Applications of Computer Vision (WACV)*, (IEEE, 2021), pp. 1400–1409.

40. M. Seeland, M. Rzanny, D. Boho, J. Wäldchen, P. Mäder, Image-based classification of plant genus and family for trained and untrained plant species. *BMC Bioinformatics* **20**, 1–13 (2019).

41. E. Cole, *et al.*, Spatial Implicit Neural Representations for Global-Scale Species Mapping in *International Conference on Machine Learning*, (2023).

42. B. Deneu, M. Servajean, P. Bonnet, F. Munoz, A. Joly, Participation of LIRMM/Inria to the GeoLifeCLEF 2020 challenge (2020).

43. E. Ben-Baruch, *et al.*, Asymmetric Loss For Multi-Label Classification. *arXiv [cs.CV]* (2020).

44. C. Botella, A. Joly, P. Monestiez, P. Bonnet, F. Munoz, Bias in presence-only niche models related to sampling effort and species niches: Lessons for background point selection. *PLoS One* **15**, e0232078 (2020).

45. B. Deneu, *et al.*, Convolutional neural networks improve species distribution modelling by capturing the spatial structure of the environment. *PLoS Comput. Biol.* **17**, e1008856 (2021).

46. B. Deneu, M. Servajean, P. Bonnet, F. Munoz, A. Joly, Participation of LIRMM / Inria to the GeoLifeCLEF 2020 challenge (2020) (February 22, 2022).

47. A. Norberg, *et al.*, A comprehensive evaluation of predictive performance of 33 species distribution models at species and community levels. *Ecol. Monogr.* **89**, e01370 (2019).

48. R. J. Hijmans, S. Phillips, J. Leathwick, J. Elith, dismo: Species distribution modeling. *R package version* **1**, 1–1 (2017).

49. R. Valavi, G. Guillera-Arroita, J. J. Lahoz-Monfort, J. Elith, Predictive performance of presence-only species distribution models: a benchmark study with reproducible code. *Ecological Monographs* **92** (2022).

50. C. J. Battey, P. L. Ralph, A. D. Kern, Predicting geographic location from genetic variation with deep neural networks. *Elife* **9** (2020).

51. K. A. Stumpf, Cogan Technology, and Kier Associates, "Vegetation mapping and classification project: Redwood National and State Parks, California" (National Park Service, 2017).

52. C. R. Keyes, E. K. Teraoka, Structure and Composition of Old-Growth and Unmanaged Second-Growth Riparian Forests at Redwood National Park, USA. *For. Trees Livelihoods* **5**, 256–268

(2014).

53. U.S. Forest Service, Existing Vegetation: Region 5 -Zone 7, South Coast (2018) https:/doi.org/https://data.fs.usda.gov/geodata/edw/datasets.php (2023).

54. M. Slaton, "South Coast and Montane Ecological Province CALVEG Zone 7 Vegetation Description" (USDA Forest Service, Pacific Southwest, 2009) (March 13, 2023).

55. A. Siefert, C. Ravenscroft, M. D. Weiser, N. G. Swenson, Functional beta-diversity patterns reveal deterministic community assembly processes in eastern North American trees. *Glob. Ecol. Biogeogr.* **22**, 682–691 (2013).

56. M. Večeřa, *et al.*, Alpha diversity of vascular plants in European forests. *J. Biogeogr.* **46**, 1919–1935 (2019).

57. Golden Gate National Parks Conservancy, Tamalpais Lands Collaborative (One Tam), Aerial Information Systems, Tukman Geospatial LLC, Marin County Fine Scale Vegetation Map (2021) (August 15, 2022).

58. K. Jordahl, GeoPandas: Python tools for geographic data. *URL: https://github. com/geopandas/geopandas*.

59. F. Osorio, R. Vallejos, F. Cuevas, SpatialPack: Package for analysis of spatial data. *R package version 0.2–3*.

60. E. N. Stavros, *et al.*, Unprecedented remote sensing data over King and Rim megafires in the Sierra Nevada Mountains of California. *Ecology* **97**, 3244 (2016).

61. J. E. Keeley, Fire intensity, fire severity and burn severity: a brief review and suggested usage. *Int. J. Wildland Fire* **18**, 116–126 (2009).

62. P. Dutilleul, P. Clifford, S. Richardson, D. Hemon, Modifying the t Test for Assessing the Correlation Between Two Spatial Processes. *Biometrics* **49**, 305–314 (1993).

63. T. Yep, *torchinfo* (2022) (April 17, 2023).

64. Calflora: Information on California plants for education, research and conservation. [web application]. 2024. Berkeley, California: The Calflora Database [a non-profit organization]. Available: https://www.calflora.org/ (Accessed: Mar 14, 2024).

65. Soil Survey Staff, Natural Resources Conservation Service, United States Department of Agriculture. U.S. General Soil Map (STATSGO2). Available online. Accessed (March/10/2024).

66. H. Safford, J. Miller. An updated database of serpentine endemism in the California flora. *Madroño* (January 10, 2020).
